# Optimal control strategies of SARS-CoV-2 Omicron supported by invasive and dynamic models

**DOI:** 10.1186/s40249-022-01039-y

**Published:** 2022-11-26

**Authors:** Jia Rui, Jin-Xin Zheng, Jin Chen, Hongjie Wei, Shanshan Yu, Zeyu Zhao, Xin-Yi Wang, Mu-Xin Chen, Shang Xia, Ying Zhou, Tianmu Chen, Xiao-Nong Zhou

**Affiliations:** 1 State Key Laboratory of Molecular Vaccinology and Molecular Diagnostics, School of Public Health, Xiamen University, Xiamen, People’s Republic of China; 2 Department of Nephrology, Institute of Nephrology, Ruijin Hospital, Shanghai Jiao Tong University School of Medicine, Shanghai, 200025 People’s Republic of China; 3 National Institute of Parasitic Diseases at Chinese Center for Disease Control and Prevention, WHO Collaborating Centre for Tropical Diseases, NHC Key Laboratory of Parasites and Vectors Biology of China, Shanghai, 200025 People’s Republic of China; 4 School of Global Health, Chinese Center for Tropical Diseases Research, Shanghai Jiao Tong University School of Medicine, Shanghai, 200025 People’s Republic of China

**Keywords:** SARS-CoV-2, Omicron variant, Biological invasive theory, Dynamic models, Optimal combination of interventions, Spatial spillover risk

## Abstract

**Background:**

There is a raising concern of a higher infectious Omicron BA.2 variant and the latest BA.4, BA.5 variant, made it more difficult in the mitigation process against COVID-19 pandemic. Our study aimed to find optimal control strategies by transmission of dynamic model from novel invasion theory.

**Methods:**

Based on the public data sources from January 31 to May 31, 2022, in four cities (Nanjing, Shanghai, Shenzhen and Suzhou) of China. We segmented the theoretical curves into five phases based on the concept of biological invasion. Then, a spatial autocorrelation analysis was carried out by detecting the clustering of the studied areas. After that, we choose a mathematical model of COVID-19 based on system dynamics methodology to simulate numerous intervention measures scenarios. Finally, we have used publicly available migration data to calculate spillover risk.

**Results:**

Epidemics in Shanghai and Shenzhen has gone through the entire invasion phases, whereas Nanjing and Suzhou were all ended in the establishment phase. The results indicated that *Rt* value and public health and social measures (PHSM)-index of the epidemics were a negative correlation in all cities, except Shenzhen. The intervention has come into effect in different phases of invasion in all studied cities. Until the May 31, most of the spillover risk in Shanghai remained above the spillover risk threshold (18.81–303.84) and the actual number of the spillovers (0.94–74.98) was also increasing along with the time. Shenzhen reported Omicron cases that was only above the spillover risk threshold (17.92) at the phase of outbreak, consistent with an actual partial spillover. In Nanjing and Suzhou, the actual number of reported cases did not exceed the spillover alert value.

**Conclusions:**

Biological invasion is positioned to contribute substantively to understanding the drivers and mechanisms of the COVID-19 spread and outbreaks. After evaluating the spillover risk of cities at each invasion phase, we found the dynamic zero-COVID strategy implemented in four cities successfully curb the disease epidemic peak of the Omicron variant, which was highly correlated to the way to perform public health and social measures in the early phases right after the invasion of the virus.

**Graphical Abstract:**

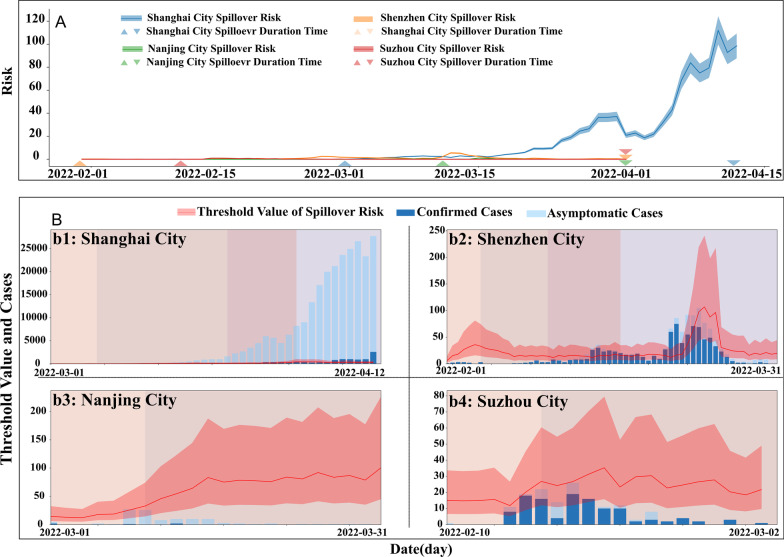

**Supplementary Information:**

The online version contains supplementary material available at 10.1186/s40249-022-01039-y.

## Background

During the coronavirus disease 2019 (COVID-19) pandemic, several variants of the severe acute respiratory syndrome coronavirus 2 (SARS-CoV-2) have been found. B.1.1.529 (Omicron) variant was first detected in a sample collected in Botswana on November 11, 2021, and first reported by South Africa on November 24, 2021 [1]. The SARS-CoV-2 Omicron variant has five major sublineages, including BA.1, BA.2, BA.3, BA.4, and BA.5, while BA.1 is a transient lineage that is rapidly replaced by the Omicron sublineage BA.2. By March 2022, Omicron BA.2 variant has been identified in 133 countries and has been the most prevalent lineage globally, representing 85% of variant cases reported in late March, 2022 [2]. Outbreaks of Omicron BA.2 had also occurred in several regions of China, such as Guangdong Province, Shanghai City, Jilin Province, and Hong Kong Special Administrative Region [3–6].

A concern has been raised based on the facts that higher infectivity of Omicron BA.2 variant, decreased effectiveness of current vaccines, uncertainty of therapeutic monoclonal antibodies and antiviral drugs for Omicron BA.2 [7–9], which create more challenges in the mitigation process against COVID-19 pandemic. The original strain of SARS-CoV-2 has a basic reproduction number (*R*_0_) of 2.5, while the Delta variant (B.1.617.2) has a *R*_0_ of just under 7, and *R*_0_ of Omicron BA.2 could be as high as 10 [10]. Even milder average clinical presentations may be offset by increased infection rates in the Omicron variant, with the potential for considerable social disruption due to disease, lost productivity, and suffering, and additional strain on health care systems due to staffing shortages [11]. Once it has invaded a city, it is difficult to control the spread of Omicron BA.2, especially where there is a high population density, e.g., Shanghai City. However, there is little quantitative analysis on the process and mechanism of its spread and outbreak after Omicron BA.2 invasion of a city [5, 12]. The recently evolved strains, BA.4 and BA.5, have become dominant in Gauteng and appear to be fuelling a new wave of infections in South Africa, with infectivity about 36% higher than BA.2, which will probably cause the next Omicron wave [13–15].

Studies have proved that invasive species pose one of the most important threats to ecosystems worldwide, often spreading rapidly in new environments and endangering the conservation of native species [16]. And the emergence and transportation of different mutant strains of epidemiology of emergency diseases like COVID-19 into a region can be regarded as a process of biological invasion. In the consideration of the similarity in patterns of spread between invasive pathogens and SARS-CoV-2, the Omicron virus can be considered as a biological invasion, although viral infectious diseases are rarely viewed as this way. Despite a long controversy on how viruses were classified as living organisms, the outbreak process of COVID-19 contains typical features of an invasive species, such as sudden appearance, fast reproduction and spread, adaptation to new environments, large-scale geographic transmission through human transportation networks, and tremendous impacts on human health and society. The epidemic management of SARS-CoV-2 virus needs phase-based processes that are similar with invasion phases of nonpathogenic organisms, which was comparable to five phases: transport, colonization, establishment, landscape spread, outbreak phases, in line with biological invasion theory [17, 18]. Few studies have been performed in the application of biological invasion model to understand the spreading patterns and spillover risks of COVID-19 pandemic with the aim of improving the prevention or control capacity [19]. It is of great importance for further advancement of interdisciplinary methods toward applied research and management of invasive human pathogens and the spillover risk. Thus, we contend that the invasion science [20] is positioned to contribute substantively to understanding the pandemic pattens, including drivers of colonization and establishment, mechanisms of the spread, and factors promoting outbreaks, of novel infectious pathogen of SARS-CoV-2, in particular of Omicron variant with much higher transmissibility compared with other variants.

Mathematical modelling has been essential to inform decision-making processes by investigating the consequences of unmitigated transmission of SARS-CoV-2 in the various invasion phases, as well as the effectiveness of public health social measures. We applied the systematic dynamic model with the public data of four megacities of which population is about or over 10 million, including Suzhou, Nanjing, Shenzhen, Shanghai in The People's Republic of China, where no local case of Omicron variant occurred before the study period but experiencing Omicron variant invasions from January 31 to May 31, 2022. China's dynamic zero-COVID policy is to stem the resurgence of cases in time [21]. The dynamic zero- COVID strategy includes strict public health measures such as strict management of regions with COVID-19 risk, mass nucleic acid testing, contact tracing using the advanced technologies [22]. In order to optimize various combinations of interventions under the dynamic zero-COVID strategy, the investigation was carried out by further understanding invasive patterns and spillover risks of Omicron variant in line with the invasion theory.

## Methods

### Data collection

To parameterize the mathematical model for the interventions on the transmission of COVID-19 (Omicron variant) in The People's Republic of China, we collected the epidemic and intervention data in the megacities from official websites or reports as shown in Additional file 1: Table S1. The megacities were selected by the following criteria: (1) Where no local case of Omicron variant occurred in this city. (2) The interventions strategies were published by local government (like booster vaccination, traffic restriction of metro population-flow in daily records, etc.) were easy to collect. (3) Accessing the spillover risks from one place to other cities. The information of reported cases (symptomatic and asymptomatic) of 4 megacities (Nanjing, Shanghai, Shenzhen and Suzhou) were collected from January 31 to May 31, 2022, provided in public by the National Health Commission (NHC) of China and local Center for Disease Control and Prevention (CDC) as shown in Additional file 1: Table S1. We collected the number of daily symptomatic and asymptomatic confirmed new cases, close contract tracing numbers and their reported locations in each city (Additional file 1).

Response policies by government was taken once the COVID-19 outbreak (after invasion of Omicron variant into the city). Given the difference or description of actual intervention measures taken in each city, with the reference of WHO’s public health and social measures (PHSM) [23] was reported, we standardize the description of measures as following: (1) booster vaccination, (2) mask wearing, (3) traffic restriction, (4) nucleic acid testing, (5) regional management, and (6) contact tracing. The data were collected by four researchers and checked by all of four researchers.

### Data analysis

#### Biological invasion theory framework

Based on the concept of biological invasion [24], this study assumes that the spread of COVID-19 is divided into five phases: transport, colonization, establishment, landscape spread, outbreak (Additional file 1: Fig. S1). The theoretical curves simulated by different initial *R*_*eff*_ were segmented by the time series segmentation (TSS) of piecewise trend approximation (PTA) method, after we classified the biological invasive phases based on theoretical curve, next we determined the segmentation of invasive phases into real outbreak curve. In order to tell the different phases of invasion in real epidemic case, we normalized of a dataset by using the mean value and standard deviation for all scenarios by simulation into the same scale, which comparing data with different cities. Firstly, we calculate the areas under the fitted curve; secondly, we find the areas of each phase according to break point time; thirdly, we find the proportions of each phase areas with areas in the highest point of the ascending period as defined by the derivative; finally, we calculate the area under the curve of real epic curves, with knowing areas in the highest point of the ascending period as defined by the derivative, we use the proportions to detect the phases of invasion in real epics. With real cases developed, there is a possibility that the derivative of the highest point occurred at the beginning with small outbreak of epidemic, we determined this situation as phase of colonization (the time is less than the period for three generations of virus after its invasion). Then, the clustering analysis of study area was carried out by calculating the Moran index for spatial autocorrelation analysis. For the specific calculation process of TSS and the spatial autocorrelation analysis, see Additional file 1.

### Model structure

We considered pre-symptomatic infections based on the basic susceptible-exposed-symptomatic-asymptomatic-recovered/removed (SEIAR) transmission dynamics model according to the previous researches [25–29]. In our model, the whole population were first divided into two groups, completed booster vaccination population and uncompleted booster vaccination population. Furthermore, individuals of each group were divided into six categories: susceptible (S), exposed (E), symptomatic (I_s_), pre-symptomatic (I_p_), asymptomatic (A), removed (R), and quarantine (Q) including recovered and death (Additional file 1: Fig. S2). Also, through transmission dynamics model, we integrated the effects of intervention strategies to SARS-CoV-2 with multiple scenarios by system dynamic model (Additional file 1: Fig. S3), and all parameters were adopted to develop the model, and the description, value, and source are listed in Additional file 1: Table S2. One unique difference of the system dynamic model is that it has been able to simulate independent Omicron variant intervention responses such as the effects of nucleic acid testing and contact tracing, as well as social distancing, isolation, and quarantining. The effective reproductive number (*R*_*eff*_) of the model is as follows, using definition-based method (DBM), *R*_*t*_ is calculated using the EpiEstim package in R. The specific model construction process, parameter estimation, data simulation, calculation formula of *R*_*t*_ and *R*_*eff*_ and intervention measures were shown in Additional file 1, also the intervention simulation corresponding parameters were shown in Additional file 1: Table S4.

### Spatial spillover risk and rank calculation

We use publicly available migration data to calculate the case-count threshold for spillover risk, and if it is higher than the calculated spillover risk, we consider that there is a higher likelihood of spillover risk. Specific assumptions and calculation formulas and values are also presented in the Additional file 1.

### Software for data analysis

All statistical analyses were done in statistical software R (version 4.0., Lucent Technologies, Jasmine Mountain, USA) and Python (version 3.8.9, Software Foundation, Delaware, USA). The RK4 function is used to solve the differential equation of the model in deSolve package (version 1.28) in R, spearman correlation analysis was used to analyze the correlation between *R*_*t*_ and PHSM (the calculation of PHSM-index were shown in Additional file 1: Table S3), and the initial *R*_*eff*_ using the definition-based method is calculated by numpy (version 1.21.4) in Python.

## Results

### Classification of biological invasive phases

Four megacities in The People's Republic of China (Additional file 1: Fig S4) were selected, the invasive Omicron variant existed for 65 days in Shenzhen, 22 days in Nanjing, 21 days in Suzhou, and had been lasting 92 days in Shanghai (as of May 31, 2022), respectively, with their cumulative incidence rates, 26.11‰ (649,418/24,870,000) in Shanghai, being the highest, followed by 0.006‰ (105/17,560,100) in Shenzhen, 0.003‰ (27/9,310,000) in Nanjing and 0.002‰ (22/12,748,262) in Suzhou.

**Fig. 1:**
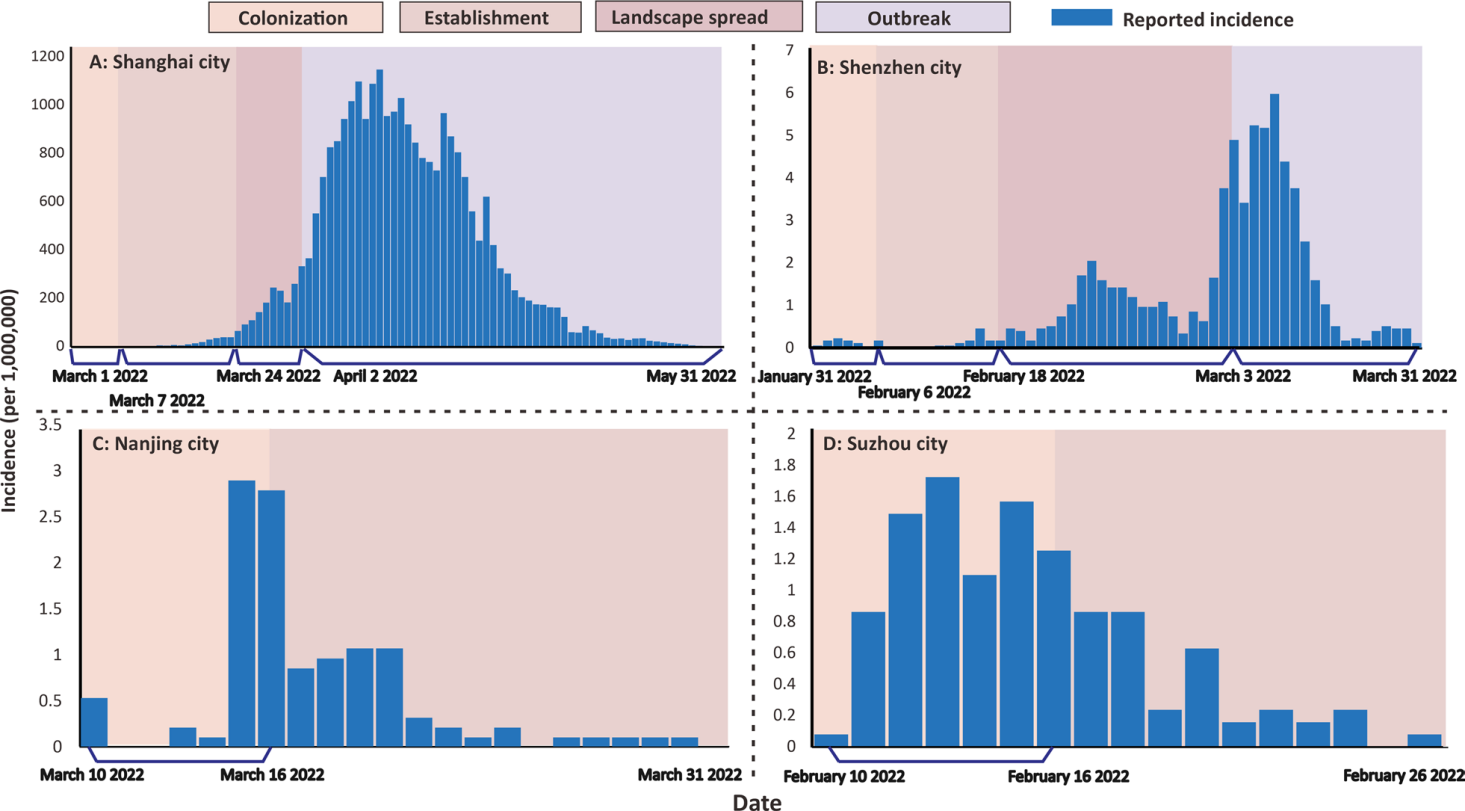
Four phases of the epidemic curve based on the theory of biological invasion (The five phases include transport, colonization, establishment, landscape spread, outbreak. The transport phase is define as the time of reporting of the first case in Additional file 1: Fig. S1. Each phase was partitioned by the segmentation of the theoretical curves. The newly reported cases of each city were illustrated in blue bar. The case distribution patterns in each city is also showed under the figures to corresponding to each epidemic phase)

According to the biological invasion theory, the epidemic from the first invasion day of Omicron variant to the day of the peak of reported cases (refers to the date corresponding to when the peak number of reported incidences reached its peak value) in each city were divided into five phases: transport, colonization, establishment, landscape spread, outbreak, within the epidemic curves plotted by the reported daily incidences in the four cities (Fig. 1).

Differences in phase division were observed in each city both occurred in the theoretical curves (Additional file 1: Fig. S5–S8) and the actual epidemic curve which is influenced by its respective basic vaccinations and interventions. Epidemics in Shanghai, Shenzhen has gone through the whole invasion phases, whereas in Nanjing and Suzhou, the epidemics only ended in the establishment phase. The duration of each phase varied from city to city. As of May 31, Shanghai has already entered the outbreak phase (32 days from transport phase) with the highest incidence rate of 1.058‰, but the peak of epidemic curve has not yet appeared. Shenzhen entered the outbreak phase (31 days from transport phase) and reached the epidemic peak on the 46th day with the highest incidence rate of 0.006‰. The establishment phase lasted 12 days in Shenzhen and 17 days in Shanghai. The duration of the landscape spread in Shanghai and Shenzhen were 9 days and 13 days, respectively. Epidemics in Nanjing and Suzhou have reached the peak of the epidemic at the colonization phase and then showed a decreased trend in the establishment phase, and the highest incidence rate were 0.0028‰, 0.0020‰, respectively.

Spatial autocorrelation of the epidemic varied at each phase in the four cities (Table 1). Shanghai showed a significant spatial aggregation at the very beginning phase of invasion process (Moran's I = 0.106), and spatial aggregation increased from the colonization phase to the outbreak phase. Shenzhen showed spatial dispersion with no statistics significance at the beginning (*P* = 0.602), but showed significant spatial aggregation during the landscape spread phase (Moran's I = 0.239), then this spatial aggregation increased on the outbreak phase (Moran's I = 0.472). Nanjing and Suzhou showed the smaller spatial aggregation with statistics significance only during the colonization phase (Moran's I = 0.037, Moran's I = 0.272).

**Table 1:** Results of invasion phase and spatial autocorrelation analysis

	Phase	Start date	Duration (days)	Number of cumulative reported cases	Moran's I	*P*-value
Shanghai City	Transport	–	–	–	–	–
	Colonization	2022/3/1	6	121	0.106	< 0.01
	Establishment	2022/3/7	17	5852	0.644	< 0.01
	Landscape spread	2022/3/24	9	36,815	0.769	< 0.01
	Outbreak	2022/4/2	> 10	210,796	0.779	< 0.01
Shenzhen City	Transport	–	–	–	–	–
	Colonization	2022/1/31	6	6	− 0.018	0.602
	Establishment	2022/2/6	12	21	− 0.027	0.640
	Landscape spread	2022/2/18	13	179	0.239	< 0.01
	Outbreak	2022/3/3	28	28	0.472	< 0.01
Nanjing City	Transport	–	–	–	–	–
	Colonization	2022/3/10	6	61	0.037	< 0.01
	Establishment	2022/3/16	15	51	–	–
	Landscape spread	–	–	–	–	–
	Outbreak	–	–	–	–	–
Suzhou City	Transport	–	–	–	–	–
	Colonization	2022/2/10	16	31	0.272	< 0.01
	Establishment	2022/2/16	10	124	–	–
	Landscape spread	–	–	–	–	–
	Outbreak	–	–	0	–	–

“–” refers to the null value

### Time-varying reproduction number (*R*_*t*_) value and actual interventions in four cities

**Fig. 2:**
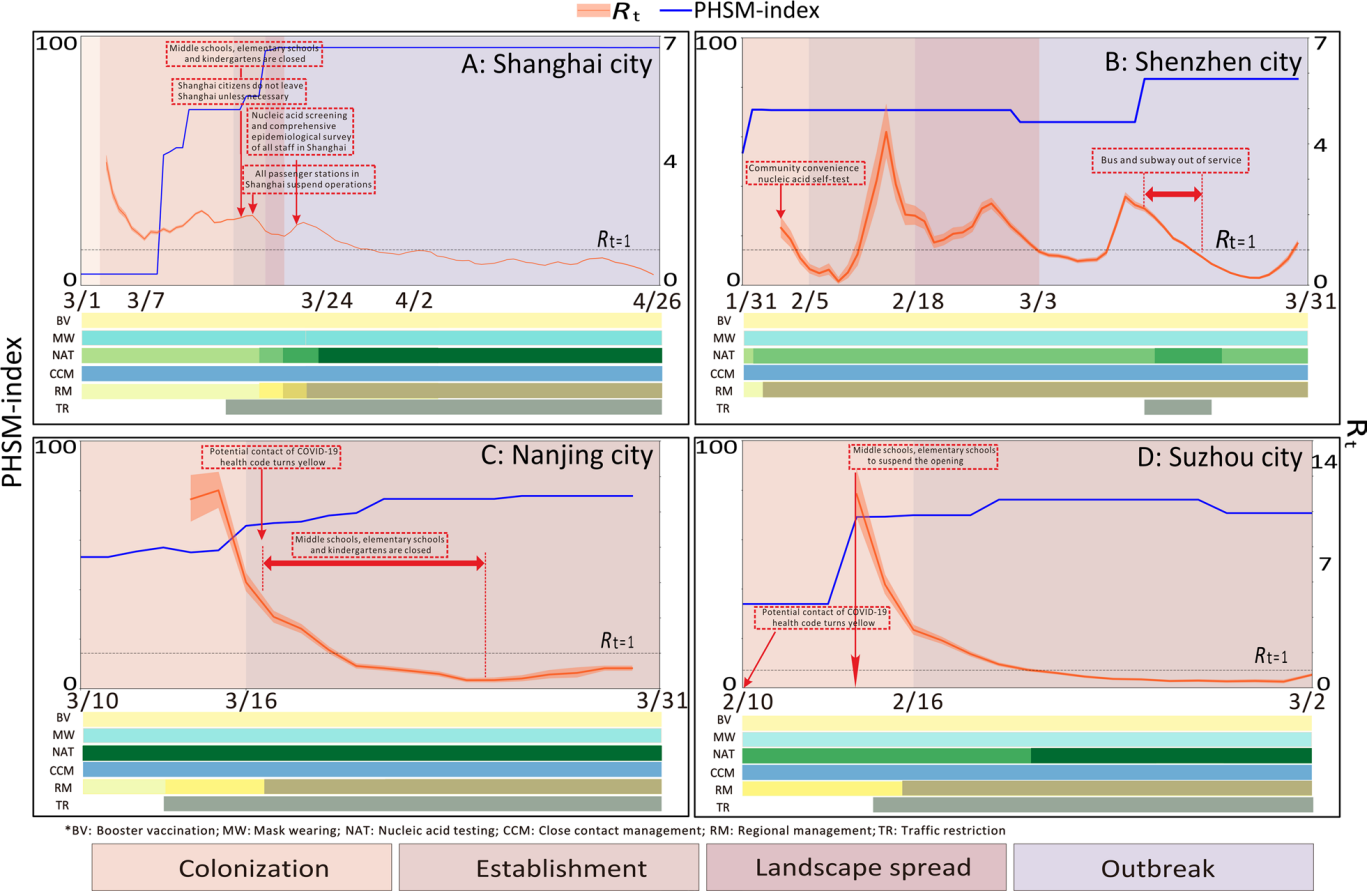
Real-time regeneration numbers and real-time interventions in the studied cities (The PHSM-index of each city was illustrated in light blue lines. The real-time generation number of each city was shown in orange lines with quartile 25 and quartile 75 in orange shades, and the red dashed boxes denote real-time measures. The color of bottom six bars denotes measures of each city, A: Booster vaccination; B: Mask wearing; C: Nucleic acid testing: D: Close contact management; E: Regional management: F: Traffic restriction. The coverage rates of booster vaccination were Shanghai (45.0%), Shenzhen (62.0%), Nanjing (52.7%), and Suzhou (36.4%) before the invasion of Omicron variant. All four cities required the public to wear masks in public places in the early phases of the epidemic (transport phase), and manage the booster vaccination, conducted rules of nucleic acid testing for people at risk and risk area management. Shenzhen, Nanjing and Suzhou started nucleic acid screening in several risk areas at the transport phase. Shanghai has implemented a city-wide management since April 4. From March 28 to 31, all stations of the Shanghai subway were shut down from east and south of the Huangpu River. Shanghai has adopted comprehensive PHSM including citywide “static management”, comprehensive nucleic acid screening, comprehensive mobile surveys, and universal cleaning and disinfection since March 30. Shenzhen has adopted the intervention of "a short, sharp blockade to quell the outbreak" since March 14. Other cities, such as Nanjing and Suzhou, have adopted daily nucleic acid screening)

We build the epidemic curve (Additional file 1: Fig. S5–S8: Five stages of the epidemic curve based on the theory of biological invasion in Shanghai, Shenzhen, Nanjing, and Suzhou, respectively) to understand the nature epidemic patterns of each city based on *R*_*t*_ values at the beginning of Omicron variant invasions. The median (inter-quartile range, IQR) of *R*_*t*_ values (Fig. 2) for the four studied cities, e.g., Shanghai, Shenzhen, Nanjing, Suzhou during the epidemic period are 1.762 (1.757, 1.770), 1.180 (1.067, 1.262), 0.576 (0.514, 0.644), and 0.740 (0.678, 0.807), respectively. The range of PHSM-index values (Fig. 2) for the four studied cities during the epidemic period are 28.88–80.02, 50.36–83.42, 3.20–77.90, and 33.93–76.21, respectively, and more details data were shown in Additional file 1: Table. S6.

During the process of Omicron variant invasion into the four cities, the *Rt* value and PHSM-index of four cities showed a negative correlation in three cities except Shenzhen (*r* = − 0.316, *P* = 0.002; *r* = − 0.255, *P* = 0.060; *r* = − 0.961, *P* = 0.000; *r* = − 0.531, *P* = 0.028) (Additional file 1: Table S7). *R*_*t*_ values in Shenzhen showed significant fluctuations over time, while the other three cities showed a rapid downward trend, with Shanghai showing a small rebound from the initial decline. The specific values for each invasion phase are given in the Additional file 1: Table S5.

The *Rt* value varied among four studied cities that influenced by the value of PHSM-index in the early phases, such as colonization and establishment phases. For example, in Nanjing, *R*_*t*_ value was controlled to below 1 during the phase of establishment with the PHSM-index of 71.0–77.9 and did not rebound due to the rapid public health interventions. Main interventions conducted in Nanjing include nucleic acid screening per 1–2 day in the control and restriction areas. In Suzhou, the *R*_*t*_ value was quickly controlled to under 1 within 7 days at the phase of establishment. In Shenzhen, the *R*_*t*_ value was gradually reduced as the intervention was strengthened at the stage of colonization and early period of establishment with the PHSM-index of 65.97–70.81, but the epidemic rebounded later, when the intra-city transportation was suspended and mass nucleic acid testing was conducted for a week and then the *R*_*t*_ value was effectively controlled to below 1 again when the PHSM-index reaches 83.40. In Shanghai, the *R*_*t*_ values showed a substantially slow trend of reduction with the PHSM-index climbing from 28.88 to 88.02, and has been being *R*_*t*_ > 1 till the end of our analyzing time.

### Simulations of intervention effects

Single intervention and various combinations of interventions were simulated by the system dynamic model with booster vaccination intervention for the four cities. We have simulated 188 various interventions for each city to test whether there were better interventions to achieve the controlling targets at each phase (Additional file 1: Fig. S9–S13: Simulation of different mask wear rates, social distances, isolation ratios, combinations of mask wear and social distance rates, and comprehensive interventions in 4 cities, respectively). And the detailed data of various simulations were founded in Additional file 1: Table S8–S12.

**Fig. 3:**
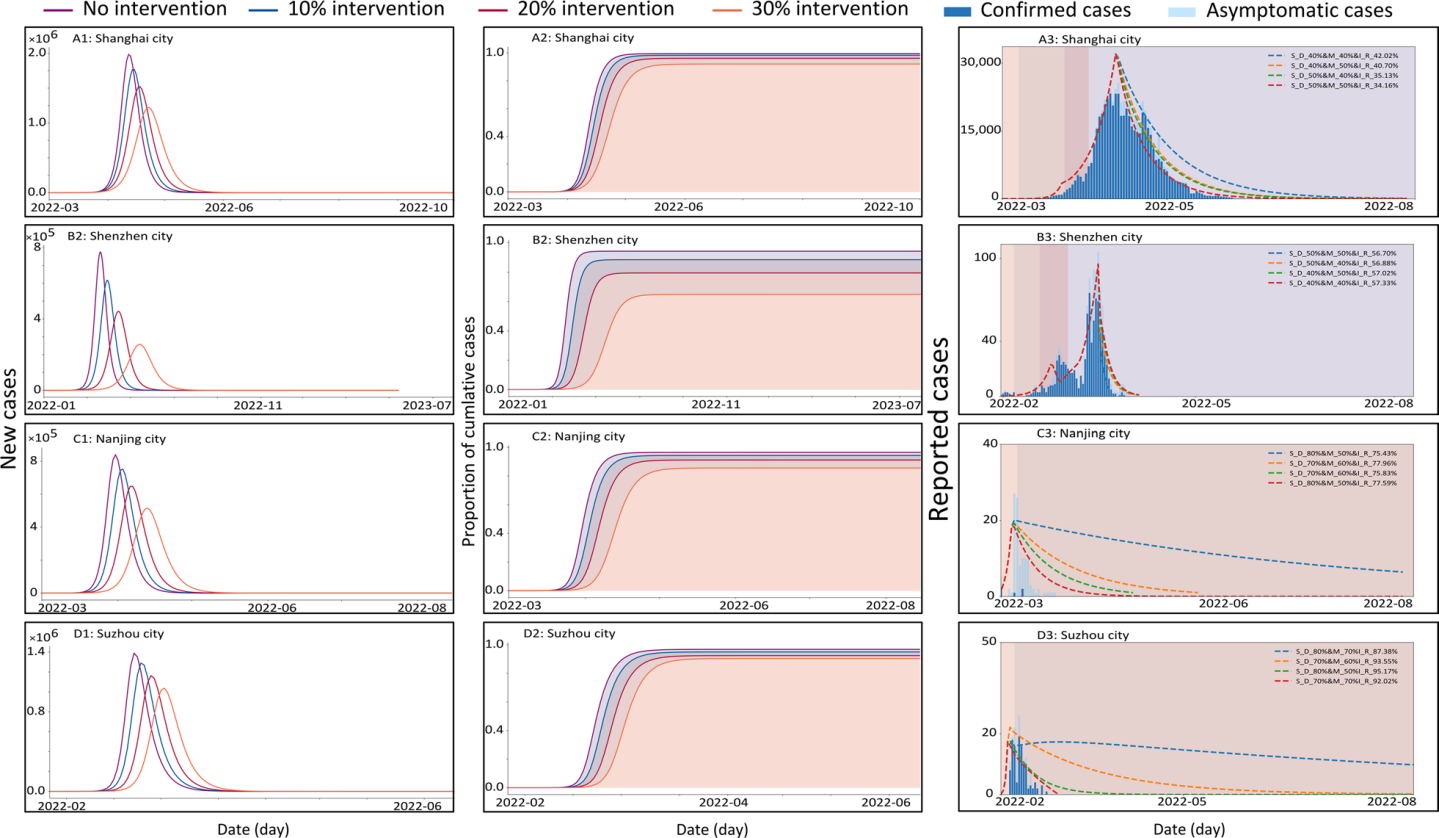
Simulation results of mixed intervention scenarios in the four cities: no intervention, 10–30% intervention. From top to bottom are the four cities, and from left to right are the cumulative number of cases, the number of new cases (four optimal mixed measures applied at 0% (No-intervention), 10% (M_20%&S_D_20%&I_R_10%), 20% (M_20%&S_D_30%&I_R_20%), and 30% strength (M_10%&S_D_50%&I_R_30%), and the number of new cases multiple mixed measures applied at 100% strength, A3**–**D3 denotes the effect of different interventions (specific measures in the legend) in the four cities at the respective intervention dates (Shanghai: Apr 12, Shenzhen: Mar 17, Nanjing: Mar 16, Suzhou: Feb 14)

Firstly, we simulated the trend of the epidemic (cumulative number of cases and new cases) in four cities with no intervention and three different levels of interventions (by reduction of 10%, 20%, 30% for interventions) (Fig. 3A1–D2, A2–D2). The simulation results showed that the cumulative number of cases in all four cities approached 100% in the no-intervention scenario when it reached the outbreak phase, and decreased gradually in the intervention scenario. The cumulative number of cases decreased by 30.37% (IQR: 20.20%, 90.58%) and the time to peak was progressively delayed by 37.5 (IQR: 26, 94.5) days in the four cities as a result of the combined interventions. The results indicated that the intervention has come into effect, to a certain extent, and achieved the goals of "suppressing the peak/cutting the peak and slowing down the epidemic" in various phases of invasion in all studied cities.

Secondly, we simulated the actual situations in four cities (Fig. 3A3–D3), and effects of a combination of four different interventions were analyzed in each city. Since the epidemic in Shanghai was still in an increasing pattern as of April 22, we estimated four combinations of interventions that were realistic for Shanghai. The most effective interventions in the four cities showed in Fig. 3 were red, followed by green, yellow and blue, (M: Mask wear; S_D: Social distance rates; I_R: Isolation rates ). Although both Shanghai and Shenzhen started to intensify their interventions during the outbreak phase, the four different interventions in Shanghai reduced the cumulative incidence by 96.5%, 97.1%, 97.2%, and 97.6%, respectively, compared to the no-intervention state. While the four interventions in Shenzhen reduced the cumulative incidence by almost 100% compared to the no-intervention state. Both Nanjing and Suzhou intensified their interventions at the colonization stage, as a result, their four interventions reduced the cumulative number of cases by 100% relative to the no-intervention state.

The simulations of the real-world epidemic trends are generally consistent as shown in Fig. 3. We have found the highest actual quarantine rate before the landscape spread phase in Suzhou (more than 90%), followed by Nanjing (more than 75%), Shenzhen (more than 55%), and Shanghai (less than 50%), respectively. The comprehensive assessment of intervention intensity of measures in the studied cities demonstrated that the higher intensity of the interventions carried out in the colonization or establishment phases the easier to rapidly control the epidemic and the earlier to achieve the goal of "cutting the peak and slowing down the epidemic" and reducing the medical burden, under the framework of dynamic zero-COVID strategy.

### Assessment of spatial spillover risk

**Fig. 4:**
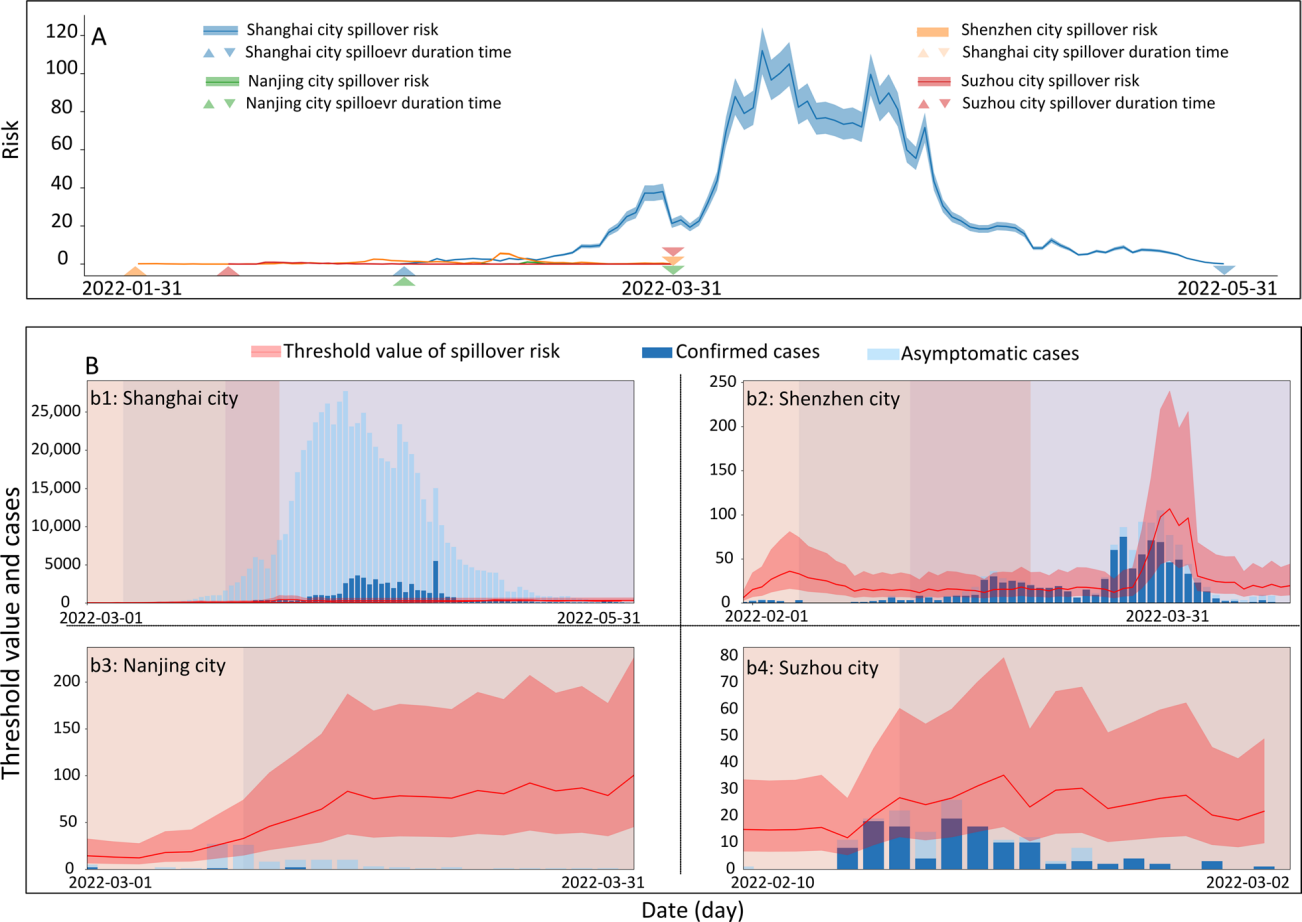
Actual and threshold of spatial spillover risk (**A** The newly reported confirmed cases and asymptomatic cases of each city were illustrated in light blue and dark blue columns. The threshold of spillover risk of each city was shown in pink lines with quartile 25 and quartile 75 in pink shades. **B** The solid line represents the spillover risk, and the positive and inverted triangles represent the duration start and end, respectively. The actual spillover rates of the study regions are distinguished by different colors: Shanghai (blue), Shenzhen (orange), Nanjing (green), and Suzhou (red))

As of May 31, the actual rate of spatial spillover shows that Shenzhen has the longest duration of the outbreak, followed by Shanghai, Suzhou, and Nanjing (Additional file 1: Fig. S14). By simulating the actual number of spillover case in the three cities, except for Shanghai, during the epidemic was less than 1. The median actual spillover cases in Shanghai were 2.858 during the colonization phase and has been in the rising pattern since then (Fig. 4A). The spillover curve is also proportional to the number of reported cases per day, with the risk of spillover increasing as the number of reported cases increased. The actual spillover rate in Shanghai is substantially higher than that of other three cities with the highest spillover risk in all phases, followed by Shenzhen, Nanjing, and Suzhou (Fig. 4A, Additional file 1: Table S13).

The spillover risk threshold is an important indicator reflecting the zero spatial spillover case occurred when the total number of Omicron cases found in one city. From the colonization phase until May 31, most of the spillover risk in Shanghai remained above the spillover risk threshold (IQR) [18.810 (8.478, 42.259)–291.537(144.861, 624.441)] and the actual number of the spillovers (0.947–74.986) was also increasing along the time (Fig. 4B). Shenzhen reported Omicron cases was only above the spillover risk threshold (17.923) at the phase of outbreak, consistent with an actual partial spillover. In Nanjing and Suzhou, the actual number of reported cases did not exceed the spillover alert value (16.127, 14.910) and no actual spillover cases were reported during this round of Omicron epidemic, however, the absence of spillover risks cannot be ruled out.

## Discussion

With the changes in the pandemic patterns of COVID-19, especially the rapid spread of Omicron variant with a large proportion of asymptomatic patients, the effects of control in many countries are not ideal. Although it is in the critical stage to control COVID-19 with faster spreading and higher transmissibility of Omicron variant, China still adheres to the dynamic zero-COVID strategy to prevent the continuous spread and reduce the burden on Chinese population and save more lives in the old, through early detection, rapid containment, and cutting off transmission. Therefore, it is of significance to find the determinants measuring the intensity of Omicron epidemic and giving alarming signals to start different interventions, e.g., the integrated intervention, especially in the raising stage of the disease epidemic [30]. In this study, spatiotemporal invasion dynamics and optimal control strategies of SARS-CoV-2 Omicron variant were investigated. First, the concept of biological invasion was incorporated into the spread of COVID-19 of Omicron variant for the first time, and the segmentation under dynamic zero-COVID strategy was quantitatively analyzed by using TSS and the Moran’s I index. Second, a mathematical model of COVID-19 based on the system dynamics methodology was performed for the evaluation of intervention strategy optimization followed by the index calculation of interventions. Third, it assessed the spatial spillover risks between cities at different invasion phases during the spread of COVID-19 caused by the Omicron variant.

### The dynamic zero-COVID strategy in China

The current dynamic zero-COVID strategy conducted in China, is to take prompt precise and comprehensive approach to contain the sporadic cases once reported in the beginning of invasion phases, cut off the transportation to colonization, and end the epidemic in a timely manner (to “find one, end one”) [31, 32], with an aim to achieve the maximum effectiveness at the lowest cost. Considering the transmission of SARS-CoV-2 Omicron variant happened in a city in the context of biological invasion, the phases from transport to outbreak is determined by the coevolutionary relationship between the organisms and their natural or/and social settings, so do the measures to stop its transmission, particularly the PHSM that we can handle and implement to gain the zero COVID-19 case at each of separate phases [33]. Here we put forward the major interventions needed in each phase. (i) From this study we found that before the transport of imported cases into a city, regular quarantine and surveillance by nuclear acid screening are essential to detect the risk at the earliest time. (ii) At the phases of transport and colonization, the emphasis is mainly on the rapid location of close contacts and strengthened nucleic acid screening among enlarged risk groups. (iii) Identification of close contacts using new technologies like big data analysis is an efficient option before the phase of landscape spread in the golden response time (within 24 h after each report), so as to end the epidemic within one or two maximum incubation periods [21, 34]. (iv) Timely nuclear acid screening is always an essential tool in detecting and controlling the spread of COVID-19 [35]. At the phases of establishment and landscape spread, more intensive measures are implemented among the public, such as the mass screening by nucleic acid screening and end of social activity, so as to cut off possible transmission routes and protect susceptible population from infections. (v) During the outbreak phase, the combination of city-wide enhanced management, daily nucleic acid screening, reduction of severe cases and timely treatment of the COVID-19 patients with other underlying diseases are conducted to limit its spread and control the spatial spillover [33, 34, 36].

The arrival of Omicron variant with the *R*_0_ approximately of 10 and high proportion of asymptomatic cases (93.4%) poses great challenges to the implementation of “dynamic zero-COVID” policy [37], but as long as we adopt the prompter, more precise, more stringent approach, the cessation of sporadic or cluster cases spreading is possible at the early stage of the invasion. As shown in this study, Suzhou halted the epidemic in the establishment phase with the PHSM-index of 69.91–70.77, containing the *R*_t_ of Omicron within 3.28 → 0.74 → 0; Nanjing also stopped the epidemic in the establishment phase, with the PHSM-index of 65.84–77.91 and the *R*_t_ of 3.01 → 0.58 → 0. Although Shenzhen, with the PHSM-index of 70.81 and 65.97 in the establishment phase and outbreak phase, failed to constrain the epidemic from outbreak, but the outbreak phase was ended during a short period (30 days) compared to Shanghai (more than 42 days) where the PHSM-index was 28.88–72.44 and 88.88 in the establishment phase and outbreak phase, respectively.

### Optimal control strategies in 5 invasive phases

Mining the regularity of the data in detail lays the foundation for the precise division of invasion phases and optimization of control strategies in different phases. The results in this study showed that proactive and aggressive containing measures had been implemented by all 4 cities, consequently avoiding approximately 97.8%, 99.9%, 99.9% and 99.9% of the infections, equivalent to 24.20 million, 16.57 million, 8.99 million and 12.34 million people of Shanghai, Shenzhen, Nanjing, Suzhou, respectively, as of May 31, 2022. It was also founded that differences in the spatiotemporal invasion dynamics of the four cities was mainly related to the intensity of control measures in these cities. Nanjing and Suzhou launched a first-level public health emergency response in a very proactive manner, for instance, those response actions including earlier traffic restrictions and nucleic acid screening, more strict social distance, and region management, immediately after the transportation or at the beginning of invasion occurred. These actions have reflected in PHSM-index that increased to over 80 at the colonization phase, far before the outbreak rose to epidemic levels, so that the Omicron variant failed to colonize in Nanjing and Suzhou, reflecting in the pandemic curve that began to be declined during the colonization phase. However, Shenzhen has gradually increased the PHSM efforts during the colonization and establishment phases, and successfully extended the time required for colonization and incubation, leaving more time for the preparation of the medical system. Finally, the pandemic curve reached the peak in the landscape spread phase and then began to decline. In comparison, Shanghai's epidemic gradually increased less intensive PHSM efforts during the periods of colonization, incubation, and landscape spread phases than that in Shenzhen, as a result of that the actual pandemic curve in Shanghai has arrived the outbreak phase. But all those invasive phases were based on the first case import led to city outbreak, frequently invade were not included in our study, and further works will be researched on this part.

Based on the types, implementation time, and intensity of PHSM, the existence of the optimal control strategy is proved at each invasion phase, with following facts. Firstly, compared with single intervention measure, the optimal combination of interventions, including mask wearing, social distance, and quarantine, reduced infection by 99%, which was consistent with previous study [38]. Secondly, in a scenario when implementation of three main interventions is not possible, a strategy of two interventions would be more effective than a single intervention at each invasion phase with the target of controlling the COVID-19 transmission. Thirdly, the event that a testing screen delay of 2 days was less efficient than that implemented two days in advance, when fixed intensity of intervention, has found in the simulation analysis, which demonstrated that earlier interventions are more effective to curb the spread of the epidemic. Finally, the intensity for each intervention measures have been adjusted at the various invasion phases in the invasion process in all cities. We are able to optimize control strategies in Shanghai and Shenzhen based on the different intensive level of combinations of PHSM strategies in each invasion phase, which demonstrated higher intensive measures lead to shorter epidemic period and lower epidemic peak for both cities.

In sensitivity analysis, we varied the basic reproduction number *R*_0_ to assess impact of combinations of interventions. This analysis showed that a combination of all three interventions were effective in nearly all scenarios. Rapid, intensive, and combined strategies can be highly effective in the early control of COVID-19, but places substantial demands on the local public-health authorities. However, upfront expenditures could decrease downstream burden by preventing infected cases, hospitalizations, and additional resource use. This model can provide a valuable reference for policymakers in various cities with different COVID-19 vaccination coverage, enabling them to choose more effective prevention and control strategies based on practical implementation considerations.

### The spillover thresholds in each of invasive phases

It is the first time to apply the concept of spillover effects into the spread of COVID-19 of Omicron variant, in spite of fact that it has been expanded in many fields, such as in stock markets [39], on energy sector [40], and on the US tourism subsectors [41], to represent the exported infection risk to the other locations during the epidemic of Omicron variant with higher transmissibility. The spillover values and spillover thresholds varied in four typical megacities at each phase of Omicron variant invasion. The number of spillover infections, and affected cities nationwide increased gradually from the "transport" phase to the "outbreak" phase of the epidemic base on the analysis for the data from Shanghai and Shenzhen. The model in Shanghai indicated that the median of spillover infections increased from the "transport" phase to "outbreak" phase by 629.1% (Additional file 1: Table S13). This is likely due to the high infectiousness and low pathogenicity of the Omicron variant reflecting in the high proportion of asymptomatic infections that are difficult to detect [42–44], as well as the substantially lower intensity of control measures implemented in Shanghai compared to other three cities. In addition, it can also be seen from the migration index in Shanghai that the overall flow of population at different phases is gradually declining, with the migration index falling by about 94% with 45 days. The low migration index when enter to the outbreak phase in Shanghai remained to the date that we publish this analysis. This shows that although the number of the outgoing population is being controlled gradually, the continuous development of the local outbreak will largely cause the spillover of the disease and thus affect other neighboring cities or areas across the country. The spillover risk in Shenzhen was substantial at the early phase of colonization, when the spillover threshold is exceeded. Even though the outgoing population of Shenzhen spatially spread to many cities in surrounding provinces, the overall spillover was controlled at a low level, with the migration index dropping by around 70% during colonization phase, and has remained at a low level since then. Due to the effective control during the colonization phase of Omicron variant, no substantial spillover risks were identified at any phase in Suzhou and Nanjing. Our analysis indicated that the two important aspects of controlling spillover were local control of epidemic and population outflow control. The data from Suzhou and Nanjing shows that strict measures in the above two dimensions during and before establishment phase can effectively decrease the spillover risk and minimize the risk to other regions. Our findings was supported by another study about spillovers from vaccines and mass drug administration to control infectious disease [45]. It is quite novel to quantify the interregional spillover effects and the extent to which these spillover events have an impact on the increase of COVID-19 outbreak vulnerability in other megacities in China. Our study has also proven that high variation of different invasive phases of Omicron pandemics have distinctive spillover effects in various degrees on neighboring areas if control measures were not strong enough in the early invasion phases.

### Limitations of this study

First, we applied the daily number of reported cases for each city and there may be some time lag between infected and reported dates, which might cause displacement of the invasion phases to some extent. In addition, limited by the testing capacity of nucleic acid screening, the cases from the same population may be reported on different days because of the delay of testing results. However, the daily reported cases could reflect the incidence trends. Second, when analyzing the correlation between PHSM-index and *R*_*t*_, the number of samples of PHSM-index data may have some effects on the results; we did not consider the age grouping for each city in the dynamic models, which might affect the susceptibility of population in the models. Another limitation of this study is that no sensitivity analysis was performed on the parameters of the model, but rather the parameters were set based on previous studies. Third, because of the short time span of the study area and the unclear rate of the attenuation of the protective effect of the vaccine, so we did not consider it when building the model which may have led to an increased risk of patient infection. Fourthly, the absolute number of outflows in this study is estimated by using the Baidu migration index and the absolute number of flows on special holiday, which may have some differences from the real amount of the outflows. The use of Baidu migration index may miss some of the people who go out without smartphones, such as the elderly and children; in addition, in some special cases, when the outflow is controlled, a small number of people may go out without smartphones on purpose to avoid the monitoring of big data. We have therefore set a parameter to the formula for calculating the size of the population migrating from Baidu to correct for the number of people who are really moving, but there is no way to verify whether this parameter is true and valid at the moment.

## Conclusions

We quantified the real epidemic curve for each city into five invasion phases based on the invasion theory to provide evidence for detailed judgments and prompt warnings of epidemic development, which offers Svaluable underexploited frameworks and insights to the COVID-19 pandemic. Also, the system dynamics models simulated optimized interventions and spillover risk in different cities, the importance of early and rapid, high intensity interventions, combined and optimal control measures is the back of the success in cutting off the epidemic peak. It is recommended that after the transport of cases, governments could quickly benchmark the invasion phases proposed in this paper as well as optimal PHSM by simulating interventions, and evaluate the spillover risk at the various phases of invasion, to delay or flatten the epidemic curve inside the city, decreasing the spillover risk outside the city, and finally achieve the goal of dynamic zero case of COVID-19.

## Supplementary Information

**Additional file 1: Table S1.** COVID-19 daily reported cases and response policies by local government. **Table S2.** Variable definitions and parameter values. **Table S3.** PHMS-Index calculation. **Table S4.** Intervention simulation corresponding parameters. **Table S5.** Parameter estimation of actual spillover rate and spillover threshold. **Table S6.** Rt and PHMS-Index values at different stages. **Table S7.** Mean, standard deviation, Person correlation coefficient of Rt and PHSM_Index. **Table S8.** Simulation of different mask wear rates in 4 cities. **Table S9.** Simulation of different social distances in 4 cities. **Table S10.** Simulation of different social distances and mask wear rates in 4 cities. **Table S11.** Simulation of different isolation ratios in 4 cities. **Table S12.** Simulation of different combinations of mask wear, social distance rates and isolation rates in 4 cities. **Table S13.** Spillover risk values at different stages. **Fig. S1**. Biological invasion theory framework. (a: Date of reporting of the first case; b: The time when the third-generation cases appeared, that is, a + 6 days; c, d: According to the PTA methods, the case time series data of points 0-H are calculated in segments.). **Fig. S2.** SEIAR model with booster vaccination intervention. **Fig. S3.** The stock flow diagram of the system dynamic model for COVID-19. (The intervention strategies are represented by causal variables shown in red color. Other causal variables, are shown in parameter tables.). **Fig. S4.** Geographical distribution of the study area and importation of cases into other Chinese cities from study areas (Study areas include Shanghai, Shenzhen, Nanjing, and Suzhou. The red line shows the direction of spillover.). **Fig. S5.** Five stages of the epidemic curve based on the theory of biological invasion in Shanghai (Spread: Landscape spread). **Fig. S6.** Five stages of the epidemic curve based on the theory of biological invasion in Shenzhen (Spread: Landscape spread). **Fig. S7.** Five stages of the epidemic curve based on the theory of biological invasion in Nanjing (Spread: Landscape spread). **Fig. S8.** Five stages of the epidemic curve based on the theory of biological invasion in Suzhou (spread: landscape spread). **Fig. S9.** Simulation of different mask wear rates in 4 cities (a total of 10 mask wear rates were simulated, ranging from 90 to 0%.). **Fig. S10.** Simulation of different social distances in 4 cities (a total of 10 social distances were simulated, ranging from 90 to 0%.). **Fig. S11.** Simulation of different isolation ratios in 4 cities (a total of 26 segregation ratios were modelled, ranging from 80 to 10%.). **Fig. S12.** Simulation of different combinations of mask wear and social distance rates in 4 cities (a total of 56 possible real-world combinations of masks + social distance measures are simulated.). **Fig. S13.** Simulation of comprehensive interventions in 4 cities (a total of 100 possible real- world integrated interventions are simulated.). **Fig. S14.** Map of spillover city top 30 ranking of each city.

## Data Availability

All source code and necessary data are available and accessible at GitHub repository (https://github.com/wjdiadijai/Invasive-dynamic-model).

## Abbreviations

COVID-19: Coronavirus disease 2019
SARS-CoV-2: Severe acute respiratory syndrome coronavirus 2
NHC: National Health Commission
CDC: Center for Disease Control and Prevention
TSS: Time series segmentation
PTA: Piecewise trend approximation
SEIAR: Susceptible-exposed-symptomatic-asymptomatic-recovered/removed
PHSM: Public health and social measures
