# Supplementary material for: Optimal control strategies of SARS-CoV-2 Omicron supported by invasive and dynamic models
Source: Infect Dis Poverty. 2022 Nov 26;11:115. doi: 10.1186/s40249-022-01039-y (PMC9701379; doi:10.1186/s40249-022-01039-y)
Supplement: Supplementary file 1 — Additional file 1: Table S1. COVID-19 daily reported cases and response policies by local government. Table S2. Variable definitions and parameter values. Table S3. PHMS-Index calculation. Table S4. Intervention simulation corresponding parameters. Table S5. Parameter estimation of actual spillover rate and spillover threshold. Table S6. Rt and PHMS-Index values at different stages. Table S7. Mean, standard deviation, Person correlation coefficient of Rt and PHSM_Index. Table S8. Simulation of different mask wear rates in 4 cities. Table S9. Simulation of different social distances in 4 cities. Table S10. Simulation of different social distances and mask wear rates in 4 cities. Table S11. Simulation of different isolation ratios in 4 cities. Table S12. Simulation of different combinations of mask wear, social distance rates and isolation rates in 4 cities. Table S13. Spillover risk values at different stages. Fig. S1. Biological invasion theory framework. (a: Date of reporting of the first case; b: The time when the third-generation cases appeared, that is, a + 6 days; c, d: According to the PTA methods, the case time series data of points 0-H are calculated in segments.). Fig. S2. SEIAR model with booster vaccination intervention. Fig. S3. The stock flow diagram of the system dynamic model for COVID-19. (The intervention strategies are represented by causal variables shown in red color. Other causal variables, are shown in parameter tables.). Fig. S4. Geographical distribution of the study area and importation of cases into other Chinese cities from study areas (Study areas include Shanghai, Shenzhen, Nanjing, and Suzhou. The red line shows the direction of spillover.). Fig. S5. Five stages of the epidemic curve based on the theory of biological invasion in Shanghai (Spread: Landscape spread). Fig. S6. Five stages of the epidemic curve based on the theory of biological invasion in Shenzhen (Spread: Landscape spread). Fig. S7. Five stages of the epidemic curve based [file 40249_2022_1039_MOESM1_ESM.pdf]

Additional Information: Optimal control strategies of SARS-CoV-2 Omicron supported by invasive and dynamic models

Jia Rui[1†], Jin-xin Zheng[2†], Jin Chen[3†], Hongjie Wei[1†], Shanshan Yu[1], Zeyu Zhao[1], Xin-Yi Wang[3], Mu-Xin Chen[3], Shang Xia[3,4], Ying Zhou[4]*, Tianmu Chen[1]*, Xiao-Nong Zhou[3,4]*

Affiliations:

1 State Key Laboratory of Molecular Vaccinology and Molecular Diagnostics, School of Public Health, Xiamen University, Xiamen, People's Republic of China.

2 Department of Nephrology, Ruijin Hospital, Institute of Nephrology, Shanghai Jiao Tong University School of Medicine, Shanghai 200025, People's Republic of China.

3 National Institute of Parasitic Diseases at Chinese Center for Disease Control and Prevention, WHO Collaborating Centre for Tropical Diseases, NHC Key Laboratory of Parasites and Vectors Biology of China, Shanghai 200025, People's Republic of China.

4 School of Global Health, Chinese Center for Tropical Diseases Research, Shanghai Jiao Tong University School of Medicine, Shanghai 200025, People's Republic of China.

CONTENTS

Supplementary Text ... 1
Materials and Methods ... 1
Results Supplement ... 16
References ... 20
Tables Supplement ... 23
Table.S1 COVID-19 daily reported cases and response policies by local government. ... 23
Table.S2 Variable definitions and parameter values. ... 24
Table.S3 PHMS-Index calculation ... 26
Table.S4 Intervention simulation corresponding parameters ... 28
Table.S5 Parameter estimation of actual spillover rate and spillover threshold ... 29
Table.S6 Rt and PHMS-Index values at different stages ... 30
Table.S7 Mean, standard deviation, Person correlation coefficient of Rt and PHSM_Index ... 31
Table.S8 Simulation of different mask wear rates in 4 cities ... 32
Table.S9 Simulation of different social distances in 4 cities ... 34

Table.S10 Simulation of different social distances and mask wearing rate in 4 cities 36
Table.S11 Simulation of different isolation ratios in 4 cities 42
Table.S12 Simulation of different combinations of mask wear, social distance rates and isolation rates in 4 cities 45
Table.S13 Spillover risk values at different stages 55
Fig.S1 Biological Invasion Theory Framework 57
Fig.S2 SEIAR model with booster vaccination intervention 58
Fig.S3 The stock flow diagram of the system dynamic model for Covid-19. 58
Fig.S4 Geographical distribution of the study area and importation of cases into other Chinese cities from study areas. 59
Fig.S5 Five stages of the epidemic curve based on the theory of biological invasion in Shanghai. 60
Fig.S6 Five stages of the epidemic curve based on the theory of biological invasion in Shenzhen. 61
Fig.S7 Five stages of the epidemic curve based on the theory of biological invasion in Nanjing. 62
Fig.S8 Five stages of the epidemic curve based on the theory of biological invasion in Suzhou. 63
Fig.S9 Simulation of different mask wear rates in 4 cities (A total of 10 mask wear rates were simulated, ranging from 90% to 0%.) 64
Fig.S10 Simulation of different social distances in 4 cities (A total of 10 social distances were simulated, ranging from 90% to 0%.) 65
Fig.S11 Simulation of different isolation ratios in 4 cities (A total of 26 segregation ratios were modelled, ranging from 80% to 10%.) 65
Fig.S12 Simulation of different combinations of mask wear and social distance rates in 4 cities (A total of 56 possible real-world combinations of masks + social distance measures are simulated.) 66
Fig.S13 Simulation of comprehensive interventions in 4 cities (A total of 100 possible real-world integrated interventions are simulated.) 67
Fig.S14 Map of Spillover city top30 ranking of each city 67

Supplementary Text

Materials and Methods

Data analysis

Biological Invasion Theory Framework

Based on the concept of biological invasion(*1*), this study assumes that the spread of COVID-19 is divided into five phases: transport, colonization, establishment, landscape spread, outbreak (Fig.S1). The theoretical curves simulated by different initial R_{eff} were segmented by the Time Series Segmentation (TSS) of Piecewise Trend Approximation (PTA) method, and then the actual reported cases were segmented according to the segmentation results of the theoretical curves.

TTS for reported infections

To address the high dimensionality issue in real-world time series data, especially for pandemic trends. We need to model the epidemic time series into one or more segments, which to help us understand the trends in specific periods with the significant information. The PTA is a well-established tool were implemented to split sequence into several segments to find accurate and fast similarity detection in time series(*2*).

Given a time series $\mathrm{Y} = y_1 + y_2 + \cdots + y_t$, where y_t is a real numeric value and t is the timestamp, PTA can be represented as following:

$$\mathrm{T} = \{(X_1, X_{t_1}), \ldots, (X_m, X_{t_m})\}, m \leq t \quad (1)$$

where X_{t_i} is the right end point of the ith segment, X_i ($1 < i \leq m$) is the ratio between $X_{t_i} - 1$ and X_{t_i} in the ith segment, and X_1 is the ratio between the first point t_i and X_{t_1}. The length of the ith segment can be calculated as $X_{t_i} - X_{t_{i-1}}$.

PTA approximates a time series by applying a piecewise discontinuous function to reduce dimensionality. The algorithm of PTA was implemented to our theoretical curves of COVID-19 time series in trending time.

Spatial Autocorrelation Analysis

As the COVID-19 outbreak with the time and space, then case events can have spatial information attached to the map. Therefore, we need a quantitative and objective approach to quantifying the degree to which similar features cluster and where such clustering occurs. The Moran's I statistics were used to examine spatial autocorrelation. This statistic method was first proposed by Moran (1948, 1950) (*3, 4*). The Moran's I statistic is defined as following:

$$I = \frac{\mathrm{u}}{\sum_i \sum_j w_{ij}} \frac{\sum_i \sum_j w_{ij}(z_i - \bar{z})(z_j - \bar{z})}{\sum_i (z_i - \bar{z})^2} \quad (2)$$

where u is number of observations, w_{ij} is the i, j element of a weight matrix W , specifying the degree of dependency between polygons i and j. The implementation here allows only nearest neighbors weighting schemes. Considering the differences between urban subdivisions in the city and small number of case events at the beginning. We builds discrete global grids which partition the surface of the city polygon into hexagonal(*5, 6*), then construct a grid with 5 km spacing between the centers of adjacent hexagons which regard the movement of cases.

The Moran's I statistic ranges from -1 to 1. I<0 indicates negative spatial autocorrelation (Low values tend to have neighbors with high values and vice versa), I=0 indicates no spatial autocorrelation (No spatial pattern - random spatial distribution) and I>0 indicates positive spatial autocorrelation (Spatial clusters of similarly low or high values between neighbor municipalities should be expected.)

Since there is an area size difference in the district division of each city, a global spatial autocorrelation analysis was performed after a 5*5 km grid hexagonal division of the whole city was used to eliminate this bias.

Model structure

We considered pre-symptomatic infections based on the basic Susceptible- Exposed- Symptomatic- Asymptomatic- Recovered/Removed (SEIAR) deterministic model according to the previous research(*7-11*). In our model, the whole population were first divided into two groups, completed booster vaccination population and uncompleted booster vaccination population. Furthermore, individuals of each group were divided into six categories: Susceptible (S), Exposed (E), Symptomatic (I_s), Pre-symptomatic (I_p), Asymptomatic (A), and Removed (R) including recovered and death (Fig.S2). The equations of the model were:

$$\frac{d(S_1)}{dt} = -\beta_{11} * S_1 * (I_{S1} + \kappa * I_{P1} + \kappa * A_1) - \beta_{21} * S_1 * (I_{S2} + \kappa * I_{P2} + \kappa * A_2)$$

$$\frac{d(E_1)}{dt} = \beta_{11} * S_1 * (I_{S1} + \kappa * I_{P1} + \kappa * A_1) + \beta_{21} * S_1 * (I_{S2} + \kappa * I_{P2} + \kappa * A_2) - (1-p) * \omega * E_1 - p * \omega_2 * E_1$$

$$\frac{d(I_{P1})}{dt} = (1-p) * \omega * E_1 - \omega_1 * I_{P1} - h * I_{P1}$$

$$\frac{d(I_{S1})}{dt} = \omega_1 * I_{P1} - \gamma * I_{S1} - h * I_{S1}$$

$$\frac{d(A_1)}{dt} = p * \omega_2 * E_1 - \gamma * A_1 - h * A_1$$

$$\frac{d(R_1)}{dt} = \gamma * I_{S1} + \gamma * A_1$$

$$\frac{d(R_2)}{dt} = \gamma * Q_1$$

$$\frac{d(Q_1)}{dt} = h * (I_{S1} + I_{P1} + A_1) - y * Q_1$$

$$\frac{d(S_2)}{dt} = -\beta_{22} * S_2 * (I_{S2} + \kappa * I_{P2} + \kappa * A_2) - \beta_{12} * S_2 * (I_{S1} + \kappa * I_{P1} + \kappa * A_1)$$

$$\frac{d(E_2)}{dt} = \beta_{22} * S_2 * (I_{S2} + \kappa * I_{P2} + \kappa * A_2) + \beta_{12} * S_2 * (I_{S1} + \kappa * I_{P1} + \kappa * A_1) - (1-p) * \omega * E_2 - p * \omega_2 * E_2$$

$$\frac{d(I_{P2})}{dt} = (1-p) * \omega * E_2 - \omega_1 * I_{P2} - h * I_{P2}$$

$$\frac{d(I_{S2})}{dt} = \omega_1 * I_{P2} - \gamma * I_{S2} - h * I_{S2}$$

$$\frac{d(A_2)}{dt} = p * \omega_2 * E_2 - \gamma * A_2 - h * A_2$$

$$\frac{d(R_3)}{dt} = \gamma * I_{S2} + \gamma * A_2$$

$$\frac{d(R_4)}{dt} = \gamma * Q_2$$

$$\frac{d(Q_2)}{dt} = h * (I_{S2} + I_{P2} + A_2) - y * Q_2$$

$$\mathrm{N} = S_1 + E_1 + I_{S1} + I_{P1} + A_1 + R_1 + R_2 + Q_1 + S_2 + E_2 + I_{S2} + I_{P2} + A_2 + R_3 + R_4 + Q_2$$

(3-19)

This extended SEIAR model follows some basic assumptions, including population is homogeneous and well-mixed interactions without influence by social behavior, age and work. And we add some assumptions to our study:

1) Susceptible population would be infected with a transmission relative rate of β by contact with pre-symptomatic/ symptomatic/ asymptomatic infections, and their transmission relative rate is the same.

2) The incubation period of symptomatic infections was $1/\omega + 1/\omega''$, the latent period of an asymptomatic person was $1/\omega'$.

3) Parameter p ($0 \leq p \leq 1$) gave the proportion of individuals who had asymptomatic infections.

4) Symptomatic infections are communicable in $1/\omega''$ days before developed symptoms.

5) Individuals in categories I_s and A were transferred into category R after an infectious period of $1/\gamma'$ and$1/\gamma$, respectively.

6) Case fatality rate (CFR) was 0 and was not simulated in the model because Omicron variant has low CFR(*12*).

7) We assumed that the infectivity and susceptibility would be reduced after vaccination. VEI and VES(*13*) due to being fully vaccinated were denoted as (1 - x) and (1 - y), respectively(*14-16*).

Parameter estimation approach

Three parameters were estimated based on real data, such as the total population, asymptomatic infection rate and the coverage rate of COVID-19 booster vaccination. In this study, several parameters were adopted to develop the model, and the description, value, and source are listed in Table.S2.

1) According to the statistical year in 2021, the total population of the four cities is 24,870,000, 17,560,100, 9,310,000, 12,748,262, respectively. The number of initial infections (I), including symptomatic and asymptomatic, is obtained from the actual reported data, and the initial values I_0 of the four cities are 2, 1, 5, 1, respectively. The initial values of E and R were set to 0.

2) The coverage rate of COVID-19 booster vaccination in four cities is 45.00%, 62.01%, 52.70%, and 36.35%, respectively.

3) The parameter κ refers to the relative transmissibility rate of asymptomatic to symptomatic individuals. Refer to the previous research, κ is set to 1 in this study (*9, 17*).

4) Since reported asymptomatic patients are far more than infected in Shanghai city, the p in Shanghai is set to 0.8 by assumption, while the p in Shenzhen city, Nanjing city, Suzhou city are set to 0.31 according to the previous research(*18*).

5) As of April 12, 2022, no death case was reported in the report data of the four cities, so this study did not incorporate the case fatality rate (f) in the model.

6) At present, there were few researches on the incubation period of the symptomatic infections(ω) and latent period of the asymptomatic(ω') of Omicron Variant(*19*), so we made assumptions based on the existing literatures of Omicron BA.1, and we assumed that the latent period is the same as the incubation period, it is similar to the previous study in Gauteng and KwaZulu-Natal(*20*). According to the outbreak in Norway(*21*), the median incubation period was 3 days (interquartile range: 3−4); it was 4.2 days (range, 2–8 days) according to other publicly reported data from Korea(*22*); the incubation found by a survey in South Korea median incubation period was 3–4 days(*23*); it was 3 days (interquartile range:1-4 days) in the study of a northern region of Spain(*24*)； We also refer to another study in Japan(*25*), mean incubation periods were 3.7 (95% credible interval (CI) 3.4–4.0) and 5.0 (95% CI 4.5–5.6) days for(*26*) Delta and non-Delta cases, respectively. According to CDC Newsroom report in December 27, 2021(*27*), the $1/\omega''$ was 1-2 days.

7) In this study, the infectious period was set to 4.5 days (γ = γ'=0.22) by our previous research about Delta(*26*) and CDC Newsroom report in December 27, 2021(*27, 28*).

Calculation formula of *R_t and R_{eff}*

The basic reproductive number (R_{eff}) of the model is as follows, using definition-based method (DBM). The interactive R_{ij} between groups in our model, which is defined as the expectation of secondary infections that one infected individual in group i will produce in an entirely susceptible population of group j during its lifespan as infectious, is a quantity of great interest. All R_{ij} forms a matrix that depicts transmission between groups, R_{eff} is defined as the leading eigenvalue of matrix R:

$$R_{eff} = \lambda_{max}(R) \text{ (20)}$$

$$R = \begin{bmatrix} R_{11} & R_{12} \\ R_{21} & R_{22} \end{bmatrix}$$

Step 1: For any infected individual x_i, there are four possible states: E_i, I_{si}, $I_{pi}$and A_i. The first step is to consider the initial state E_i and compute the probability of x_i passing other infectious compartments. For individual $x_i \in E_i$, it might develop into E_i, A_i, and I_{si}, and from I_{si} delvelop into I_{pi}. Since $P(x_i \in E_i) = 1$, the probabilities are computed by ratio of transition rate given in Fig.S2:

$$P(x_i \in E_i) = 1$$

$$P(x_i \in I_{pi}) = P(\in I_{pi} \mid x_i \in E_i)P(x_i \in E_i) = \frac{(1-p)\omega}{p\omega_2 + (1-p)\omega}$$

$$P(x_i \in I_{si}) = P(x_i \in I_{si} \mid x_i \in I_{pi})P(x_i \in I_{pi}) = \frac{(1-p)\omega}{p\omega_2 + (1-p)\omega}$$

$$P(x_i \in A_i) = P(x_i \in A_i \mid x_i \in E_i)P(x_i \in E_i) = \frac{p\omega_2}{p\omega_2 + (1-p)\omega}$$

where $P(x_i \in E_i)$ denotes the probability that individual x_i will develop into the compartment E_i; and $P(\in I_{pi} \mid x_i \in E_i)$ denotes the conditional probability of x_i will develop into

the compartment I_{pi} with $x_i \in E_i$ known.

Step 2: Letting one of four components of $(E_i, I_{pi}, I_{si}, A_i)$ equals to 1, and others equal 0 respectively, and substitute into the term of newly infection rate $\beta_{ij} S_j (I_{si} + \kappa I_{pi} + \kappa A_i)$, one obtains the number of secondary infections in group j, that one infected individual x_i will produce per unit time for the different compartments that x_i might attain:

$$Q_{ij}(x_i \in I_{pi}) = \kappa \beta_{ij} S_j$$

$$Q_{ij}(x_i \in I_{si}) = \beta_{ij} S_j$$

$$Q_{ij}(x_i \in A_i) = \kappa \beta_{ij} S_j$$

Step 3: Take compartment I_{si} as an example, the infectious period of individual x_i is $1/(\gamma + h)$. Similar procedures obtain the infectious period for other compartments of x_i might attain:

$$T(x_i \in I_{pi}) = \frac{1}{\omega_1 + \gamma}$$

$$T(x_i \in I_{si}) = \frac{1}{(\gamma + h)}$$

$$T(x_i \in A_i) = \frac{1}{(\gamma + h)}$$

Step 4: At the beginning of disease transmission, $S \approx N$, and S/N is nearly a constant. As a good 1-order approximation, it is natural to assume that $Q(x_i)$ is constant during the time interval $T(x_i)$.

Step 5: By taking expectations, the expectation of secondary infections in group j that one infected individual in group i will produce during its lifespan as infectious are given by:

$$\begin{aligned} R_{eff,ij} = \ & P(x_i \in I_{pi}) Q_{ij}(x_i \in I_{pi}) T(x_i \in I_{pi}) \\ & + P(x_i \in I_{si}) Q_{ij}(x_i \in I_{si}) T(x_i \in I_{si}) \\ & + P(x_i \in A_i) Q_{ij}(x_i \in A_i) T(x_i \in A_i) \end{aligned}$$

Step 6: Calculating λ_{max} (leading eigenvalue) of matrix R

$$R_{eff,ij} = \lambda_{max}(R) \quad (20)$$

$$R = \begin{bmatrix} R_{11} & R_{12} \\ R_{21} & R_{22} \end{bmatrix}$$

The time-varying reproductive number (R_t) of the model is calculated based on R_0 package. We obtained the intergenerational time of the cases by specifying the transmission relationship of a part of the cases, including epidemiological surveys. The distribution of intergenerational time was estimated by the est.GT function using R_0 package.**PHSM-Index**

We collected the governments' strategies and interventions on COVID-19 across each city from public websites. Policy responses are recorded on ordinal or continuous scales for 5 public health and social measures (PHSM) indicators (Table.S3). The purpose of these indices is to describe the number and degree of intervention, we weight each indicator and each interval on the ordinal scale equally (within each indicator). We calculate the index of public health and social measures (PHMS-Index) in four megacities, including Shanghai, Shenzhen, Nanjing and Suzhou, respectively, with the reference of Oxford COVID-19 Government Response Tracker (OxCGTR) (*29*). The PHMS-Index of policy indicator as follows:

1) Booster vaccination;
2) Mask wearing;
3) Traffic restriction;
4) Nucleic acid testing (sub-indicators);
5) Regional management (sub-indicators).

For the indicators with numerical data, we scale each of these by their maximum value to create a score between 0 and 100; for the indicators with ordinal data, we set the corresponding value as 0 or 1, with a missing value contributing 0; The 1 indicate that this policy was

implemented. The weighted of each indicator was shown in Table.S3. We then averaged these scores to obtain the total PHMS-Index. This calculation is described in equation (21) below, where k is the number of indicators in the index and I_j is the sub-index score for an individual indicator.

$$Index = \frac{1}{k}\sum_{j=1}^{k} Ij \quad (21)$$

In this calculation, we use a conservative assumption that if a datum for one of the component indicators is missing, we consider its contribution to the index is 0.

Intervention measures

Our model is an extension of the classic SEIAR. A mathematical model of Covid-19 based on System Dynamics (SD) methodology is presented (Fig.S3). Our SD based epidemiological model explicitly captures the disease progression pathways, especially for policy interventions pathways, and assumptions for numerous of scenario simulations.

To model the various intervention policies, this is modeled by the flows out of E, I and Q. Quarantine and Isolation restrict the flow in/out from these groups. The specific interventions acting on the model parameters are as follows (Table S4)：

Simulation of booster vaccination

Booster vaccination, wherein the population is divided into two-group wise ‘compartments’ to indicate Covid-19 progression and the interventions. Booster vaccination coverage on the setting of the coefficient X in the model, and the X of each city is set according to the vaccination coverage of the four cities.

Simulation of mask wearing

According to the escape formula $\beta = \frac{1-(1-\psi)^{c}}{N}$, the influencing factors of transmission coefficient (β) can be divided into the probability of single-contact infection (ψ) and the degree of

population contact(c). Assuming that the initial contact degree is c_0=18.8 in Shanghai(*30*), β_0 is fitted by fitting, N is the total population, and ψ_0 is obtained by calculated. Since the population order of the other three cities is similar to Wuhan, it is set to c_0=14.4(*30*). Mask wearing is reflected in the recorded with mask wearing rate in public transportation and the protection efficiency of different types of masks. The mask wearing rate and the protective effect of the mask finally work on the parameter ψ in the model. The simulation reduction rate of ψ was 10%，20%，30%，40%，50%，60%，70%，80%，90% respectively, and the corresponding indicators were calculated to evaluate the simulation effect.

Simulation of traffic restriction

Traffic restriction include transportation shutdown and metro traffic, which is mainly used to control the social distance between people in public places and finally work on the parameter x in the model. The simulation reduction rate of c was 10%，20%，30%，40%，50%，60%，70%，80%，90% respectively, and the corresponding indicators were calculated to evaluate the simulation effect.

Simulation of nucleic acid testing

Nucleic acid testing is divided into five different levels according to the severity of the epidemic in the four cities: a) Nucleic acid testing for close contact, secondary close contact and risk groups, b) Nucleic acid screening in focal area, c) Nucleic acid screening in non-focal regions, d) Nucleic acid screening capabilities, e) Nucleic acid testing in all population. Asymptomatic patients and pre-symptomatic infected patients were easy to be ignored in the early stage, and the transmissibility of the two in the population was as serious as that of symptomatic patients. Therefore, nucleic acid testing could detect and isolate these people as soon as possible, thereby controlling the spread. The proportion of infectious agents found by nucleic acid testing at different

levels corresponded to the isolation coefficient(h) in the model. The actual isolation ratio calculation formula is $\delta = (R_2 + R_4)/(R_1 + R_2 + R_3 + R_4)$. The rate of δ was set to 0-90%, and the corresponding indicators were calculated to evaluate the simulation effect.

Simulation of regional management

Regional management is divided into five different levels according to the severity of the epidemic in the four cities: a) close contact (risk population) management, b) focal area management, c) non-focal area management, d) the management risk area coverage, e) city-wide control and restriction. Regional management can effectively reduce population exposure, so this measure acts on the parameter c in the model.

The indexes for effectiveness of interventions

The indices for effectiveness of interventions are as listed:

1) Total infection rate (TIR): the cumulative number of infections of a disease as a percentage of the total population during a disease outbreak epidemic and is calculated as follows:

   cumulative infection rate = cumulative number of infections/total population × 100%.

2) Epidemic duration: the time interval from the beginning of the epidemic to the end of the epidemic.

3) Peak incidence: the maximum incidence or number of infectious diseases during the disease outbreak pandemic with the smallest calculated time (e.g., day or week).

4) Peak incidence day: the specific time (e.g., date) at which the peak incidence of a disease occurs during a disease outbreak epidemic.

5) Cumulative incidence reduction rate: the reduction in the cumulative number of incidences relative to the initial state through the implementation of different interventions.

Spillover risk and rank calculation

"Spillover event" is the term used to describe when a pathogen overcomes many naturally occurring barriers necessary to "spill over" from one species to another, such as the process of SARS-CoV-2 virus passing from animals to humans has been central to the COVID-19 story since the beginning. Previous study(*31*) have provided evidence of SARS-CoV-2 spillover back and forth between animals and humans. The concept of spillover effects have been expanded to the geographic spillover from one region/territory to another region/territory in many fields, such as in stock markets(*32*) , on energy sector(*33*), and on the US tourism subsectors(*34*).

We calculated the spillover rate of people who infected with SARS-CoV-2 Omicron variant when they migrated from one city to other cities/regions based on the following assumptions.

1) Daily new cases include symptomatic infected, asymptomatic infected and pre-symptomatic cases who fail to isolate in time to be able to go out freely to other cities;

2) Infected individuals are evenly distributed in the society and each patient is as likely to go out as a normal person at the overall level of the society;

3) Patients spilling over to other cities are collectively referred to as spillover cases, all of which are infectious.

4) The migration index can more accurately reflect the daily number of overflow population in each location.

The calculation of the spillover city ranking (RC_s) share is based on CTModelling SeriesEpidemiologist Toolbox (https://toolbox.ctmodelling.cn/app/riskcal). p_{si} denotes the

percentage of Baidu's migrant source/out-migration population at location s on day i of the total population on that day, r_i denotes i days of Baidu migration size index, b denotes upper limit of time i, a denotes number of migrating population origin/destination of migrating population. The calculation process as follows:

$$RC_s = \frac{\sum_{i=1}^{b} p_{si} r_i}{\sum_{s=1}^{a}(\sum_{i=1}^{b} p_{si} r_i)}$$

Actual spillover rate

The formula is as follows, where *C* denotes the number of new infections per day and *N* denotes the total population in each place, f_i, $f_{i,\ upper}$, $f_{i,\ lower}$ denote the number of actual spillover cases and their upper and lower 95% confidence intervals. σ denotes the coefficient of the migration index corresponding to the actual migrating population, which is an assumed value, assuming an initial value of 2. The purpose is to extend the range of the actual spillover rate to twice the original one. *h (t)* denotes the number of migrations per day. *fc* has a practical meaning as the ratio of daily case spillover to total spillover (https://qianxi.baidu.com/#/), and the higher the ratio, the more serious the case spillover is and the need to strengthen prevention and control. Parameter estimation of actual spillover rate and spillover threshold are in Table.S5.

$$f_{i(c,t)} = \frac{\mathrm{C_{(c,t)}}}{\mathrm{N_{(c)}}} * h_{(c,t)} * \mathrm{flow_{out}} \ (23)$$

$$f_{i,lower(c,t)} = \frac{1}{\sigma} * \frac{\mathrm{C_{(c,t)}}}{\mathrm{N_{(c)}}} * h_{(c,t)} * \mathrm{flow_{out,lower}} \ (24)$$

$$f_{i,upper(c,t)} = \frac{1}{\sigma} * \frac{\mathrm{C_{(c,t)}}}{\mathrm{N_{(c)}}} * h_{(c,t)} * \mathrm{flow_{out,upper}} \ (25)$$

Spillover threshold

The formula is as follows, where C_x, $C_{x,\ upper}$, $C_{x,\ lower}$ denote the number of infected persons able to move at the overall daily societal level and their upper and lower 95% confidence intervals. C_x refers to the number of spillover cases, and $C_x \leqslant 1$ means that there is no spillover in theory

$$C_{x(c,t)} = \frac{\mathrm{N_{(c)}}}{h_{(c,t)} * \mathrm{flow_{out}}} \leq 1 \quad (26)$$

$$C_{x,lower(c,t)} = \frac{1}{\sigma} * \frac{\mathrm{N_{(c)}}}{h_{(c,t)} * \mathrm{flow_{out,lower}}} \leq 1 \quad (27)$$

$$C_{x,upper(c,t)} = \sigma * \frac{\mathrm{N_{(c)}}}{h_{(c,t)} * \mathrm{flow_{out,upper}}} \leq 1 \quad (28)$$

Results Supplement

Dynamic patterns of the COVID-19 (Omicron variant) outbreak in the study area

Economically developed megacities (the population is around or over 10 millions) in mainland China have a highly mobile population and frequent interaction with neighboring cities; therefore, in the event of an outbreak, not only the local scale is large, but also the spillover of cases can easily lead to outbreaks in neighboring cities. In this study, four Chinese megacities (Fig.S4) were selected for analysis, including Shanghai, Shenzhen, Nanjing, and Suzhou. As of April 12, the duration of the epidemic was 65 days in Shenzhen city, 43 days in Shanghai city, 22 days in Nanjing city, and 21 days in Suzhou city, from highest to lowest.

Classification of biological invasive phases theoretical curve

We simulated a variety of scenarios to the outbreak of COVID-19 with different R_{eff} (In our study we set R_{eff} = 3, 4, 5, 6, 8, 10, 12) in four cities. A Piecewise Trend Approximation (PTA) method was used in each scenario to detect the time break in our epidemic curves, which were used to determine the phase of establishment, landscape spread and outbreak. In order to tell the different phases of invasion in real epidemic case, we normalized of a dataset by using the mean value and standard deviation for all scenarios by simulation into the same scale, which comparing data with different cities. First, we calculate the areas under the fitted curve; then according to break point time to find the areas of each phase; thirdly, we find the proportions of each phase areas with areas in the highest point of the ascending period as defined by the derivative; finally, we calculate the area under the curve of real epic curves, with knowing the areas in highest point of the ascending period as defined by the derivative, we use the proportions to detect the phases of invasion in real epics. With real cases developed, there is a possibility that the derivative of

highest point occurred at the beginning with small outbreak of epidemic, we determined this situation as phase of colonization (the time is less than the period for three generations of virus after its invasion). Our results of each invasion phase in four cities with different scenarios were shown in Fig.S5-S8.

Correlation analysis of R_t value and PHSM-Index

The study conducted a correlation analysis on the R_t value and PHSM-Index of four cities, and the results showed that there was a detailed negative correlation. The correlation coefficients are shown in Table.S7, the R_t value and PHSM-Index value of Shanghai City (r=-0.504, p=0.001), Nanjing City (r=-0.961, p=0.000), and Suzhou City (r=-0.531, p=0.028) are all negatively correlated. The correlation analysis results in Shenzhen City (r=-0.255, p=0.060) were not statistically significant.

Simulations of intervention effects

In this study, different interventions and combinations of interventions were simulated at different time points in four cities to find the optimal combination of measures, where mask wearing rate, controlled social distance, isolation, mask wearing + social distance and combined measures were simulated for 10, 10, 27, 41 and 100 interventions respectively for a total of 188 interventions. Shanghai, Shenzhen, Nanjing and Suzhou simulated 12, 18, 9 and 9 time point interventions respectively, and only one time point was demonstrated. The effects of the implementation of the different interventions are shown in the Fig.S9-S13 and Table.S8-S12.

We found that in the model simulations it is very difficult to bring an epidemic under control in a short period of time with a single intervention unless it is very strong, but in the real world it

is often difficult to exceed 80% efficiency with a single intervention. In a cross-section of interventions, isolation was the fastest and most effective single intervention to control the epidemic. The time to peak and the cumulative number of cases were better under the dual intervention of masks and controlled social distance than that under a single intervention. The combination of masks, social distance and isolation was the most effective interventions in terms of the number of cases, the time to peak and the number of peak cases occurred.

The effect of the combination measures showed that the duration and peak time of the epidemic tended to stabilize as the intensity of the combination measures increased, and the number of peak cases and cumulative incidence gradually decreased with increasing intensity for the same or similar duration and peak time, and the combination of high-intensity epidemic prevention measures could eliminate the epidemic rapidly in a short period of time, and for many types of measures.

Assessment of spillover risk

This study simulated the top 30 cities in terms of spillover rates due to cases coming from the studied four cities (Fig.S14), among them, the top five risk cities from Shanghai are: Suzhou City (21.87%), Jiaxing City (7.98%), Hangzhou City (4.68%), Nantong City (4.67), and Wuxi City (3.54%); Shenzhen City are: Dongguan City (30.85%), Huzhou City (18.42%), Guangzhou City (8.73%), Foshan City (2.54%), Shanwei City (2.09%); Nanjing City are: Zhenjiang City (14.01%), Chuzhou City (9.61%), Ma'an shan City (8.08%), Changzhou City (4.17%), Yangzhou City (3.94%); Suzhou City are: Shanghai City (42.28%), Wuxi City (14.73%), Nantong City (3.91%), Jiaxing City (3.57%), Huzhou City (2.87%). Notably Shanghai City and Suzhou City are each the cities with the highest risk of spillover from the other.

We conducted a count of the threshold value of spillovers for different periods and the actual number of daily spillovers simulated by the model (Table.S13). The threshold value of spillovers increased with the time in Shanghai (16.825-303.844), Nanjing (14.381-18.582), and Nanjing (14.947-26.611), while threshold value of spillovers in Shenzhen stays around 17 in the latter three periods, with specific figures of 17.502, 15.105, and 17.923, respectively. The median of actual number of daily spillovers simulated by the model in 5 periods, Shanghai increased from 0.119 to 74.986, Shenzhen increased from 0.169 to 0.751, the whole process through the simulation did not appear spillover disease from Suzhou and Nanjing. Actual value did not appear greater than 1, for instance, Nanjing decreased from 0.348 to 0.019 in the first period, and Suzhou increased from 0.067 to 0.147 in the first period.

References

1. C. S. Elton, *The ecology of invasions by animals and plants*. (Springer Nature, 2020).
2. J. P. Dan, W. R. Shi, F. Y. Dong, K. Hirota, Piecewise Trend Approximation: A Ratio-Based Time Series Representation. *Abstr Appl Anal*, (2013).
3. P. A. Moran, Notes on continuous stochastic phenomena. *Biometrika* **37**, 17-23 (1950).
4. P. A. Moran, The interpretation of statistical maps. *Journal of the Royal Statistical Society. Series B (Methodological)* **10**, 243-251 (1948).
5. R. Barnes, K. Sahr, G. Evenden, A. Johnson, F. Warmerdam, dggridR: discrete global grids for R. *R package version 0.1* **12**, (2017).
6. J.-X. Zheng *et al.*, Infestation risk of the intermediate snail host of Schistosoma japonicum in the Yangtze River Basin: improved results by spatial reassessment and a random forest approach. *Infectious diseases of poverty* **10**, 34-46 (2021).
7. Q. Zhao, M. Yang, Y. Wang, L. Yao, T. Chen, Effectiveness of Interventions to Control Transmission of Reemergent Cases of COVID-19 — Jilin Province, China, 2020. *China CDC Weekly* **2**, 651-654 (2020).
8. T. M. Chen *et al.*, A mathematical model for simulating the phase-based transmissibility of a novel coronavirus. *Infect Dis Poverty* **9**, 24 (2020).
9. Z. Y. Zhao *et al.*, A five-compartment model of age-specific transmissibility of SARS-CoV-2. *Infect Dis Poverty* **9**, (2020).
10. S. N. Lin *et al.*, Effectiveness of potential antiviral treatments in COVID-19 transmission control: a modelling study. *Infect Dis Poverty* **10**, 53 (2021).
11. Y. Niu *et al.*, Containing the Transmission of COVID-19: A Modeling Study in 160 Countries. *Front Med (Lausanne)* **8**, 701836 (2021).
12. J. A. Lewnard *et al.*, Clinical outcomes among patients infected with Omicron (B.1.1.529) SARS-CoV-2 variant in southern California. *medRxiv*, 2022.2001.2011.22269045 (2022).
13. Y. Yang *et al.*, The transmissibility and control of pandemic influenza A (H1N1) virus. *Science* **326**, 729-733 (2009).
14. Y. Araf *et al.*, Omicron variant of SARS-CoV-2: Genomics, transmissibility, and responses to current COVID-19 vaccines. *J Med Virol* **94**, 1825-1832 (2022).
15. N. Andrews *et al.*, Covid-19 Vaccine Effectiveness against the Omicron (B.1.1.529) Variant. *N Engl J Med* **386**, 1532-1546 (2022).
16. J. Cai *et al.*, Modeling transmission of SARS-CoV-2 Omicron in China. *Nat Med* **28**, 1468-1475 (2022).
17. S. Hu *et al.*, Infectivity, susceptibility, and risk factors associated with SARS-CoV-2

transmission under intensive contact tracing in Hunan, China. *Nat Commun* **12**, 1533 (2021).
18. H. Nishiura *et al.*, Relative Reproduction Number of SARS-CoV-2 Omicron (B.1.1.529) Compared with Delta Variant in South Africa. *J Clin Med* **11**, (2022).
19. M. Manica *et al.*, Intrinsic generation time of the SARS-CoV-2 Omicron variant: An observational study of household transmission. *Lancet Reg Health Eur* **19**, 100446 (2022).
20. F. Grabowski, M. Kochańczyk, T. Lipniacki, Omicron strain spreads with the doubling time of 3.2-3.6 days in South Africa province of Gauteng that achieved herd immunity to Delta variant. *MedRxiv*, (2021).
21. L. T. Brandal *et al.*, Outbreak caused by the SARS-CoV-2 Omicron variant in Norway, November to December 2021. *Euro Surveill* **26**, (2021).
22. J. J. Lee *et al.*, Importation and Transmission of SARS-CoV-2 B.1.1.529 (Omicron) Variant of Concern in Korea, November 2021. *J Korean Med Sci* **36**, e346 (2021).
23. J. S. Song *et al.*, Serial Intervals and Household Transmission of SARS-CoV-2 Omicron Variant, South Korea, 2021. *Emerg Infect Dis* **28**, 756-759 (2022).
24. J. Del Águila-Mejía *et al.*, Secondary Attack Rates, Transmission, Incubation and Serial Interval Periods of first SARS-CoV-2 Omicron variant cases in a northern region of Spain. (2022).
25. T. Ogata, H. Tanaka, F. Irie, A. Hirayama, Y. Takahashi, Shorter Incubation Period among Unvaccinated Delta Variant Coronavirus Disease 2019 Patients in Japan. *Int J Environ Res Public Health* **19**, (2022).
26. T. Chen *et al.*, Feasibility of COVID-19 control from a pandemic to endemic. (2022).
27. R. P. Walensky, CDC updates and shortens recommended isolation and quarantine period for general population: media statement for immediate release: Monday, December 27, 2021. (2021).
28. W. S. Hart *et al.*, Generation time of the alpha and delta SARS-CoV-2 variants: an epidemiological analysis. *Lancet Infect Dis* **22**, 603-610 (2022).
29. T. Hale *et al.*, A global panel database of pandemic policies (Oxford COVID-19 Government Response Tracker). *Nat Hum Behav* **5**, 529-+ (2021).
30. J. Zhang *et al.*, Changes in contact patterns shape the dynamics of the COVID-19 outbreak in China. *Science* **368**, 1481-1486 (2020).
31. B. B. Oude Munnink *et al.*, Transmission of SARS-CoV-2 on mink farms between humans and mink and back to humans. *Science* **371**, 172-177 (2021).
32. A. Samitas, E. Kampouris, S. Polyzos, Covid-19 pandemic and spillover effects in stock markets: A financial network approach. *International Review of Financial Analysis* **80**, 102005 (2022).

33. D.-K. Si, X.-L. Li, X. Xu, Y. Fang, The risk spillover effect of the COVID-19 pandemic on energy sector: Evidence from China. *Energy Economics* **102**, 105498 (2021).
34. O. Abdelsalam, A. F. Aysan, O. Cepni, M. Disli, The spillover effects of the COVID-19 pandemic: Which subsectors of tourism have been affected more? *Tourism Economics*, 13548166211053670 (2021).

Tables Supplement

Table.S1 COVID-19 daily reported cases and response policies by local government.

Items	Source
**Daily reported cases**	NHC：http://www.nhc.gov.cn
Nanjing	http://wjw.nanjing.gov.cn/
Shanghai	https://wsjkw.sh.gov.cn/
Shenzhen	http://wjw.sz.gov.cn/
Suzhou	http://wsjkw.suzhou.gov.cn/
**Response policies**	Official website or official report
Booster vaccination	Official report
Mask wearing	Regulations for wearing masks in public transportation
Traffic restriction	The announcement on bus, metro or road traffic shutdown by local government
Nucleic acid testing	Official report
Regional management	http://wjw.nanjing.gov.cn/; https://wsjkw.sh.gov.cn/; http://wjw.sz.gov.cn/; http://wsjkw.suzhou.gov.cn/
Contact tracing	http://wjw.nanjing.gov.cn/; https://wsjkw.sh.gov.cn/; http://wjw.sz.gov.cn/; http://wsjkw.suzhou.gov.cn/

*NHC: National Health Commission

Table.S2 Variable definitions and parameter values.

Parameter	Description	Distribution	Range	Source
$1/\omega$	Difference of date between exposed of symptomatic infections	3.66	(1~8)(15-20)	Reference*(18)*
$1/\omega'$	Difference of date between exposed and positive test of asymptomatic infections	3.66	(1~8)(15-20)	Reference*(18)*
$1/\omega''$	The infectious period of pre-symptomatic infections	1	(1~2)	Reference(22)
$1/\gamma'$	Period of category I transferred into category R	4.5	(3~7)	Reference(26- 28)
$1/\gamma$	Period of category A transferred into category R	4.5	(3~7)	Reference(26- 28)
p	Proportion of asymptomatic infections	0.8/0.31	-	Real data/ Reference (16, 29-31)
b	Basic reproductive index	-	-	Fitted
κ	The propagation capacity coefficient of A relative to I	1	-	Reference (9,17)
VES1	Reduction of susceptibility after vaccination for uncompleted booster vaccination population	0.5267	-	Fitted
VEI1	Reduction of infectivity after vaccination for uncompleted booster	0	-	Fitted

	vaccination population			
VES2	Reduction of susceptibility after vaccination for completed booster vaccination population	0.7499	-	Fitted
VEI2	Reduction of infectivity after vaccination for completed booster vaccination population	0.07	-	Assumption

Table.S3 PHMS-Index calculation

PHMS-Index	Sub-indicator	Type	Range	Score	Descriptions
**Vaccination**	Booster vaccination	Numerical	0-100	100	Coverage percentage (Number of booster vaccinations/City population)
**Mask wearing**	Mask wearing	Ordinal	[0,1]	100	Recorded with mask wearing in public transportation
**Traffic restriction**					
	Transportation Shutdown	Ordinal	[0,1]	50	Recorded transportation Shutdown through time in the city
	Metro traffic	Numerical	0-100	50	The Metro Traffic flow (1-The days passenger flow / normal period passenger flow), the value is getting higher, the intervention implemention getting stronger, simultaneously
**Nucleic acid testing**					The intensity of policy measures is from low to high (weight)
	Nucleic acid testing for close contact, secondary close contact and risk groups	Ordinal	[0,1]		Recorded through time in the city
	Nucleic acid screening in focal area	Ordinal	[0,1]	50	Recorded through time in the city
	Nucleic acid screening in non-focal regions	Ordinal	[0,1]		Recorded through time in the city
	Nucleic acid screening capabilities	Numerical	0-100	50	Record the number of nucleic acid screening through time period
	Nucleic acid testing in all population	Ordinal	[0,1]	100	If a full population nucleic acid test is taken, means the stronger policy implemented, above four items have been covered.
**Regional management**					The intensity of control measures is from low to high (weight)
	Close contact (risk population) management	Ordinal	[0,1]	50	Recorded through time in the city

Focal area management	Ordinal	[0,1]		Recorded through time in the city
Non-focal area management	Ordinal	[0,1]		Recorded through time in the city
The management risk area coverage	Numerical	0-100	50	Recorded the management risk area through time in the city
City-wide control and restriction	Ordinal	[0,1]	100	If city-wide control and restriction is taken, means the stronger policy implemented, all regional management have been covered.

Table.S4 Intervention simulation corresponding parameters

Parameter	Description	PHMS-Index	Range	Source/Method
ψ	The probability of single-contact infection	Mask wearing	0–90%	Assumption
c	The degree of population contact	Traffic restriction; Regional management	0–90%	Assumption
δ	The actual isolation ratio	Nucleic acid testing	0–90%	Fitted

Table.S5 Parameter estimation of actual spillover rate and spillover threshold

Parameter	Description	Distribution	Source/Method
$N_{shanghai}$	Total population of Shanghai	24,870,000	Real data
$N_{shenzhen}$	Total population of Shenzhen	17,560,100	Real data
$N_{nanjing}$	Total population of Nanjing	9,310,000	Real data
N_{suzhou}	Total population of Suzhou	12,748,262	Real data
$flow_{out}$	Migrant population corresponding to a unit migration index	210,420	Fitted
$flow_{out,upper}$	The upper limit of the migrating population corresponding to a unit migration index	233,394	Fitted
$flow_{out,lower}$	The lower limit of the migrating population corresponding to a unit migration index	187,254	Fitted
$h_{(t)}$	Migration Index	-	Real data
σ	Coefficient of the migration index corresponding to the actual migration population	2	Assumption

Table.S6 Rt and PHMS-Index values at different stages.

Region		Transport	Colonization	Establishment	Landscape spread	Outbreak
Shanghai City	Date	2022/3/1	2022/3/1–2022/3/6	2022/3/7–2022/3/23	2022/3/24–2022/4/1	2022/4/2–2022/4/12
	Rt	null	3.366 (3.176–3.563)	1.859 (1.842–1.876)	1.926 (1.91–1.942)	1.549 (1.545–1.553)
	PHSM_index	28.88	28.88	60.433	76.01	88.021
Shenzhen City	Date	2022/1/31	2022/1/31–2022/2/5	2022/2/6–2022/2/17	2022/2/18–2022/3/2	2022/3/3–2022/3/31
	Rt	null	2.178 (1.731–2.337)	1.653 (1.115–1.86)	1.924 (1.776–2.002)	0.971 (0.86–1.008)
	PHSM_index	53.69833333	70.808	70.808	70.808	83.402
Nanjing City	Date	2022/3/10	2022/3/10–2022/3/15	2022/3/16 ～ 2022/3/31	null	null
	Rt	null	5.487 (4.93–6.085)	0.576 (0.514–0.644)	null	null
	PHSM_index	53.20751938	55.489	76.707	null	null
Suzhou City	Date	2022/2/10	2022/2/10–2022/2/15	2022/2/16 ～ 2022/3/2	null	null
	Rt	null	8.418 (7.513–9.393)	0.638 (0.58–0.7)	null	null
	PHSM_index	33.95066667	69.255	76.21	null	null

Table.S7 Mean, standard deviation, Person correlation coefficient of Rt and PHSM_Index

Region	Variable	Mean	SD	r	*p*
Shanghai city	*Rt*	1.809	0.479	-0.606^{**}	0.001
	PHSM_Index	66.545	22.937		
Shenzhen city	*Rt*	1.270	0.834	-0.255	0.060
	PHSM_Index	73.556	6.920		
Nanjing city	*Rt*	1.393	1.711	$-.961^{**}$	0
	PHSM_Index	72.076	7.582		
Suzhou city	*Rt*	1.892	2.754	$-.531^{*}$	0.028
	PHSM_Index	72.998	3.154		

**p<0.01, *p<0.05

Table.S8 Simulation of different mask wear rates in 4 cities

	Measure	Max cases	Get max cases time	Duration time	All cases	Decline rate
Shanghai City	No Intervention	1836285	49	116	24333150	0.00%
	M_10%	1792105	50	118	24289633	0.18%
	M_20%	1670020	52	123	24152316	0.74%
	M_30%	1649638	52	124	24130066	0.83%
	M_40%	1547011	54	129	23976230	1.47%
	M_50%	1409418	57	136	23714814	2.54%
	M_60%	1218958	62	148	23225485	4.55%
	M_70%	946776	70	171	22163315	8.92%
	M_80%	548284	92	231	19237530	20.94%
	M_90%	28985	297	885	5774763	76.27%
Shenzhen City	No Intervention	630390	87	182	15236301	0.00%
	M_10%	599308	90	187	15066526	1.11%
	M_20%	520921	96	203	14563888	4.41%
	M_30%	510324	98	206	14486138	4.92%
	M_40%	446062	105	222	13969305	8.32%
	M_50%	363559	116	249	13151421	13.68%
	M_60%	257607	137	299	11745089	22.91%
	M_70%	126764	191	426	8988881	41.00%
	M_80%	6240	632	993	2250322	85.23%
	M_90%	36	27	73	671	100.00%
Nanjing City	No Intervention	839297	31	87	8993194	0.00%
	M_10%	818016	31	88	8989747	0.04%
	M_20%	784403	33	91	8977993	0.17%
	M_30%	776515	33	92	8975974	0.19%
	M_40%	743384	35	95	8961263	0.36%
	M_50%	697228	37	99	8933744	0.66%
	M_60%	629027	40	107	8876070	1.30%
	M_70%	532462	46	120	8733901	2.88%
	M_80%	381266	60	150	8285793	7.87%
	M_90%	118108	123	296	5915348	34.22%
Suzhou City	No Intervention	1770814	15	24	14275397	0.00%
	M_10%	1738685	16	63	13125432	8.06%
	M_20%	1750389	16	69	12485175	12.54%
	M_30%	1744134	16	69	12458097	12.73%

M_40%	1689982	17	70	12385867	13.24%
M_50%	1668702	17	71	12363409	13.39%
M_60%	1611750	18	72	12353706	13.46%
M_70%	1528749	20	73	12346451	13.51%
M_80%	1370148	23	77	12337391	13.58%
M_90%	1018253	32	94	12260895	14.11%

* M: mask wearing rate

Table.S9 Simulation of different social distances in 4 cities

	Measure	Max cases	Get max cases time	Duration time	All cases	Decline rate
Shanghai City	No Intervention	1836285	49	116	24333150	0.000%
	S_D_10%	1794726	50	118	24292371	0.168%
	S_D_20%	1736935	51	120	24232876	0.412%
	S_D_30%	1661529	52	124	24142338	0.784%
	S_D_40%	1563903	54	128	24000672	1.366%
	S_D_50%	1429570	57	135	23761650	2.349%
	S_D_60%	1249183	61	146	23313451	4.191%
	S_D_70%	986176	69	167	22343874	8.175%
	S_D_80%	591803	89	222	19674262	19.146%
	S_D_90%	47225	251	723	7238370	100.000%
Shenzhen City	No Intervention	630390	87	182	15236301	0.000%
	S_D_10%	601021	89	187	15077031	1.045%
	S_D_20%	563996	93	194	14853445	2.513%
	S_D_30%	516710	97	204	14528910	4.643%
	S_D_40%	455151	104	219	14049297	7.791%
	S_D_50%	376570	114	244	13293595	12.751%
	S_D_60%	273411	134	289	11989103	21.312%
	S_D_70%	143250	180	401	9438085	38.055%
	S_D_80%	12881	485	993	3241303	78.726%
	S_D_90%	36	27	77	709	99.995%
Nanjing City	No Intervention	839297	31	87	8993194	0.000%
	S_D_10%	819873	31	88	8989968	0.036%
	S_D_20%	806024	32	89	8985042	0.091%
	S_D_30%	781178	33	91	8977091	0.179%
	S_D_40%	745446	35	94	8963682	0.328%
	S_D_50%	702311	37	98	8938874	0.604%
	S_D_60%	641533	40	105	8886913	1.182%
	S_D_70%	546454	46	118	8759251	2.601%
	S_D_80%	398633	58	146	8355889	7.087%
	S_D_90%	133965	115	277	6184942	31.226%
Suzhou City	No Intervention	1770814	15	24	14275397	0.000%
	S_D_10%	1737449	16	63	13155394	7.846%
	S_D_20%	1754865	16	68	12703086	11.014%
	S_D_30%	1747833	16	69	12471997	12.633%

S_D_40%	1684480	17	70	12391024	13.200%
S_D_50%	1682274	17	70	12365383	13.380%
S_D_60%	1629143	18	71	12354770	13.454%
S_D_70%	1529686	20	73	12347307	13.506%
S_D_80%	1381354	23	77	12338571	13.568%
S_D_90%	1042143	31	92	12273701	14.022%

*S_D: social distance

Table.S10 Simulation of different social distances and mask wearing rate in 4 cities

	Measure	Max cases	Get max cases time	Duration time	All cases	Decline rate
Shanghai City	No Intervention	1836285	49	116	24333150	0.000%
	S_D_10%&M_10%	1794726	50	118	24292371	0.168%
	S_D_10%&M_20%	1739252	51	120	24236101	0.399%
	S_D_20%&M_10%	1736935	51	120	24232876	0.412%
	S_D_20%&M_20%	1675891	52	123	24160141	0.711%
	S_D_30%&M_10%	1661529	52	124	24142338	0.784%
	S_D_10%&M_30%	1604111	53	126	24063500	1.108%
	S_D_30%&M_20%	1586681	53	127	24044170	1.188%
	S_D_10%&M_40%	1579782	54	127	24034238	1.228%
	S_D_40%&M_10%	1563903	54	128	24000672	1.366%
	S_D_20%&M_30%	1522955	55	130	23936120	1.632%
	S_D_20%&M_40%	1503507	55	131	23899589	1.782%
	S_D_40%&M_20%	1479673	56	132	23864240	1.927%
	S_D_10%&M_50%	1471239	56	133	23840765	2.024%
	S_D_30%&M_30%	1424025	57	135	23746341	2.412%
	S_D_30%&M_40%	1400347	57	137	23698583	2.608%
	S_D_20%&M_50%	1380500	58	138	23652265	2.798%
	S_D_50%&M_10%	1340833	59	140	23561289	3.172%
	S_D_40%&M_30%	1299230	60	143	23455374	3.607%
	S_D_40%&M_40%	1274654	60	144	23389543	3.878%
	S_D_30%&M_50%	1266538	60	145	23372439	3.948%
	S_D_60%&M_10%	1148770	64	153	22997853	5.488%
	S_D_50%&M_20%	1140337	64	154	22966508	5.616%
	S_D_40%&M_50%	1132887	64	155	22942541	5.715%
	S_D_50%&M_30%	1112830	65	156	22868195	6.020%
	S_D_50%&M_40%	963605	70	170	22237505	8.612%
	S_D_60%&M_20%	930309	71	173	22073262	9.287%
	S_D_60%&M_30%	901325	72	176	21923669	9.902%
	S_D_70%&M_10%	877685	73	179	21793405	10.437%
	S_D_50%&M_50%	779764	78	191	21190201	12.916%
	S_D_60%&M_40%	745137	80	196	20955086	13.883%
	S_D_70%&M_20%	649410	85	211	20198420	16.992%
	S_D_70%&M_30%	622284	87	216	19959250	17.975%
	S_D_60%&M_50%	563992	91	227	19398871	20.278%
	S_D_80%&M_10%	485080	98	246	18525490	23.867%
	S_D_70%&M_40%	469096	99	250	18329059	24.675%
	S_D_60%&M_60%	352037	113	290	16645064	31.595%

	S_D_70%&M_50%	299402	121	314	15709025	35.442%
	S_D_80%&M_20%	276486	126	326	15254312	37.311%
	S_D_80%&M_30%	253626	130	340	14768619	39.307%
	S_D_80%&M_40%	134282	169	459	11453948	52.929%
	S_D_80%&M_50%	36692	274	804	6445211	73.513%
	No Intervention	630390	87	182	15236301	0.000%
	S_D_10%&M_10%	601021	89	187	15077031	1.045%
	S_D_10%&M_20%	566151	92	194	14865332	2.435%
	S_D_20%&M_10%	563996	93	194	14853445	2.513%
	S_D_20%&M_20%	525453	96	202	14591443	4.232%
	S_D_30%&M_10%	516710	97	204	14528910	4.643%
	S_D_10%&M_30%	480729	101	213	14258394	6.418%
	S_D_30%&M_20%	472479	102	215	14193500	6.844%
	S_D_10%&M_40%	468833	102	216	14160355	7.062%
	S_D_40%&M_10%	455151	104	219	14049297	7.791%
	S_D_20%&M_30%	431466	106	226	13839538	9.167%
	S_D_20%&M_40%	419210	108	230	13722858	9.933%
	S_D_40%&M_20%	407363	110	233	13611219	10.666%
	S_D_10%&M_50%	399969	111	236	13537734	11.148%
	S_D_30%&M_30%	372165	115	245	13246940	13.057%
	S_D_30%&M_40%	359167	117	250	13102525	14.005%
	S_D_20%&M_50%	347136	119	255	12964014	14.914%
Shenzhen City	S_D_50%&M_10%	325042	123	264	12696020	16.673%
	S_D_40%&M_30%	301628	127	275	12390096	18.680%
	S_D_40%&M_40%	288200	130	282	12202910	19.909%
	S_D_30%&M_50%	284836	131	283	12154614	20.226%
	S_D_60%&M_10%	221240	148	323	11127444	26.968%
	S_D_50%&M_20%	216697	149	326	11043794	27.517%
	S_D_40%&M_50%	213260	150	329	10980045	27.935%
	S_D_50%&M_30%	203026	154	337	10783418	29.225%
	S_D_50%&M_40%	133326	186	415	9172690	39.797%
	S_D_60%&M_20%	119174	196	439	8767130	42.459%
	S_D_60%&M_30%	107386	205	461	8401873	44.856%
	S_D_70%&M_10%	97909	214	481	8086782	46.924%
	S_D_50%&M_50%	62095	260	594	6659105	56.294%
	S_D_60%&M_40%	51139	282	648	6114783	59.867%
	S_D_70%&M_20%	24740	377	889	4400810	71.116%
	S_D_70%&M_30%	18757	420	993	3869509	74.603%
	S_D_60%&M_50%	8380	569	993	2630258	82.737%
	S_D_80%&M_10%	550	1001	993	205578	98.651%
	S_D_70%&M_40%	117	1001	993	67432	99.557%

	S_D_60%&M_60%	37	27	293	2644	99.983%
	S_D_70%&M_50%	37	27	198	1794	99.988%
	S_D_80%&M_20%	37	27	174	1578	99.990%
	S_D_80%&M_30%	36	27	156	1411	99.991%
	S_D_80%&M_40%	36	27	100	912	99.994%
	S_D_80%&M_50%	36	27	75	687	99.995%
Nanjing City	No Intervention	839297	31	87	8993194	0.000%
	S_D_10%&M_10%	819873	31	88	8989968	0.036%
	S_D_10%&M_20%	807291	32	89	8985316	0.088%
	S_D_20%&M_10%	806024	32	89	8985042	0.091%
	S_D_20%&M_20%	786556	33	91	8978696	0.161%
	S_D_30%&M_10%	781178	33	91	8977091	0.179%
	S_D_10%&M_30%	763010	34	93	8969763	0.261%
	S_D_30%&M_20%	758021	34	93	8967914	0.281%
	S_D_10%&M_40%	754885	34	94	8966957	0.292%
	S_D_40%&M_10%	745446	35	94	8963682	0.328%
	S_D_20%&M_30%	735596	35	95	8957229	0.400%
	S_D_20%&M_40%	724284	35	96	8953493	0.441%
	S_D_40%&M_20%	721727	36	97	8949821	0.482%
	S_D_10%&M_50%	718393	36	97	8947353	0.510%
	S_D_30%&M_30%	701170	37	99	8937207	0.623%
	S_D_30%&M_40%	694405	37	100	8931948	0.681%
	S_D_20%&M_50%	683644	37	100	8926770	0.739%
	S_D_50%&M_10%	673868	38	102	8916397	0.854%
	S_D_40%&M_30%	659566	39	103	8904002	0.992%
	S_D_40%&M_40%	649496	39	104	8896141	1.079%
	S_D_30%&M_50%	646003	39	105	8894080	1.102%
	S_D_60%&M_10%	606483	42	110	8847219	1.623%
	S_D_50%&M_20%	603574	42	110	8843164	1.668%
	S_D_40%&M_50%	600822	42	110	8840051	1.703%
	S_D_50%&M_30%	592250	43	111	8830329	1.811%
	S_D_50%&M_40%	539969	46	119	8744364	2.767%
	S_D_60%&M_20%	527919	47	121	8721114	3.025%
	S_D_60%&M_30%	516838	48	123	8699676	3.264%
	S_D_70%&M_10%	507592	48	124	8680818	3.473%
	S_D_50%&M_50%	471257	51	131	8591484	4.467%
	S_D_60%&M_40%	457990	52	133	8555878	4.863%
	S_D_70%&M_20%	421389	56	141	8438834	6.164%
	S_D_70%&M_30%	410787	57	143	8401157	6.583%
	S_D_60%&M_50%	387157	59	149	8311786	7.577%
	S_D_80%&M_10%	355332	63	157	8169846	9.155%

S_D_70%&M_40%	348667	64	159	8137534	9.515%
S_D_60%&M_60%	298274	71	175	7855675	12.649%
S_D_70%&M_50%	274477	75	185	7695844	14.426%
S_D_80%&M_20%	263780	77	189	7617512	15.297%
S_D_80%&M_30%	252930	79	194	7533392	16.232%
S_D_80%&M_40%	191036	94	228	6948809	22.733%
S_D_80%&M_50%	125191	119	287	6039116	32.848%

	No Intervention	1770814	15	24	14275397	0.000%
	S_D_10%&M_10%	1737449	16	63	13155394	7.846%
	S_D_10%&M_20%	1754402	16	68	12718531	10.906%
	S_D_20%&M_10%	1754865	16	68	12703086	11.014%
	S_D_20%&M_20%	1752102	16	69	12496885	12.459%
	S_D_30%&M_10%	1747833	16	69	12471997	12.633%
	S_D_10%&M_30%	1714237	16	70	12411653	13.056%
	S_D_30%&M_20%	1702753	16	70	12403801	13.111%
	S_D_10%&M_40%	1696415	16	70	12400366	13.135%
	S_D_40%&M_10%	1684480	17	70	12391024	13.200%
	S_D_20%&M_30%	1696016	17	70	12379424	13.281%
	S_D_20%&M_40%	1698371	17	70	12375062	13.312%
	S_D_40%&M_20%	1697902	17	70	12371774	13.335%
	S_D_10%&M_50%	1696150	17	70	12369967	13.348%
	S_D_30%&M_30%	1678252	17	71	12364691	13.385%
	S_D_30%&M_40%	1663147	17	71	12362813	13.398%
	S_D_20%&M_50%	1645058	17	71	12361306	13.408%
	S_D_50%&M_10%	1635107	18	71	12358960	13.425%
	S_D_40%&M_30%	1640878	18	71	12356886	13.439%
Suzhou City	S_D_40%&M_40%	1638085	18	71	12355831	13.447%
	S_D_30%&M_50%	1636595	18	71	12355579	13.448%
	S_D_60%&M_10%	1582934	19	72	12351491	13.477%
	S_D_50%&M_20%	1584219	19	72	12351231	13.479%
	S_D_40%&M_50%	1584758	19	72	12351037	13.480%
	S_D_50%&M_30%	1584029	19	72	12350466	13.484%
	S_D_50%&M_40%	1530747	20	73	12346793	13.510%
	S_D_60%&M_20%	1523415	20	73	12346054	13.515%
	S_D_60%&M_30%	1507893	20	74	12345433	13.520%
	S_D_70%&M_10%	1488144	20	74	12344924	13.523%
	S_D_50%&M_50%	1466203	21	75	12342850	13.538%
	S_D_60%&M_40%	1440808	21	75	12342129	13.543%
	S_D_70%&M_20%	1412335	22	76	12339985	13.558%
	S_D_70%&M_30%	1389408	22	76	12339337	13.562%
	S_D_60%&M_50%	1377220	23	77	12337829	13.573%
	S_D_80%&M_10%	1335531	24	78	12335413	13.590%
	S_D_70%&M_40%	1332734	24	78	12334847	13.594%
	S_D_60%&M_60%	1270587	25	80	12329462	13.631%
	S_D_70%&M_50%	1244408	26	82	12325959	13.656%
	S_D_80%&M_20%	1227114	26	82	12324105	13.669%
	S_D_80%&M_30%	1209486	27	83	12322013	13.684%
	S_D_80%&M_40%	1131413	29	87	12304441	13.807%

S_D_80%&M_50%	1029426	32	93	12266894	14.070%

* M: mask wearing rate; S_D: social distance

Table.S11 Simulation of different isolation ratios in 4 cities

	Measure	Max cases	Get max cases time	Duration time	All cases	Decline rate
Shanghai City	No Intervention	1836285	49	116	24333150	0.000%
	I_R_19.07%	1478529	56	132	23860482	1.942%
	I_R_27.47%	1386432	57	135	23424230	3.735%
	I_R_32.19%	1323408	58	138	23110862	5.023%
	I_R_36.35%	1261151	59	140	22780173	6.382%
	I_R_40.04%	1199448	60	143	22432771	7.810%
	I_R_43.35%	1138001	61	146	22069250	9.304%
	I_R_46.32%	1081483	63	150	21690185	10.862%
	I_R_49.00%	1024987	64	153	21296128	12.481%
	I_R_51.44%	967565	65	157	20887610	14.160%
	I_R_53.66%	914136	67	161	20465135	15.896%
	I_R_55.70%	860128	68	166	20029185	17.688%
	I_R_57.57%	809220	70	170	19580215	19.533%
	I_R_59.29%	758387	72	175	19118664	21.430%
	I_R_60.89%	709159	73	180	18644942	23.376%
	I_R_62.37%	662345	75	186	18159443	25.372%
	I_R_63.74%	616715	77	192	17662540	27.414%
	I_R_65.02%	572542	80	198	17154587	29.501%
	I_R_66.22%	530600	82	205	16635921	31.633%
	I_R_67.34%	489779	84	212	16106862	33.807%
	I_R_68.39%	450711	87	220	15567716	36.023%
	I_R_69.37%	413100	90	228	15018772	38.279%
	I_R_70.30%	377322	93	237	14460309	40.574%
	I_R_71.18%	343319	96	247	13892590	42.907%
	I_R_72.01%	310812	99	258	13315869	45.277%
	I_R_72.79%	280097	103	270	12730386	47.683%
	I_R_73.53%	250973	107	283	12136376	50.124%
	I_R_74.23%	223514	112	297	11534061	52.599%
Shenzhen City	No Intervention	630390	87	182	15236301	0.000%
	I_R_30.14%	549888	92	191	14385616	5.583%
	I_R_35.26%	499429	95	199	13801988	9.414%
	I_R_39.76%	451198	99	207	13207010	13.319%
	I_R_43.74%	405589	103	217	12602062	17.289%
	I_R_47.28%	362561	107	227	11988354	21.317%
	I_R_50.45%	322027	112	238	11366953	25.396%
	I_R_53.31%	283907	117	251	10738799	29.518%
	I_R_55.89%	248455	123	265	10104721	33.680%
	I_R_58.23%	215569	130	282	9465451	37.876%

	I_R_60.36%	185086	138	300	8821637	42.101%
	I_R_62.32%	157127	146	321	8173855	46.353%
	I_R_64.11%	131620	156	345	7522615	50.627%
	I_R_65.76%	108535	168	374	6868372	54.921%
	I_R_67.28%	87802	182	408	6211530	59.232%
	I_R_68.69%	69408	198	449	5552454	63.558%
	I_R_70.00%	53292	219	500	4891468	67.896%
	I_R_71.21%	39420	244	565	4228870	72.245%
	I_R_72.34%	27724	277	651	3564932	76.602%
	I_R_73.40%	18161	323	769	2899919	80.967%
	I_R_74.37%	10676	389	946	2234114	85.337%
	I_R_75.28%	5214	497	993	1565937	89.722%
	I_R_76.06%	1722	706	993	814311	94.655%
	I_R_76.77%	140	1001	993	85759	99.437%
	I_R_77.06%	37	31	993	10191	99.933%
	I_R_77.08%	36	30	422	4262	99.972%
Nanjing City	No Intervention	839297	31	87	8993194	0.000%
	I_R_30.14%	824605	30	86	8816204	1.968%
	I_R_35.27%	802865	31	87	8699331	3.268%
	I_R_39.77%	781489	31	88	8584562	4.544%
	I_R_43.75%	754434	32	88	8471645	5.799%
	I_R_47.29%	737602	32	89	8360372	7.037%
	I_R_50.46%	712059	32	90	8250567	8.258%
	I_R_53.32%	694635	33	91	8142082	9.464%
	I_R_55.90%	672325	33	92	8034793	10.657%
	I_R_58.24%	653459	34	93	7928593	11.838%
	I_R_60.38%	633184	34	94	7823394	13.008%
	I_R_62.33%	614381	35	95	7719115	14.167%
	I_R_64.12%	595123	35	96	7615690	15.317%
	I_R_65.78%	577335	36	98	7513058	16.458%
	I_R_67.30%	558250	36	99	7411166	17.591%
	I_R_68.71%	542027	37	100	7309967	18.717%
	I_R_70.02%	522435	37	101	7209418	19.835%
	I_R_71.24%	508034	38	103	7109483	20.946%
	I_R_72.37%	490210	39	104	7010125	22.051%
	I_R_73.43%	474888	39	105	6911314	23.150%
	I_R_74.43%	459594	40	107	6813021	24.242%
	I_R_75.36%	442528	41	108	6715219	25.330%
	I_R_76.23%	429117	41	110	6617885	26.412%
	I_R_77.05%	414614	42	111	6520995	27.490%
	I_R_77.82%	399258	43	113	6424528	28.562%

	I_R_78.55%	386003	43	115	6328466	29.630%
	I_R_79.24%	372909	44	117	6232790	30.694%
Suzhou City	No Intervention	1770814	15	24	14275397	0.000%
	I_R_30.14%	1722336	15	68	12576506	11.901%
	I_R_35.27%	1689062	16	69	12200997	14.531%
	I_R_39.77%	1677870	16	69	11958595	16.229%
	I_R_43.75%	1665865	16	69	11774021	17.522%
	I_R_47.29%	1652978	16	69	11616792	18.624%
	I_R_50.46%	1639110	16	69	11474529	19.620%
	I_R_53.32%	1624145	16	69	11341804	20.550%
	I_R_55.90%	1607966	16	69	11215995	21.431%
	I_R_58.24%	1590461	16	69	11095703	22.274%
	I_R_60.38%	1571529	16	69	10980105	23.084%
	I_R_62.33%	1551088	16	69	10868665	23.864%
	I_R_64.13%	1529073	16	69	10761003	24.619%
	I_R_65.78%	1505440	16	69	10656832	25.348%
	I_R_67.30%	1480167	16	69	10555917	26.055%
	I_R_68.71%	1453255	16	69	10458063	26.741%
	I_R_70.02%	1424725	16	69	10363101	27.406%
	I_R_71.24%	1412040	17	69	10270877	28.052%
	I_R_72.38%	1403874	17	69	10181253	28.680%
	I_R_73.44%	1394403	17	69	10094105	29.290%
	I_R_74.43%	1383528	17	69	10009314	29.884%
	I_R_75.36%	1371162	17	70	9926773	30.462%
	I_R_76.23%	1357231	17	70	9846377	31.026%
	I_R_77.05%	1341677	17	70	9768031	31.574%
	I_R_77.83%	1324458	17	70	9691646	32.109%
	I_R_78.56%	1305552	17	70	9617135	32.631%
	I_R_79.25%	1284953	17	70	9544419	33.141%

* I_R: isolation rate

Table.S12 Simulation of different combinations of mask wear, social distance rates and isolation rates in 4 cities

	Measure	Max cases	Get max cases time	Duration time	All cases	Decline rate
	No Intervention	1836285	49	116	24333150	0.000%
	M_20%&I_R_27.47%	1577573	53	125	23809552	2.152%
	S_D_40%&M_10%&I_R_27.47%	1467826	55	131	23608131	2.980%
	M_20%&I_R_32.19%	1518084	54	127	23563636	3.162%
	S_D_40%&M_10%&I_R_32.19%	1406973	56	133	23326207	4.138%
	M_50%&I_R_27.47%	1311071	59	140	23234905	4.513%
	M_20%&I_R_40.04%	1397658	56	131	23031496	5.349%
	M_50%&I_R_32.19%	1248041	60	142	22890255	5.930%
	S_D_40%&M_10%&I_R_40.04%	1286385	58	138	22715968	6.646%
	M_50%&I_R_40.04%	1122271	62	149	22144926	8.993%
	M_20%&I_R_49.00%	1225395	59	139	22138035	9.021%
	S_D_40%&M_10%&I_R_49.00%	1110321	61	147	21691990	10.854%
	M_20%&I_R_53.66%	1118260	61	145	21483200	11.712%
	S_D_50%&M_40%&I_R_27.47%	858790	73	178	21367008	12.190%
	S_D_40%&M_10%&I_R_53.66%	1002966	64	153	20942397	13.935%
	M_50%&I_R_49.00%	945215	67	160	20897177	14.121%
	M_20%&I_R_57.57%	1015498	63	151	20784280	14.585%
Shanghai City	S_D_50%&M_40%&I_R_32.19%	790798	75	184	20745842	14.742%
	M_20%&I_R_59.29%	964939	64	155	20419105	16.085%
	S_D_40%&M_10%&I_R_57.57%	897060	67	161	20143282	17.219%
	M_20%&I_R_60.89%	914483	65	158	20043864	17.627%
	M_50%&I_R_53.66%	835561	70	169	19986209	17.864%
	S_D_60%&M_40%&I_R_27.47%	640558	84	208	19783042	18.699%
	S_D_40%&M_10%&I_R_59.29%	847149	68	165	19726141	18.933%
	M_20%&I_R_62.37%	866858	67	162	19658849	19.210%
	S_D_50%&M_40%&I_R_40.04%	662886	81	198	19412983	20.220%
	S_D_40%&M_10%&I_R_60.89%	797449	70	170	19297771	20.693%
	M_20%&I_R_63.74%	820357	68	166	19264340	20.831%
	M_50%&I_R_57.57%	730895	73	179	19017193	21.847%
	S_D_60%&M_40%&I_R_32.19%	574115	88	218	18950731	22.120%
	S_D_40%&M_10%&I_R_62.37%	749787	71	175	18858517	22.499%
	M_50%&I_R_59.29%	681186	75	185	18512183	23.922%
	S_D_40%&M_10%&I_R_63.74%	703809	73	180	18408711	24.347%
	S_D_80%&I_R_27.47%	488384	96	240	18218778	25.128%
	M_50%&I_R_60.89%	632887	77	191	17994125	26.051%
	M_50%&I_R_62.37%	586189	80	198	17463457	28.232%

S_D_50%&M_40%&I_R_49.00%	490188	90	225	17209093	29.277%
S_D_80%&I_R_32.19%	424414	101	255	17189089	29.359%
S_D_60%&M_40%&I_R_40.04%	450657	96	242	17174155	29.421%
M_50%&I_R_63.74%	541977	82	205	16920590	30.463%
S_D_80%&M_10%&I_R_27.47%	384966	106	271	16825971	30.852%
S_D_80%&M_10%&I_R_32.19%	324037	113	291	15626959	35.779%
S_D_50%&M_40%&I_R_53.66%	388220	98	249	15617992	35.816%
S_D_80%&I_R_40.04%	308546	113	291	15000021	38.356%
S_D_60%&M_40%&I_R_49.00%	291192	112	291	14258717	41.402%
S_D_50%&M_40%&I_R_57.57%	297764	108	279	13939297	42.715%
S_D_70%&M_50%&I_R_27.47%	210717	138	363	13438941	44.771%
S_D_80%&M_10%&I_R_40.04%	216774	131	344	13085358	46.224%
S_D_50%&M_40%&I_R_59.29%	256947	114	297	13069398	46.290%
S_D_80%&M_30%&I_R_27.47%	169534	151	402	12314629	49.392%
S_D_50%&M_40%&I_R_60.89%	219140	120	318	12180243	49.944%
S_D_60%&M_40%&I_R_53.66%	203650	128	338	12167380	49.997%
S_D_70%&M_50%&I_R_32.19%	159668	152	407	11845736	51.319%
S_D_80%&I_R_49.00%	167693	140	376	11428156	53.035%
S_D_50%&M_40%&I_R_62.37%	184397	128	341	11272603	53.674%
S_D_80%&M_30%&I_R_32.19%	122387	169	459	10594819	56.459%
S_D_50%&M_40%&I_R_63.74%	152635	136	369	10347218	57.477%
S_D_60%&M_40%&I_R_57.57%	131522	149	405	9970914	59.023%
S_D_80%&M_10%&I_R_49.00%	95039	174	483	8955706	63.195%
S_D_80%&I_R_53.66%	97146	169	469	8878506	63.513%
S_D_60%&M_40%&I_R_59.29%	101376	162	450	8836351	63.686%
S_D_70%&M_50%&I_R_40.04%	78167	194	544	8487508	65.120%
S_D_60%&M_40%&I_R_60.89%	75183	179	508	7679155	68.442%
S_D_80%&M_30%&I_R_40.04%	50643	226	653	6975860	71.332%
S_D_60%&M_40%&I_R_62.37%	52953	201	584	6500579	73.285%
S_D_80%&I_R_57.57%	45780	216	634	6211117	74.475%
S_D_80%&M_10%&I_R_53.66%	41278	228	673	6019861	75.261%
S_D_60%&M_40%&I_R_63.74%	34676	229	689	5302106	78.210%
S_D_80%&I_R_59.29%	27343	253	774	4838397	80.116%
S_D_80%&I_R_60.89%	13730	308	999	3444462	85.845%
S_D_70%&M_50%&I_R_49.00%	9882	360	999	3088012	87.309%
S_D_80%&M_10%&I_R_57.57%	9832	344	999	2966782	87.808%
S_D_80%&M_50%&I_R_27.47%	5409	464	999	2453135	89.919%
S_D_80%&I_R_62.37%	4919	391	999	2033912	91.641%
S_D_80%&M_10%&I_R_59.29%	2472	443	999	1392891	94.276%
S_D_80%&M_30%&I_R_49.00%	1733	484	999	1142734	95.304%
S_D_80%&I_R_63.74%	880	360	999	643651	97.355%

	S_D_80%&M_10%&I_R_60.89%	572	24	999	244958	98.993%
	S_D_80%&M_50%&I_R_32.19%	545	24	999	222325	99.086%
	S_D_70%&M_50%&I_R_53.66%	562	24	999	148962	99.388%
	S_D_80%&M_10%&I_R_62.37%	570	24	789	73106	99.700%
	S_D_80%&M_30%&I_R_53.66%	557	24	577	50310	99.793%
	S_D_80%&M_10%&I_R_63.74%	569	24	460	42104	99.827%
	S_D_70%&M_50%&I_R_57.57%	558	24	378	33355	99.863%
	S_D_80%&M_50%&I_R_40.04%	544	23	345	27960	99.885%
	S_D_70%&M_50%&I_R_59.29%	557	24	279	24742	99.898%
	S_D_80%&M_30%&I_R_57.57%	554	24	269	23604	99.903%
	S_D_70%&M_50%&I_R_60.89%	555	24	225	20004	99.918%
	S_D_80%&M_30%&I_R_59.29%	552	24	217	19166	99.921%
	S_D_70%&M_50%&I_R_62.37%	553	24	190	17008	99.930%
	S_D_80%&M_30%&I_R_60.89%	551	24	184	16347	99.933%
	S_D_70%&M_50%&I_R_63.74%	552	24	167	14944	99.939%
	S_D_80%&M_30%&I_R_62.37%	549	24	162	14400	99.941%
	S_D_80%&M_50%&I_R_49.00%	544	23	165	13877	99.943%
	S_D_80%&M_30%&I_R_63.74%	548	24	145	12972	99.947%
	S_D_80%&M_50%&I_R_53.66%	544	23	129	11054	99.955%
	S_D_80%&M_50%&I_R_57.57%	544	23	109	9463	99.961%
	S_D_80%&M_50%&I_R_59.29%	544	23	102	8902	99.963%
	S_D_80%&M_50%&I_R_60.89%	544	23	96	8441	99.965%
	S_D_80%&M_50%&I_R_62.37%	544	23	91	8055	99.967%
	S_D_80%&M_50%&I_R_63.74%	544	23	87	7728	99.968%
Shenzhen City	No Intervention	630390	87	182	15236301	0.000%
	M_20%&I_R_30.14%	442123	103	217	13564631	10.972%
	S_D_40%&M_10%&I_R_30.14%	377722	111	236	12942175	15.057%
	M_20%&I_R_35.26%	392963	107	227	12879248	15.470%
	S_D_40%&M_10%&I_R_35.26%	329864	117	250	12183164	20.039%
	M_50%&I_R_30.14%	288859	127	273	11864473	22.130%
	M_20%&I_R_43.74%	303397	118	253	11470734	24.714%
	M_50%&I_R_35.26%	243885	135	293	10983036	27.915%
	S_D_40%&M_10%&I_R_43.74%	243943	131	283	10624174	30.271%
	M_20%&I_R_53.31%	191282	141	306	9285278	39.058%
	M_50%&I_R_43.74%	165309	156	344	9174485	39.785%
	S_D_40%&M_10%&I_R_53.31%	139922	161	356	8207190	46.134%
	M_20%&I_R_58.23%	131507	162	358	7792856	48.853%
	S_D_50%&M_40%&I_R_30.14%	79210	227	514	7159720	53.009%
	S_D_40%&M_10%&I_R_58.23%	87111	193	433	6557816	56.959%
	M_50%&I_R_53.31%	76613	208	471	6374796	58.160%
	M_20%&I_R_62.32%	83388	192	431	6279878	58.783%

S_D_50%&M_40%&I_R_35.26%	50905	267	615	5786585	62.021%
M_20%&I_R_64.11%	63625	212	482	5517313	63.788%
S_D_40%&M_10%&I_R_62.32%	47202	241	556	4886565	67.928%
M_20%&I_R_65.76%	46646	237	546	4751429	68.815%
M_50%&I_R_58.23%	36646	274	635	4466719	70.684%
S_D_40%&M_10%&I_R_64.11%	31954	278	650	4044541	73.455%
M_20%&I_R_67.28%	32405	271	632	3982710	73.860%
S_D_60%&M_40%&I_R_30.14%	17147	420	993	3586231	76.463%
M_20%&I_R_68.69%	20839	318	754	3211609	78.921%
S_D_40%&M_10%&I_R_65.76%	19761	331	787	3199093	79.003%
S_D_50%&M_40%&I_R_43.74%	13104	437	993	2981127	80.434%
M_50%&I_R_62.32%	11520	414	993	2535340	83.360%
S_D_40%&M_10%&I_R_67.28%	10553	413	993	2350832	84.571%
S_D_60%&M_40%&I_R_35.26%	4579	664	993	1799218	88.191%
M_50%&I_R_64.11%	4338	577	993	1549082	89.833%
S_D_40%&M_10%&I_R_68.69%	4261	561	993	1490139	90.220%
M_50%&I_R_65.76%	630	1001	993	279668	98.164%
S_D_80%&I_R_30.14%	84	1001	993	55918	99.633%
M_50%&I_R_67.28%	37	31	993	12649	99.917%
S_D_50%&M_40%&I_R_53.31%	36	27	493	4821	99.968%
S_D_60%&M_40%&I_R_43.74%	36	27	444	4248	99.972%
S_D_80%&I_R_35.26%	37	27	439	4109	99.973%
M_50%&I_R_68.69%	36	29	405	4064	99.973%
S_D_80%&M_10%&I_R_30.14%	37	27	324	2993	99.980%
S_D_80%&M_10%&I_R_35.26%	36	27	186	1738	99.989%
S_D_50%&M_40%&I_R_58.23%	36	27	174	1708	99.989%
S_D_80%&I_R_43.74%	36	27	152	1450	99.990%
S_D_60%&M_40%&I_R_53.31%	36	27	126	1217	99.992%
S_D_50%&M_40%&I_R_62.32%	35	26	114	1121	99.993%
S_D_70%&M_50%&I_R_30.14%	36	27	120	1113	99.993%
S_D_80%&M_10%&I_R_43.74%	36	27	109	1037	99.993%
S_D_80%&M_30%&I_R_30.14%	36	27	105	974	99.994%
S_D_50%&M_40%&I_R_64.11%	35	26	99	975	99.994%
S_D_70%&M_50%&I_R_35.26%	36	27	98	921	99.994%
S_D_60%&M_40%&I_R_58.23%	35	26	92	897	99.994%
S_D_50%&M_40%&I_R_65.76%	35	26	89	872	99.994%
S_D_80%&I_R_53.31%	35	27	88	850	99.994%
S_D_80%&M_30%&I_R_35.26%	36	27	89	832	99.995%
S_D_50%&M_40%&I_R_67.28%	35	26	81	794	99.995%
S_D_60%&M_40%&I_R_62.32%	35	26	76	737	99.995%
S_D_50%&M_40%&I_R_68.69%	35	26	75	734	99.995%

S_D_70%&M_50%&I_R_43.74%	35	27	76	719	99.995%
S_D_80%&M_10%&I_R_53.31%	35	26	74	717	99.995%
S_D_80%&I_R_58.23%	35	26	72	702	99.995%
S_D_60%&M_40%&I_R_64.11%	35	26	70	684	99.996%
S_D_80%&M_30%&I_R_43.74%	35	27	70	671	99.996%
S_D_60%&M_40%&I_R_65.76%	35	26	66	641	99.996%
S_D_80%&M_10%&I_R_58.23%	35	26	64	619	99.996%
S_D_80%&I_R_62.32%	35	26	63	613	99.996%
S_D_60%&M_40%&I_R_67.28%	35	26	62	606	99.996%
S_D_80%&M_50%&I_R_30.14%	35	27	64	599	99.996%
S_D_80%&I_R_64.11%	35	26	60	581	99.996%
S_D_60%&M_40%&I_R_68.69%	35	26	59	576	99.996%
S_D_70%&M_50%&I_R_53.31%	35	26	60	576	99.996%
S_D_80%&M_50%&I_R_35.26%	35	27	59	559	99.996%
S_D_80%&M_10%&I_R_62.32%	35	26	57	556	99.996%
S_D_80%&I_R_65.76%	35	26	57	554	99.996%
S_D_80%&M_30%&I_R_53.31%	35	26	57	551	99.996%
S_D_80%&M_10%&I_R_64.11%	35	26	55	532	99.997%
S_D_80%&I_R_67.28%	35	26	55	532	99.997%
S_D_70%&M_50%&I_R_58.23%	35	26	54	522	99.997%
S_D_80%&I_R_68.69%	35	26	53	512	99.997%
S_D_80%&M_10%&I_R_65.76%	35	26	53	511	99.997%
S_D_80%&M_30%&I_R_58.23%	35	26	52	504	99.997%
S_D_80%&M_50%&I_R_43.74%	35	26	53	503	99.997%
S_D_80%&M_10%&I_R_67.28%	35	26	51	493	99.997%
S_D_70%&M_50%&I_R_62.32%	35	26	50	484	99.997%
S_D_80%&M_10%&I_R_68.69%	35	26	49	478	99.997%
S_D_80%&M_30%&I_R_62.32%	35	26	48	469	99.997%
S_D_70%&M_50%&I_R_64.11%	35	26	48	468	99.997%
S_D_80%&M_30%&I_R_64.11%	35	26	47	456	99.997%
S_D_70%&M_50%&I_R_65.76%	35	26	47	455	99.997%
S_D_80%&M_50%&I_R_53.31%	35	26	47	451	99.997%
S_D_80%&M_30%&I_R_65.76%	35	26	46	444	99.997%
S_D_70%&M_50%&I_R_67.28%	35	26	46	443	99.997%
S_D_80%&M_30%&I_R_67.28%	35	26	45	433	99.997%
S_D_70%&M_50%&I_R_68.69%	35	26	45	433	99.997%
S_D_80%&M_50%&I_R_58.23%	35	26	44	426	99.997%
S_D_80%&M_30%&I_R_68.69%	35	26	44	423	99.997%
S_D_80%&M_50%&I_R_62.32%	35	26	42	407	99.997%
S_D_80%&M_50%&I_R_64.11%	35	26	41	399	99.997%
S_D_80%&M_50%&I_R_65.76%	35	26	40	392	99.997%

	S_D_80%&M_50%&I_R_67.28%	35	26	40	386	99.997%
	S_D_80%&M_50%&I_R_68.69%	35	26	39	379	99.998%
	No Intervention	839297	31	87	8993194	0.000%
	M_20%&I_R_30.14%	766922	33	90	8796474	2.187%
	S_D_40%&M_10%&I_R_30.14%	737321	34	93	8778532	2.387%
	M_50%&I_R_30.14%	681636	36	98	8742148	2.792%
	M_20%&I_R_35.27%	748718	33	91	8674173	3.547%
	S_D_40%&M_10%&I_R_35.27%	708749	35	94	8651720	3.797%
	M_50%&I_R_35.27%	659650	37	99	8606970	4.295%
	S_D_50%&M_40%&I_R_30.14%	524590	46	118	8524850	5.208%
	M_20%&I_R_43.75%	702061	34	93	8433766	6.221%
	S_D_40%&M_10%&I_R_43.75%	663521	36	96	8401016	6.585%
	S_D_50%&M_40%&I_R_35.27%	498825	47	120	8348813	7.165%
	M_50%&I_R_43.75%	608756	38	102	8337556	7.290%
	S_D_60%&M_40%&I_R_30.14%	443890	52	132	8315710	7.533%
	S_D_60%&M_40%&I_R_35.27%	415771	54	135	8106254	9.862%
	S_D_80%&I_R_30.14%	382941	58	145	8096399	9.972%
	M_20%&I_R_53.32%	632974	36	96	8081149	10.141%
	S_D_40%&M_10%&I_R_53.32%	594700	37	100	8030296	10.707%
	S_D_50%&M_40%&I_R_43.75%	448310	49	125	7990972	11.144%
Nanjing City	M_50%&I_R_53.32%	541266	41	107	7934675	11.770%
	S_D_80%&M_10%&I_R_30.14%	339186	63	157	7893531	12.228%
	S_D_80%&I_R_35.27%	355734	60	149	7854713	12.659%
	M_20%&I_R_58.24%	593091	37	99	7850121	12.710%
	S_D_40%&M_10%&I_R_58.24%	555508	39	103	7785756	13.426%
	S_D_60%&M_40%&I_R_43.75%	365244	57	143	7677584	14.629%
	M_50%&I_R_58.24%	499419	42	110	7666487	14.752%
	S_D_80%&M_10%&I_R_35.27%	311661	65	162	7623639	15.229%
	M_20%&I_R_62.33%	554315	38	102	7621668	15.251%
	S_D_40%&M_10%&I_R_62.33%	515357	40	106	7542846	16.127%
	M_20%&I_R_64.13%	533082	39	103	7508283	16.511%
	S_D_50%&M_40%&I_R_53.32%	377373	53	134	7441692	17.252%
	S_D_40%&M_10%&I_R_64.13%	496337	41	108	7421922	17.472%
	M_50%&I_R_62.33%	459218	44	114	7398482	17.732%
	M_20%&I_R_65.78%	515863	39	105	7395408	17.767%
	S_D_70%&M_50%&I_R_30.14%	257503	75	185	7378930	17.950%
	S_D_80%&I_R_43.75%	304127	64	160	7358355	18.179%
	S_D_40%&M_10%&I_R_65.78%	476645	42	110	7301320	18.813%
	M_20%&I_R_67.30%	497984	40	106	7283007	19.016%
	M_50%&I_R_64.13%	439582	45	116	7264536	19.222%
	S_D_80%&M_30%&I_R_30.14%	235714	80	195	7202951	19.907%

S_D_40%&M_10%&I_R_67.30%	457419	42	112	7181019	20.151%
M_20%&I_R_68.71%	478714	41	108	7171053	20.261%
M_50%&I_R_65.78%	420486	46	119	7130629	20.711%
S_D_50%&M_40%&I_R_58.24%	333837	56	142	7068568	21.401%
S_D_80%&M_10%&I_R_43.75%	261105	71	175	7068387	21.403%
S_D_40%&M_10%&I_R_68.71%	440655	43	113	7061003	21.485%
S_D_70%&M_50%&I_R_35.27%	231049	79	194	7041893	21.698%
S_D_60%&M_40%&I_R_53.32%	294764	63	156	7013996	22.008%
M_50%&I_R_67.30%	401994	47	121	6996760	22.199%
M_50%&I_R_68.71%	384114	48	123	6862932	23.687%
S_D_80%&M_30%&I_R_35.27%	209705	84	205	6843987	23.898%
S_D_50%&M_40%&I_R_62.33%	293870	60	150	6690899	25.600%
S_D_80%&I_R_53.32%	235014	72	178	6586821	26.758%
S_D_60%&M_40%&I_R_58.24%	252024	68	167	6560475	27.051%
S_D_50%&M_40%&I_R_64.13%	274701	62	154	6500597	27.716%
S_D_70%&M_50%&I_R_43.75%	182313	88	215	6347345	29.421%
S_D_50%&M_40%&I_R_65.78%	256378	64	159	6309431	29.842%
S_D_80%&M_10%&I_R_53.32%	193545	81	199	6203653	31.018%
S_D_50%&M_40%&I_R_67.30%	238883	66	164	6117477	31.977%
S_D_80%&M_30%&I_R_43.75%	161921	94	229	6104162	32.125%
S_D_60%&M_40%&I_R_62.33%	213569	73	180	6099792	32.173%
S_D_80%&I_R_58.24%	194357	79	194	6058074	32.637%
S_D_50%&M_40%&I_R_68.71%	222083	68	169	5924809	34.119%
S_D_60%&M_40%&I_R_64.13%	195515	76	187	5867159	34.760%
S_D_60%&M_40%&I_R_65.78%	178423	79	195	5633186	37.362%
S_D_80%&M_10%&I_R_58.24%	154461	90	220	5610348	37.616%
S_D_80%&M_50%&I_R_30.14%	108942	123	298	5586969	37.876%
S_D_80%&I_R_62.33%	157629	86	212	5520178	38.618%
S_D_60%&M_40%&I_R_67.30%	162110	82	203	5397997	39.977%
S_D_70%&M_50%&I_R_53.32%	120248	105	257	5264064	41.466%
S_D_80%&I_R_64.13%	141005	91	223	5248327	41.641%
S_D_60%&M_40%&I_R_68.71%	146777	86	212	5161706	42.604%
S_D_80%&M_50%&I_R_35.27%	87168	135	328	5040863	43.948%
S_D_80%&M_10%&I_R_62.33%	120128	100	245	5006447	44.331%
S_D_80%&I_R_65.78%	125259	96	235	4974785	44.683%
S_D_80%&M_30%&I_R_53.32%	102069	115	280	4950282	44.955%
S_D_80%&M_10%&I_R_64.13%	104687	106	260	4701155	47.725%
S_D_80%&I_R_67.30%	110605	101	248	4699717	47.741%
S_D_70%&M_50%&I_R_58.24%	86374	121	296	4520391	49.735%
S_D_80%&I_R_68.71%	96909	107	262	4423271	50.815%
S_D_80%&M_10%&I_R_65.78%	90403	113	277	4393926	51.142%

	S_D_80%&M_30%&I_R_58.24%	70101	134	328	4158269	53.762%
	S_D_80%&M_10%&I_R_67.30%	77237	121	296	4084953	54.577%
	S_D_80%&M_50%&I_R_43.75%	50919	168	411	3917269	56.442%
	S_D_80%&M_10%&I_R_68.71%	65188	130	318	3774415	58.030%
	S_D_70%&M_50%&I_R_62.33%	58357	143	350	3763425	58.153%
	S_D_70%&M_50%&I_R_64.13%	46501	157	385	3380804	62.407%
	S_D_80%&M_30%&I_R_62.33%	44387	162	397	3352253	62.725%
	S_D_70%&M_50%&I_R_65.78%	36065	173	427	2995814	66.688%
	S_D_80%&M_30%&I_R_64.13%	33818	180	445	2944909	67.254%
	S_D_70%&M_50%&I_R_67.30%	27017	194	481	2608719	70.992%
	S_D_80%&M_30%&I_R_65.78%	24749	204	505	2535098	71.811%
	S_D_70%&M_50%&I_R_68.71%	19329	221	551	2219762	75.317%
	S_D_80%&M_50%&I_R_53.32%	14927	272	678	2170606	75.864%
	S_D_80%&M_30%&I_R_67.30%	17146	235	586	2123112	76.392%
	S_D_80%&M_30%&I_R_68.71%	10983	277	700	1709236	80.994%
	S_D_80%&M_50%&I_R_58.24%	2947	481	1001	974844	89.160%
	S_D_80%&M_50%&I_R_62.33%	41	8	1001	12205	99.864%
	S_D_80%&M_50%&I_R_64.13%	41	8	371	3819	99.958%
	S_D_80%&M_50%&I_R_65.78%	40	8	223	2306	99.974%
	S_D_80%&M_50%&I_R_67.30%	40	8	163	1682	99.981%
	S_D_80%&M_50%&I_R_68.71%	40	8	129	1340	99.985%
Suzhou City	No Intervention	1770814	15	24	14275397	0.000%
	M_20%&I_R_30.14%	1711616	16	69	12175153	14.712%
	S_D_40%&M_10%&I_R_30.14%	1664174	17	70	12142163	14.943%
	M_50%&I_R_30.14%	1616037	17	71	12127449	15.047%
	S_D_50%&M_40%&I_R_30.14%	1493611	20	73	12112975	15.148%
	S_D_60%&M_40%&I_R_30.14%	1399384	22	75	12107810	15.184%
	S_D_80%&I_R_30.14%	1344866	23	77	12103195	15.216%
	S_D_80%&M_10%&I_R_30.14%	1294797	24	79	12098630	15.248%
	S_D_70%&M_50%&I_R_30.14%	1187005	26	82	12084070	15.350%
	S_D_80%&M_30%&I_R_30.14%	1167531	27	84	12077966	15.393%
	M_20%&I_R_35.26%	1683554	16	69	12009180	15.875%
	S_D_80%&M_50%&I_R_30.14%	973993	33	94	11996730	15.962%
	S_D_40%&M_10%&I_R_35.26%	1648893	17	70	11989164	16.015%
	M_50%&I_R_35.26%	1580302	18	71	11977900	16.094%
	S_D_50%&M_40%&I_R_35.26%	1463328	20	74	11964049	16.191%
	S_D_60%&M_40%&I_R_35.26%	1379658	22	75	11958307	16.231%
	S_D_80%&I_R_35.26%	1311659	23	77	11952651	16.271%
	S_D_80%&M_10%&I_R_35.26%	1257479	24	79	11946793	16.312%
	S_D_70%&M_50%&I_R_35.26%	1162141	27	83	11927863	16.445%
	S_D_80%&M_30%&I_R_35.26%	1125725	27	84	11919983	16.500%

S_D_80%&M_50%&I_R_35.26%	943594	33	95	11818523	17.211%
M_20%&I_R_43.74%	1621845	16	70	11713724	17.945%
S_D_40%&M_10%&I_R_43.74%	1613381	17	70	11703251	18.018%
M_50%&I_R_43.74%	1554467	18	71	11695083	18.075%
S_D_50%&M_40%&I_R_43.74%	1389390	20	74	11681369	18.171%
S_D_60%&M_40%&I_R_43.74%	1322748	22	76	11673739	18.225%
S_D_80%&I_R_43.74%	1252747	24	78	11665079	18.285%
S_D_80%&M_10%&I_R_43.74%	1206286	25	80	11655727	18.351%
S_D_70%&M_50%&I_R_43.74%	1095705	27	84	11625757	18.561%
S_D_80%&M_30%&I_R_43.74%	1073168	28	86	11613576	18.646%
S_D_80%&M_50%&I_R_43.74%	875560	34	98	11465526	19.683%
M_20%&I_R_53.31%	1558031	17	70	11319734	20.705%
S_D_40%&M_10%&I_R_53.31%	1545546	17	70	11313165	20.751%
M_50%&I_R_53.31%	1499072	18	71	11306529	20.797%
S_D_50%&M_40%&I_R_53.31%	1338262	21	74	11290732	20.908%
S_D_60%&M_40%&I_R_53.31%	1246389	23	77	11278073	20.996%
S_D_80%&I_R_53.31%	1166044	24	79	11262445	21.106%
S_D_80%&M_10%&I_R_53.31%	1120171	26	81	11245598	21.224%
S_D_70%&M_50%&I_R_53.31%	1009949	29	86	11193718	21.587%
S_D_80%&M_30%&I_R_53.31%	982193	29	88	11173382	21.730%
M_20%&I_R_58.23%	1531697	17	70	11080277	22.382%
S_D_40%&M_10%&I_R_58.23%	1489524	17	70	11074600	22.422%
M_50%&I_R_58.23%	1449397	18	71	11068225	22.466%
S_D_50%&M_40%&I_R_58.23%	1282052	21	75	11049431	22.598%
S_D_60%&M_40%&I_R_58.23%	1193012	23	78	11031818	22.721%
S_D_80%&I_R_58.23%	1125610	25	80	11009924	22.875%
S_D_80%&M_10%&I_R_58.23%	1066725	26	83	10986676	23.038%
S_D_80%&M_50%&I_R_53.31%	783694	36	102	10941975	23.351%
S_D_70%&M_50%&I_R_58.23%	959788	29	88	10917004	23.526%
S_D_80%&M_30%&I_R_58.23%	930606	30	90	10890241	23.713%
M_20%&I_R_62.32%	1498766	17	70	10856236	23.951%
S_D_40%&M_10%&I_R_62.32%	1435312	18	70	10850956	23.988%
M_50%&I_R_62.32%	1389021	18	71	10844481	24.034%
S_D_50%&M_40%&I_R_62.32%	1235117	22	75	10821279	24.196%
S_D_60%&M_40%&I_R_62.32%	1145057	24	78	10797426	24.363%
S_D_80%&I_R_62.32%	1064140	25	81	10768054	24.569%
M_20%&I_R_64.11%	1479543	17	70	10749439	24.700%
S_D_40%&M_10%&I_R_64.11%	1425638	18	71	10744241	24.736%
M_50%&I_R_64.11%	1368188	19	72	10737596	24.783%
S_D_80%&M_10%&I_R_62.32%	1020261	27	84	10737389	24.784%
S_D_50%&M_40%&I_R_64.11%	1219305	22	76	10711637	24.964%

S_D_60%&M_40%&I_R_64.11%	1125997	24	79	10684198	25.157%
S_D_80%&I_R_64.11%	1049320	26	82	10650661	25.391%
S_D_70%&M_50%&I_R_62.32%	909857	30	90	10647679	25.412%
M_20%&I_R_65.76%	1458377	17	70	10645877	25.425%
S_D_40%&M_10%&I_R_65.76%	1414088	18	71	10640696	25.461%
M_50%&I_R_65.76%	1357834	19	72	10633803	25.510%
S_D_80%&M_10%&I_R_64.11%	993417	27	84	10615936	25.635%
S_D_80%&M_30%&I_R_62.32%	878809	31	92	10613798	25.650%
S_D_50%&M_40%&I_R_65.76%	1199038	22	76	10604714	25.713%
S_D_80%&M_50%&I_R_58.23%	725571	38	105	10595546	25.778%
S_D_60%&M_40%&I_R_65.76%	1101245	24	79	10573390	25.933%
M_20%&I_R_67.28%	1435219	17	70	10545382	26.129%
S_D_40%&M_10%&I_R_67.28%	1400529	18	71	10540163	26.166%
S_D_80%&I_R_65.76%	1028004	26	82	10535419	26.199%
M_50%&I_R_67.28%	1345038	19	72	10532944	26.216%
S_D_70%&M_50%&I_R_64.11%	879102	31	91	10515437	26.339%
S_D_50%&M_40%&I_R_67.28%	1174297	22	76	10500350	26.444%
S_D_80%&M_10%&I_R_65.76%	965113	28	85	10496418	26.472%
S_D_80%&M_30%&I_R_64.11%	848907	32	93	10477761	26.603%
S_D_60%&M_40%&I_R_67.28%	1070944	24	80	10464851	26.693%
M_20%&I_R_68.69%	1410048	17	70	10447801	26.813%
S_D_40%&M_10%&I_R_68.69%	1384851	18	71	10442492	26.850%
M_50%&I_R_68.69%	1329677	19	72	10434867	26.903%
S_D_80%&I_R_67.28%	1000081	26	83	10422192	26.992%
S_D_50%&M_40%&I_R_68.69%	1145168	22	77	10398397	27.159%
S_D_70%&M_50%&I_R_65.76%	859746	31	92	10384650	27.255%
S_D_80%&M_10%&I_R_67.28%	947615	28	86	10378706	27.297%
S_D_60%&M_40%&I_R_68.69%	1052553	25	80	10358444	27.438%
S_D_80%&M_30%&I_R_65.76%	828210	32	94	10343033	27.546%
S_D_80%&I_R_68.69%	976621	27	84	10310853	27.772%
S_D_80%&M_10%&I_R_68.69%	922738	28	87	10262681	28.109%
S_D_70%&M_50%&I_R_67.28%	831287	31	93	10255215	28.162%
S_D_80%&M_50%&I_R_62.32%	670690	39	108	10250497	28.195%
S_D_80%&M_30%&I_R_67.28%	798584	33	95	10209518	28.482%
S_D_70%&M_50%&I_R_68.69%	810935	32	94	10127036	29.060%
S_D_80%&M_50%&I_R_64.11%	644432	40	110	10078365	29.400%
S_D_80%&M_30%&I_R_68.69%	779488	33	96	10077128	29.409%
S_D_80%&M_50%&I_R_65.76%	617987	41	112	9906441	30.605%
S_D_80%&M_50%&I_R_67.28%	591857	42	114	9734699	31.808%
S_D_80%&M_50%&I_R_68.69%	566378	43	116	9563112	33.010%

*M: mask wearing rate; S_D: social distance; I_R: isolation rate

Table.S13 Spillover risk values at different stages

Region	Transport		Colonization		Establishment		Landscape spread		Out
	Threshold	Actual value	Threshold	Actual value	Threshold	Actual value	Threshold	Actual value	Thr
Shanghai City	2022/3/1		2022/3/1–2022/3/6		2022/3/7–2022/3/23		2022/3/24–2022/4/1		202
	16.825	0.119	18.81	0.947	70.947	2.858	131.839	24.458	303
	(7.583–37.8)	(0.132–7.583)	(8.478–42.259)	(0.843–1.051)	(31.978–159.395)	(2.544–3.171)	(59.424–296.198)	(21.773–27.132)	(13 682
Shenzhen City	2022/1/31		2022/1/31–2022/2/5		2022/2/6–2022/2/17		2022/2/18–2022/3/2		202
	5.926	0.169	22.505	0.14	17.502	0.069	15.105	0.896	17.
	(13.314–2.671)	(0.187–0.15)	(10.143–50.56)	(0.156–0.125)	(7.889–39.322)	(0.076–0.061)	(6.808–33.936)	(0.994–0.798)	(8.0 40.
Nanjing City	2022/3/10		2022/3/10–2022/3/15		2022/3/16～2022/3/31		null		nul
	14.381	0.348	16.127	0.083	78.582	0.019	null	null	nul
	(6.482–32.309)	(0.31–0.386)	(7.269–36.233)	(0.074–0.092)	(35.419–176.548)	(0.017–0.021)	null	null	nul
Suzhou City	2022/2/10		2022/2/10–2022/2/15		2022/2/162022/3/2		null		nul
	14.947	0.067	14.91	0.033	26.611	0.147	null	null	nul
	(6.737–33.581)	(0.06–0.074)	(6.72–33.498)	(0.037–0.03)	(11.994–59.786)	(0.163–0.131)	null	null	nul

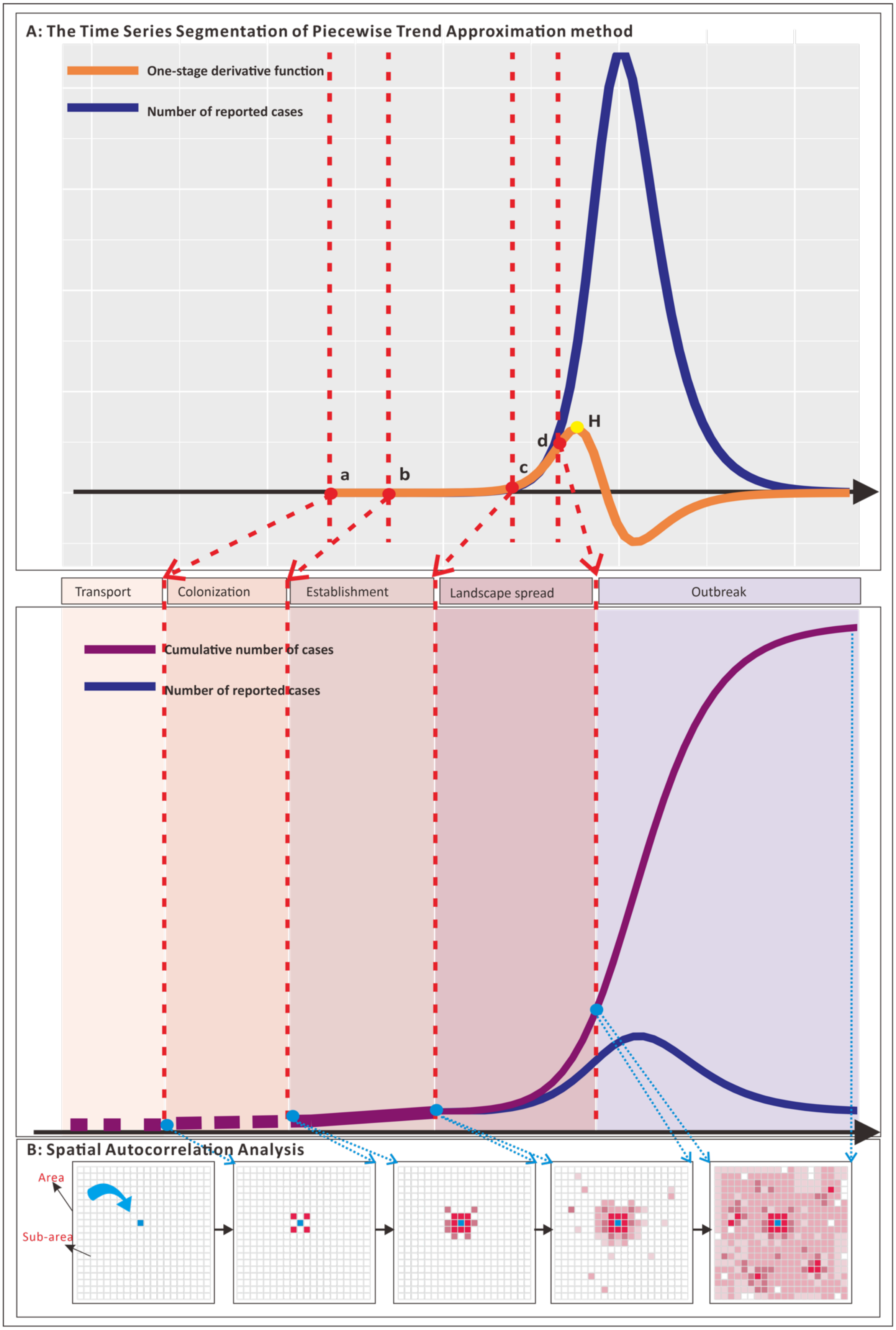

A: The Time Series Segmentation of Piecewise Trend Approximation method
One-stage derivative function
Number of reported cases
a
b
c
d
H
Transport
Colonization
Establishment
Landscape spread
Outbreak
Cumulative number of cases
Number of reported cases
B: Spatial Autocorrelation Analysis
Area
Sub-area

Fig.S1 Biological Invasion Theory Framework.

(a: Date of reporting of the first case; b: The time when the third-generation cases appeared, that is, a+6 days; c, d: According to the PTA methods, the case time series data of points 0-H are calculated in segments.)

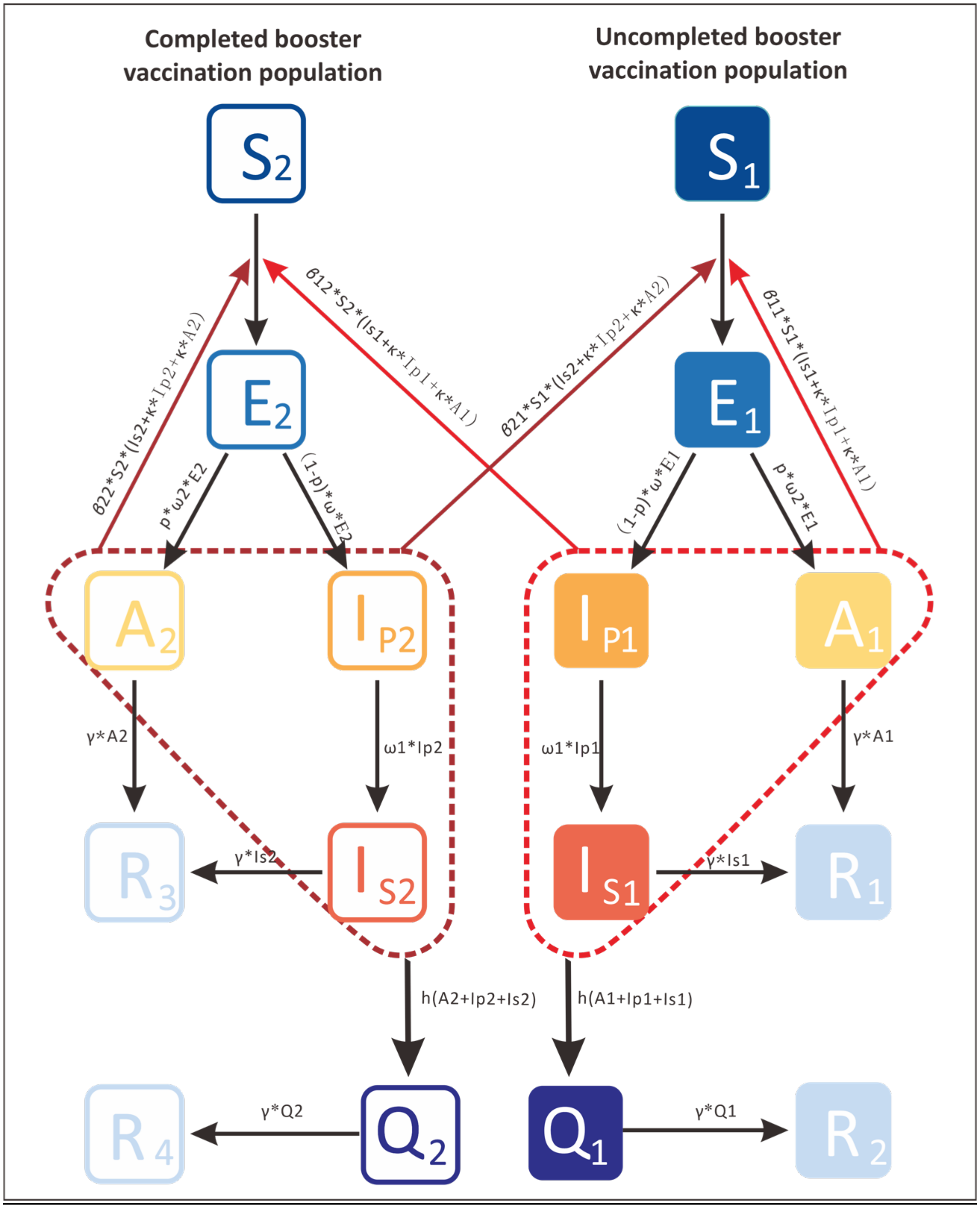

Fig.S2 SEIAR model with booster vaccination intervention

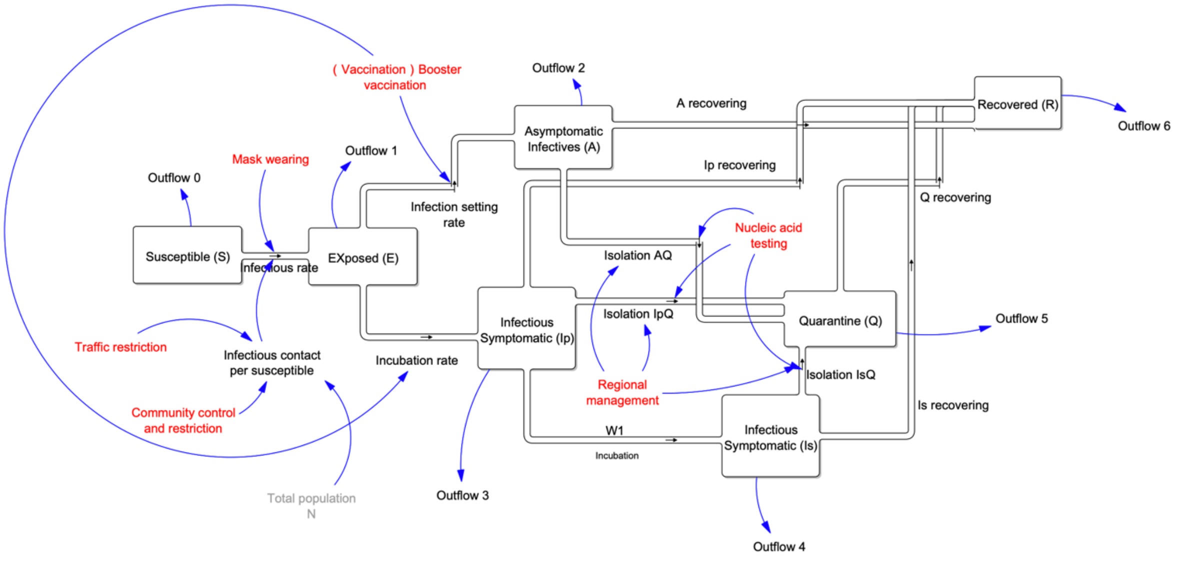

Fig.S3 The stock flow diagram of the system dynamic model for Covid-19.

(The intervention strategies are represented by causal variables shown in red color. Other causal variables, are shown in parameter tables.)

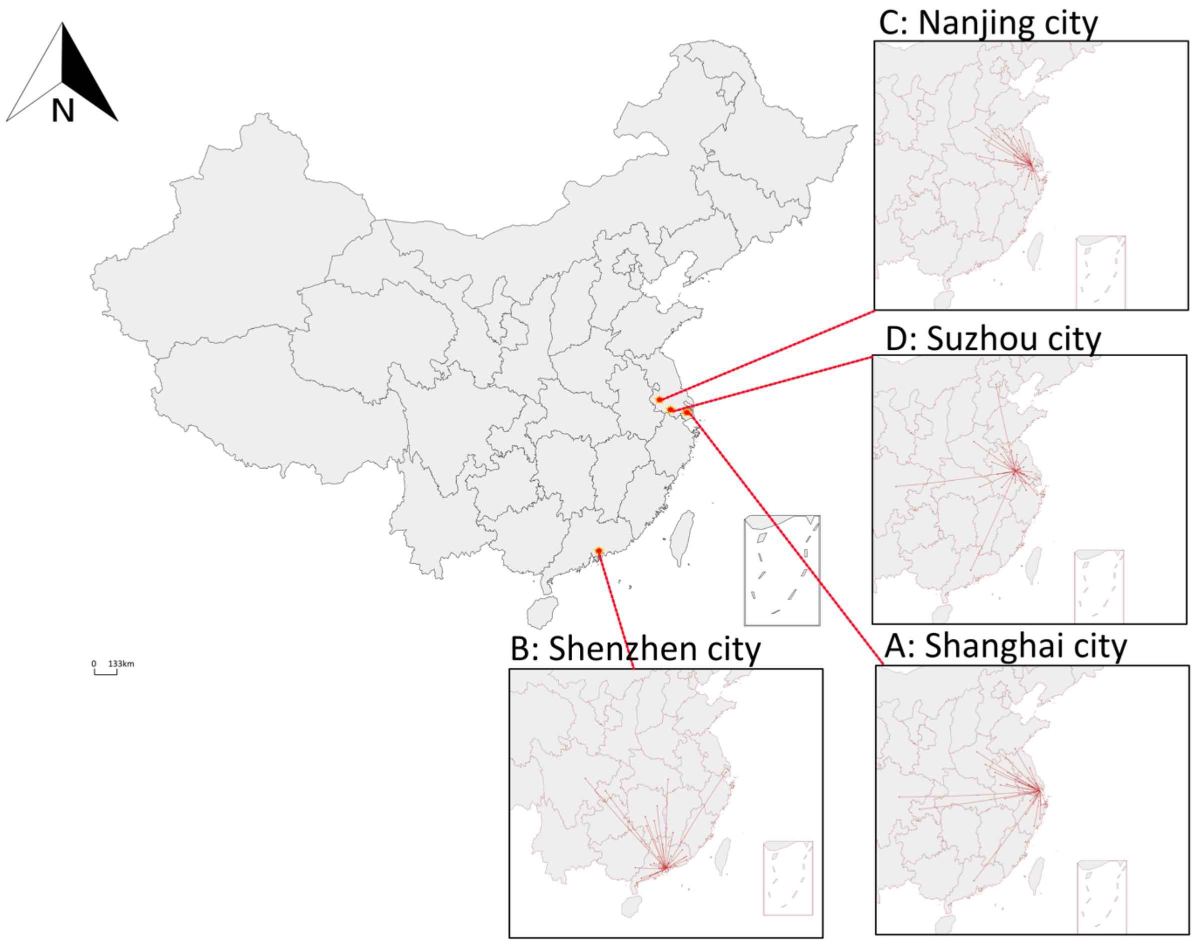

Fig.S4 Geographical distribution of the study area and importation of cases into other Chinese cities from study areas.

(Study areas include Shanghai city, Shenzhen city, Nanjing city, and Suzhou city. The red line shows the direction of spillover.)

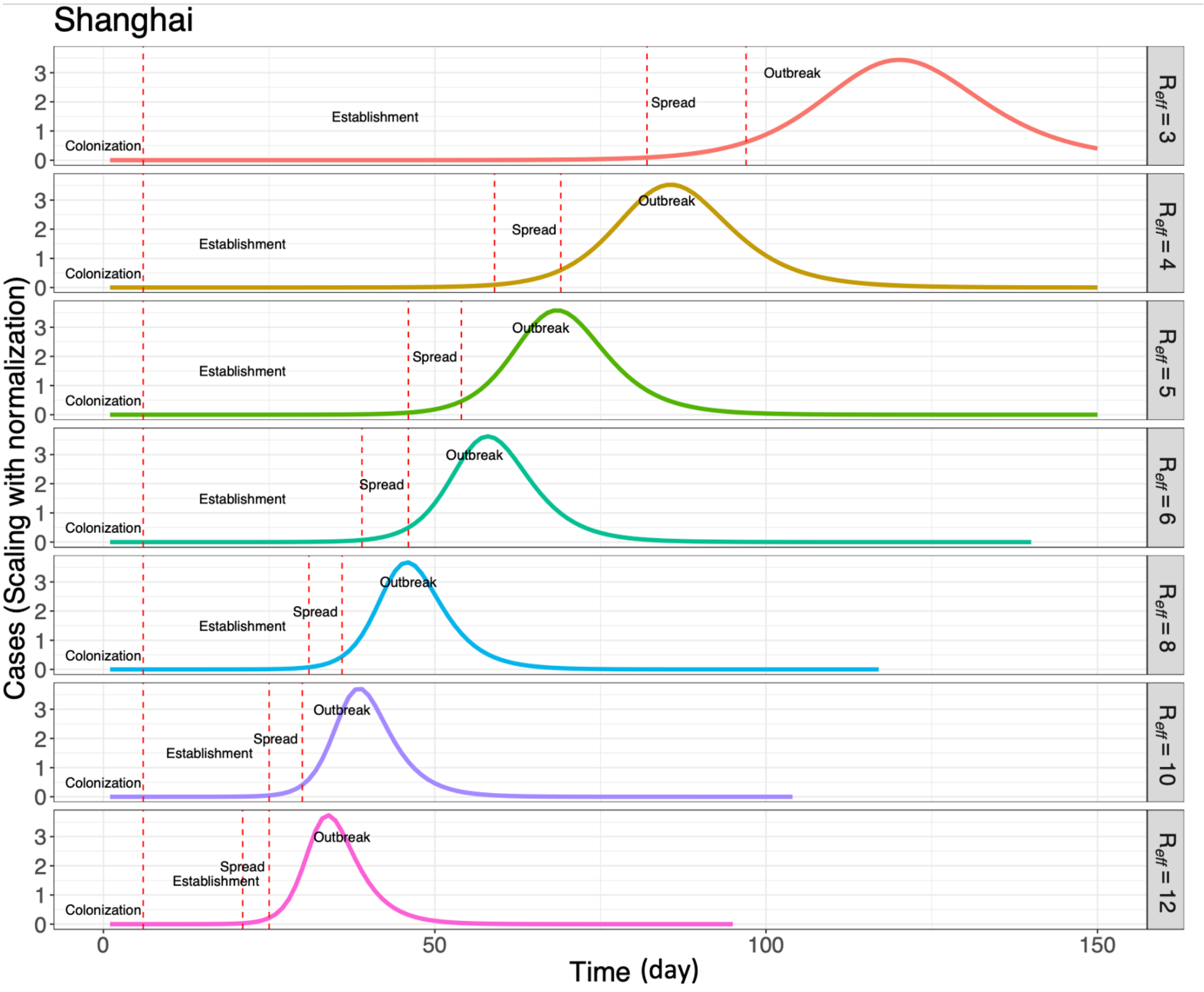

Fig.S5 Five stages of the epidemic curve based on the theory of biological invasion in Shanghai.

(Spread: Landscape spread)

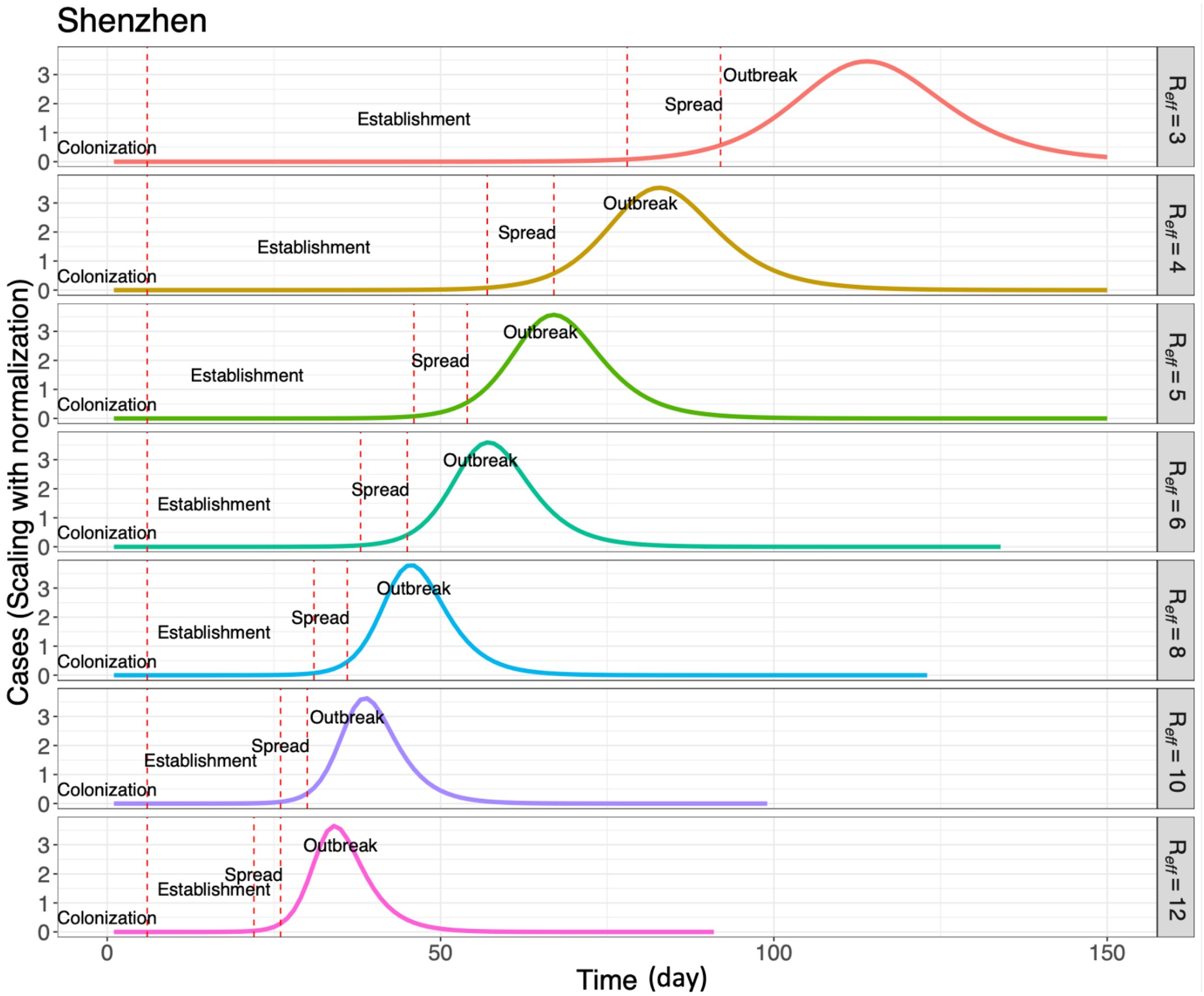

Fig.S6 Five stages of the epidemic curve based on the theory of biological invasion in Shenzhen.

(Spread: Landscape spread)

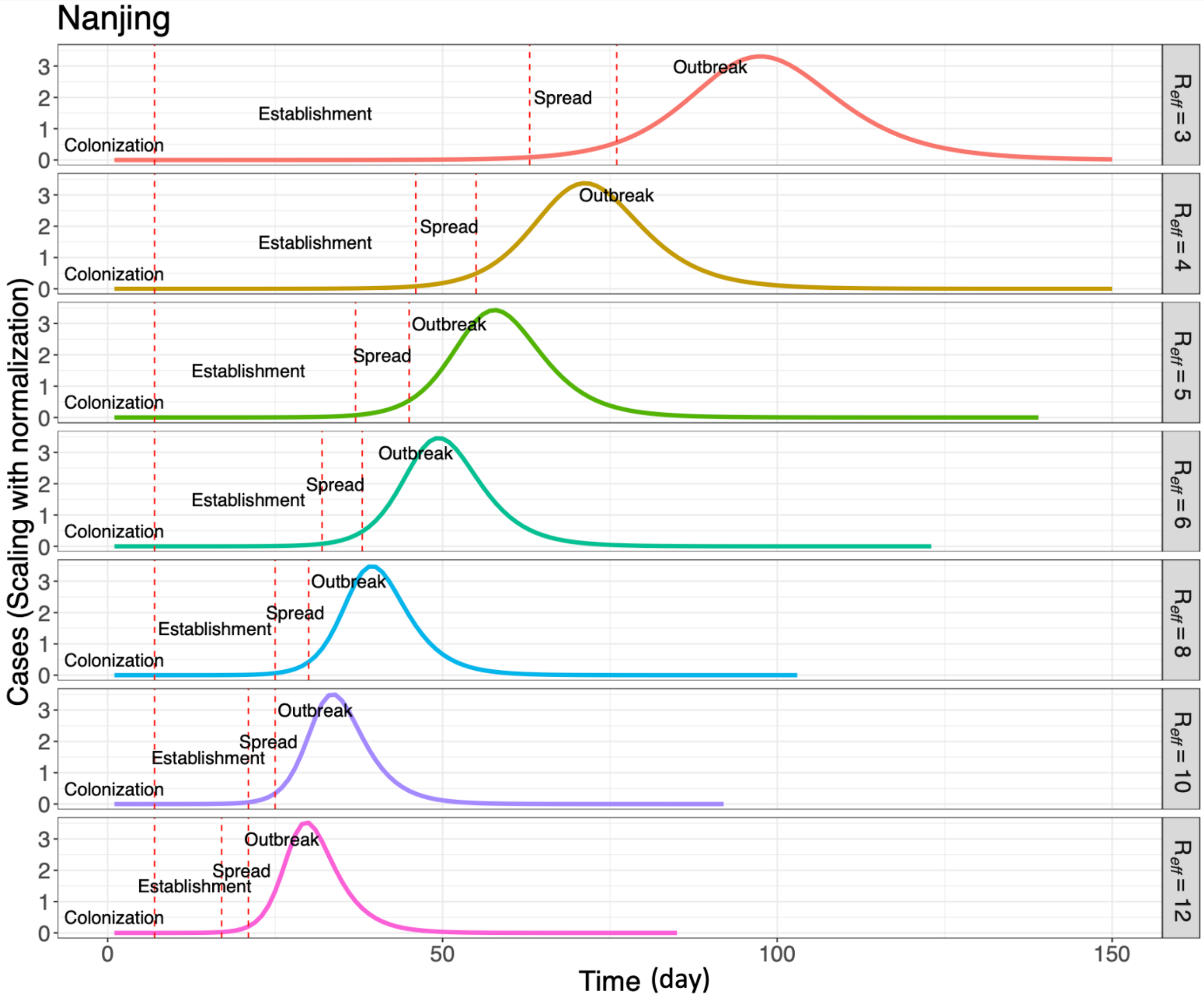

Fig.S7 Five stages of the epidemic curve based on the theory of biological invasion in Nanjing.

(Spread: Landscape spread)

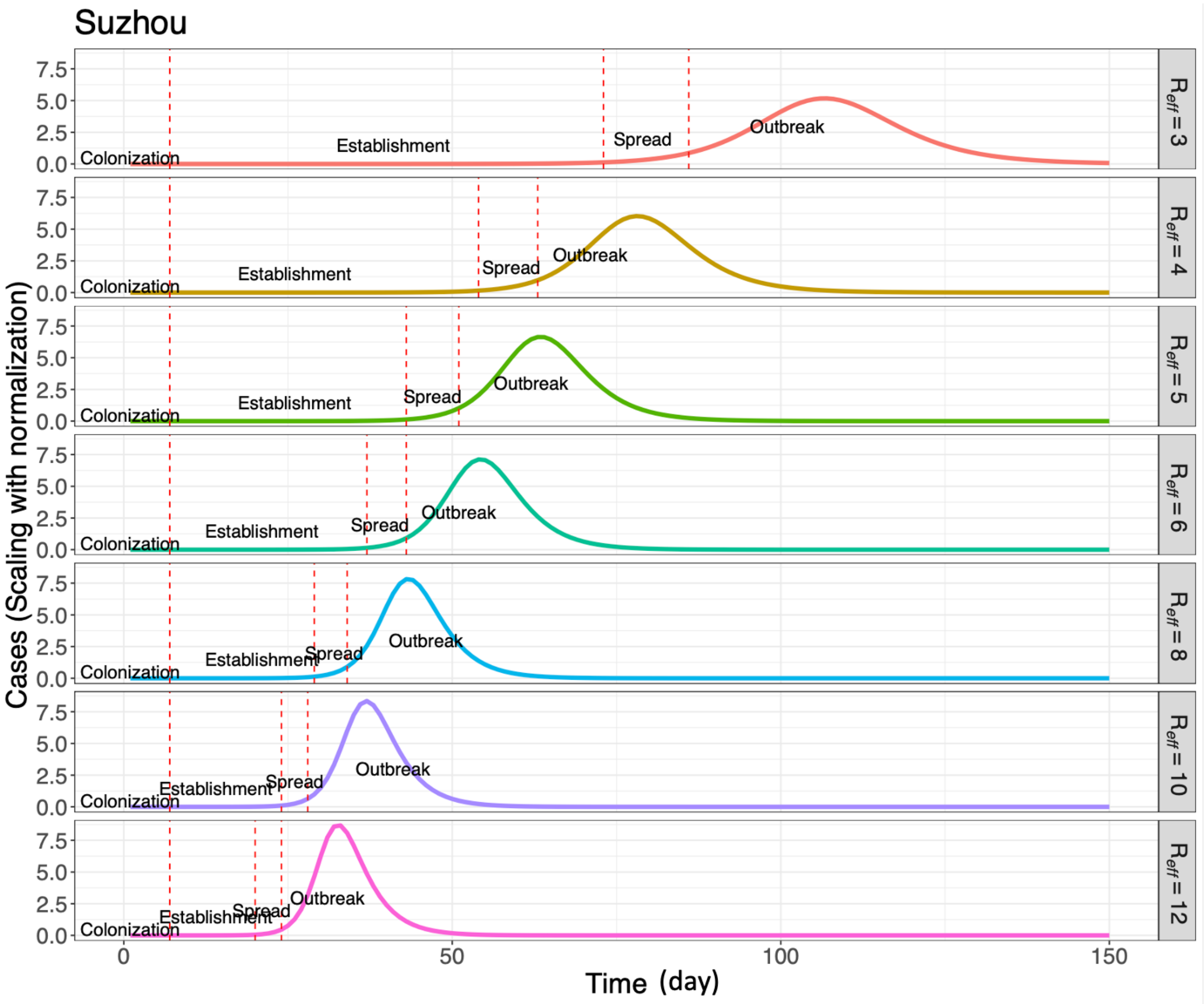

Fig.S8 Five stages of the epidemic curve based on the theory of biological invasion in Suzhou.

(Spread: Landscape spread)

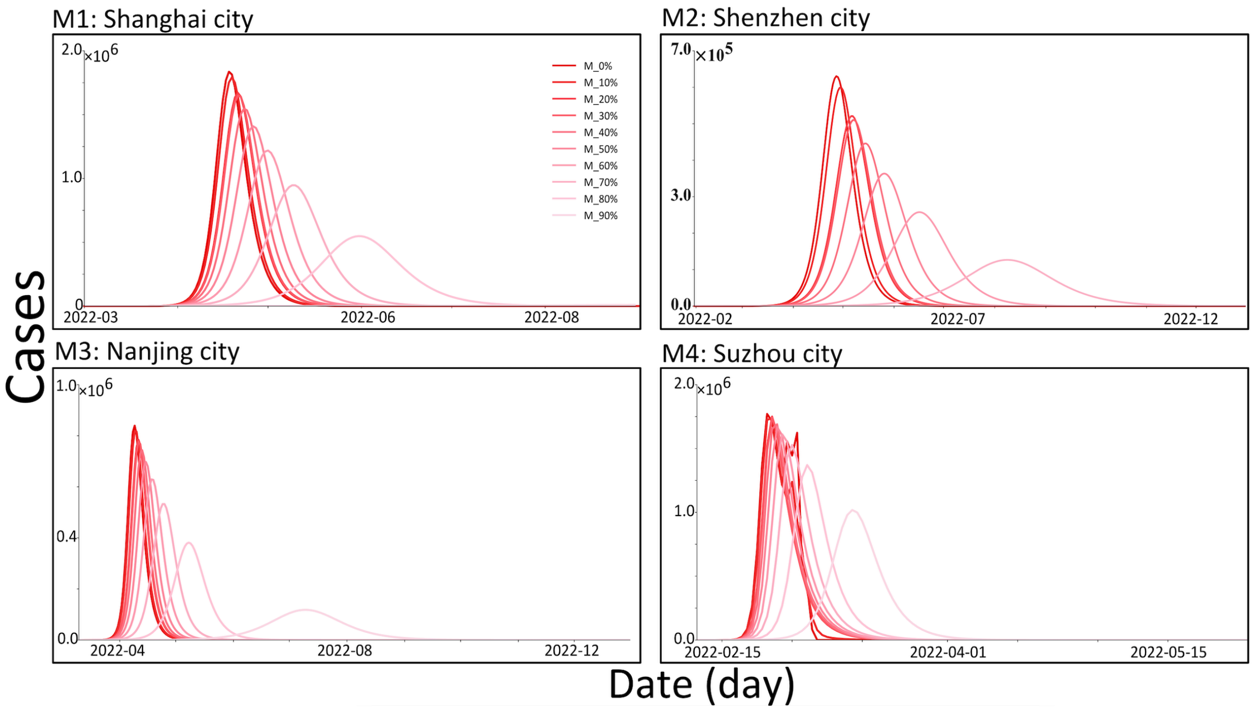

Fig.S9 Simulation of different mask wear rates in 4 cities (A total of 10 mask wear rates were simulated, ranging from 90% to 0%.)

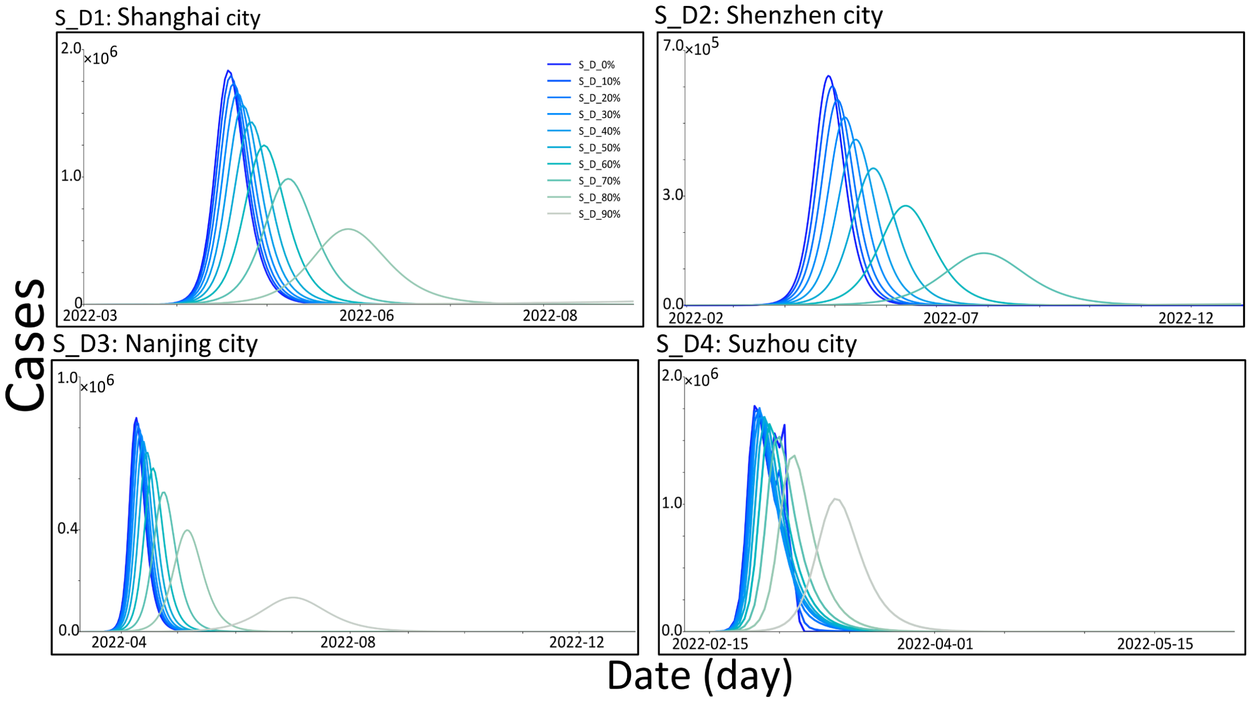

Fig.S10 Simulation of different social distances in 4 cities (A total of 10 social distances were simulated, ranging from 90% to 0%.)

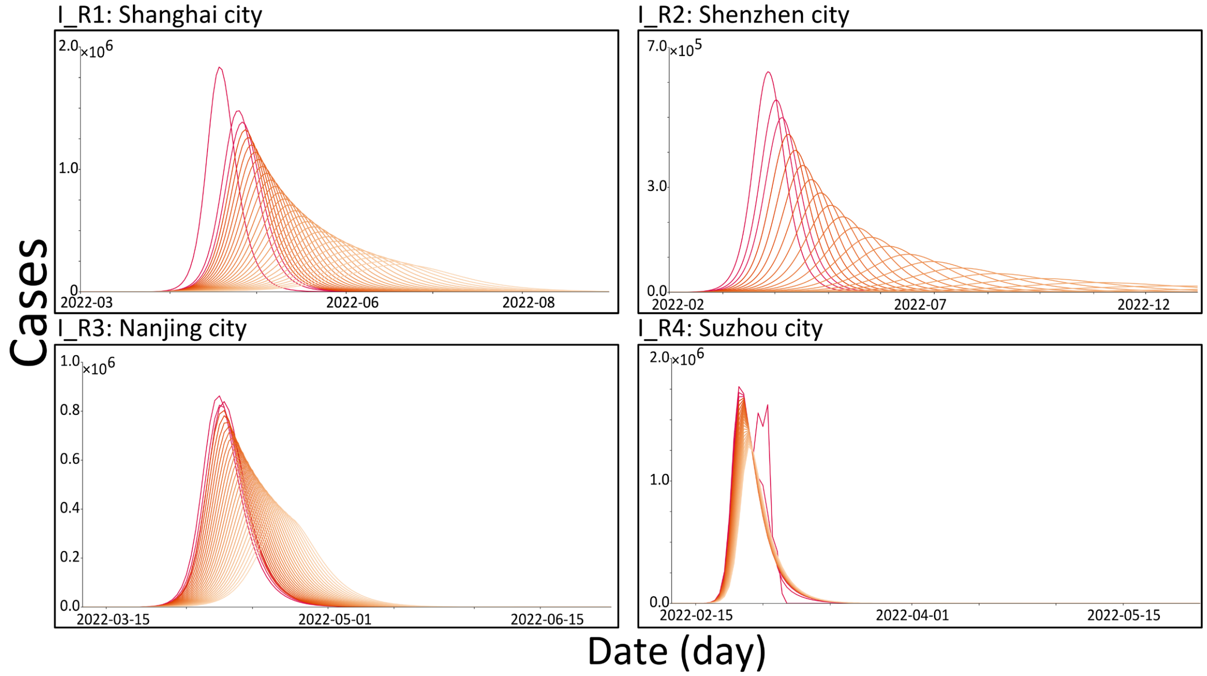

Fig.S11 Simulation of different isolation ratios in 4 cities (A total of 26 segregation ratios were modelled, ranging from 80% to 10%.)

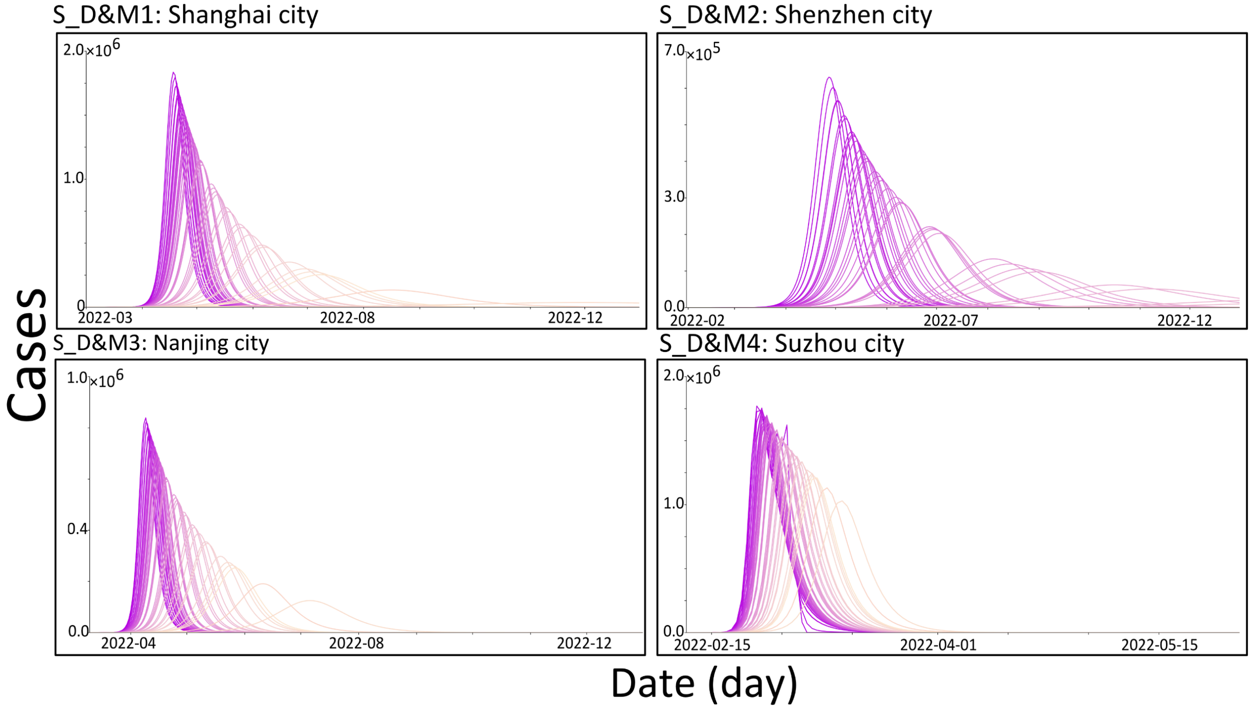

Fig.S12 Simulation of different combinations of mask wear and social distance rates in 4 cities (A total of 56 possible real-world combinations of masks + social distance measures are simulated.)

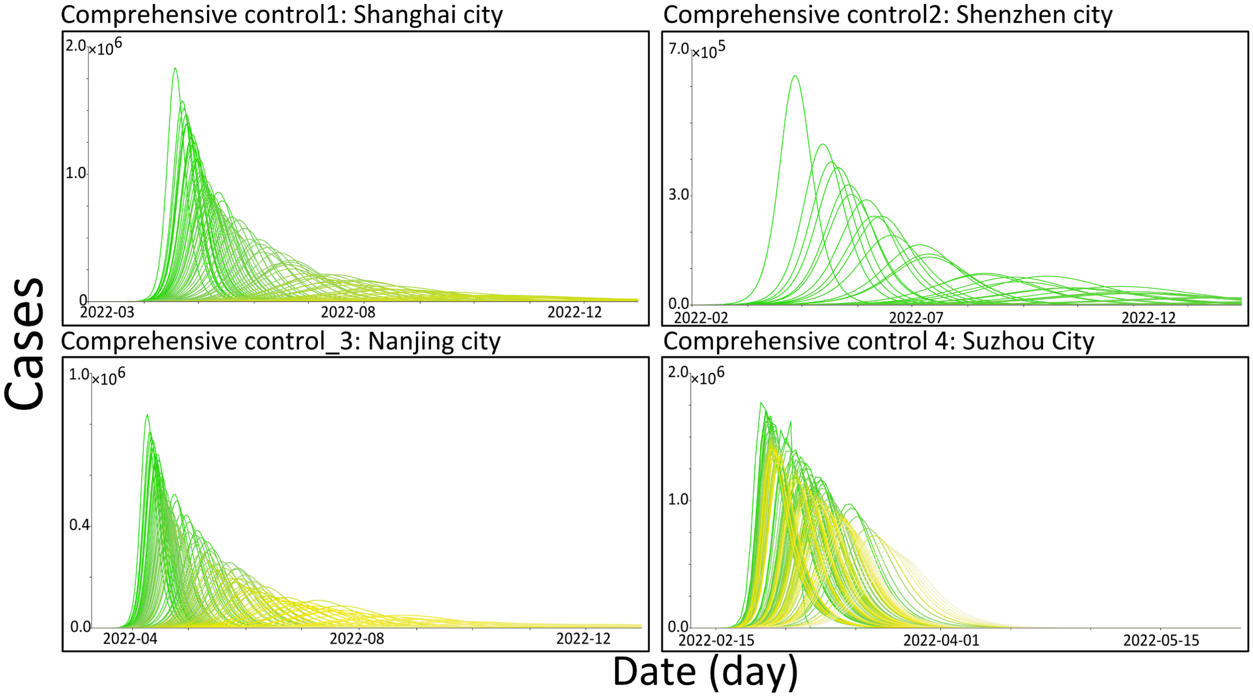

Fig.S13 Simulation of comprehensive interventions in 4 cities (A total of 100 possible real-world integrated interventions are simulated.)

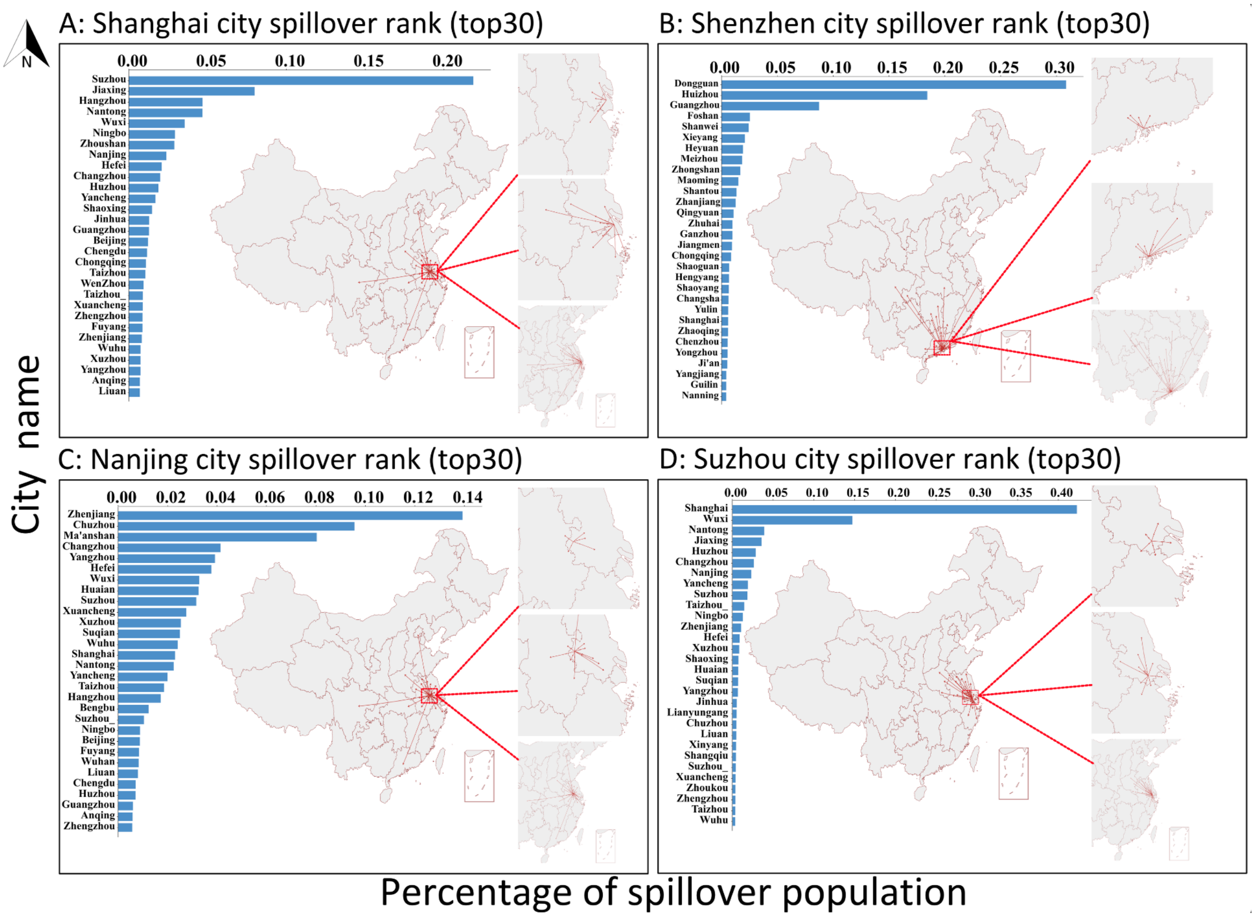

Fig.S14 Map of Spillover city top30 ranking of each city.
